# From DNA barcodes to ecology: Meta‐analysis of central European beetles reveal link with species ecology but also to data pattern and gaps

**DOI:** 10.1002/ece3.9650

**Published:** 2022-12-21

**Authors:** Sara Ottati, Jonas Eberle, Björn Rulik, Frank Köhler, Dirk Ahrens

**Affiliations:** ^1^ Zoologisches Forschungsmuseum A. Koenig (LIB) Bonn Germany; ^2^ Department of Agricultural, Forest and Food Sciences (DISAFA) University of Torino Turin Italy; ^3^ Department of Environment & Biodiversity University of Salzburg Salzburg Austria; ^4^ Coleopterological Research Office Bornheim Germany

**Keywords:** Central Europe, Coleoptera, DNA barcoding, GBOL

## Abstract

DNA barcoding has been used worldwide to identify biological specimens and to delimit species. It represents a cost‐effective, fast, and efficient way to assess biodiversity with help of the public Barcode of Life Database (BOLD) accounting for more than 236,000 animal species and more than 10 million barcode sequences. Here, we performed a meta‐analysis of available barcode data of central European Coleoptera to detect intraspecific genetic patterns among ecological groups in relation to geographic distance with the aim to investigate a possible link between infraspecific variation and species ecology. We collected information regarding feeding style, body size, as well as habitat and biotope preferences. Mantel tests and two variants of Procrustes analysis, both involving the Principal Coordinates Neighborhood Matrices (PCNM) approach, were applied on genetic and geographic distance matrices. However, significance levels were too low to further use the outcome for further trait investigation: these were in mean for all ecological guilds only 7.5, 9.4, or 15.6% for PCNM + PCA, NMDS + PCA, and Mantel test, respectively, or at best 28% for a single guild. Our study confirmed that certain ecological traits were associated with higher species diversity and foster stronger genetic differentiation. Results suggest that increased numbers of species, sampling localities, and specimens for a chosen area of interest may give new insights to explore barcode data and species ecology for the scope of conservation on a larger scale. We performed a meta‐analysis of available barcode data of central European beetles to detect intraspecific genetic patterns among ecological groups in relation to geographic distance, regarding feeding style, body size, as well as habitat and biotope preferences. Our study confirmed that certain ecological traits were associated with higher species diversity and foster stronger genetic differentiation. However, significance levels were too low to further use the outcome for further trait investigation.

## Graphical abstract

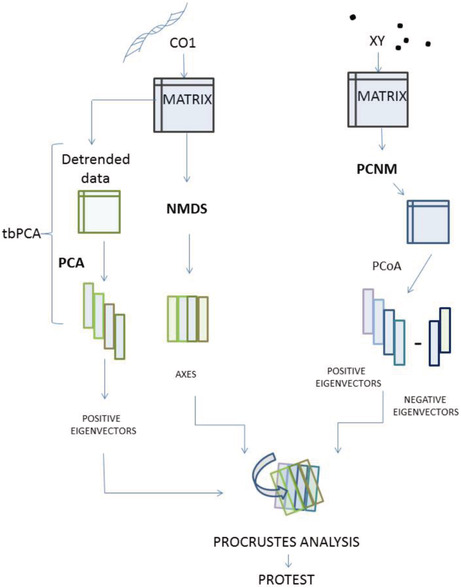

## 1 INTRODUCTION

The maintenance of biodiversity, as the sum of all organisms, communities, and ecosystems (Dirzo & Raven, 2003), is one of the most important current concerns of humankind, as wild species are decreasing at an alarming rate, and an inversion of this trend requires an anthropic intervention to guarantee their survival (Frankham et al., 2002). Biodiversity is composed by multiple dimensions, and no single measure of biodiversity can capture all its dimensions (Carpenter et al., 2009): Genetic diversity is essential in order to develop an evolutionary potential for species to be able to react to environmental changes (Toro & Caballero, 2005). A species' persistence depends on its vulnerability to these environmental changes which is determined by its genetic constitution and physiological tolerance (Potvin & Tousignant, 1996). However, very little is known on trends in genetic diversity, particularly in wild species (Pereira et al., 2012). While taxonomic coverage with indicator taxa and diversity assessments is very limited, the extinction risk of the vast majority of biodiversity is not known (Pereira et al., 2012). Thus, a characterization and management of genetic diversity seems necessary considering unique population structures, as well as to choose the correct way and the proper resolution power to estimate it (Storfer et al., 2010).

Ecological studies typically require a determination of the species involved. Acquisition of such biodiversity data for plants and animals using morphological characteristics to identify field collected samples requires both a significant time effort for identification based on morphology and sufficient taxonomic expertise that is rarely available for a vast variety of organism groups. Therefore, many ecological studies lack taxonomic information while ecological data for many species are rare.

The development of DNA‐based methods for species identification (Hebert, Cywinska, et al., 2003), has drastically simplified this identification step (Coissac et al., 2012) and might help to overcome the gap between taxonomy and ecology. DNA barcoding approaches have been widely used to explore biodiversity (e.g., Barber & Boyce, 2006; Hebert, Cywinska, et al., 2003; Hebert, Ratnasingham, & de Waard, 2003; Smith et al., 2005). Barcoding uses a single gene fragment (e.g., in most Metazoa *COI*) and, thus, through large‐scale barcoding, universal data of intraspecific genetic variation have become available over a wide range of organisms. Mitochondrial DNA (mtDNA) has been a marker of choice not only for barcoding but also for reconstructing historical patterns of population demography, admixture, biogeography, and speciation (Hurst & Jiggins, 2005). Variation in mtDNA is assessable by DNA sequencing in a cost‐effective way, which are exactly the properties that make mtDNA marker suitable for the large‐scale assessment of species boundaries (Ratnasingham & Hebert, 2007). With advances in high‐throughput sequencing methods, the production of barcodes has become increasingly more cost and time efficient (e.g., Jain et al., 2016; Srivathsan et al., 2018, 2021).

Using a standardized genetic marker in DNA barcoding allows connecting the identities of different life stages such as eggs, larvae, or adults—often a major difficulty in morphology‐based taxonomy and cryptic species (Ahrens et al., 2007; Etzler et al., 2014; García‐Robledo et al., 2013; Köhler et al., 2022; Šípek & Ahrens, 2011) and to trace in the environment remnants of organismal DNA (Taberlet et al., 2012; Yu et al., 2012). Barcoding has been successfully applied to a vast number of taxa in many different geographic regions (Ratnasingham & Hebert, 2007). It has become obvious that validated, comprehensive species libraries are the most fundamental basis for optimal barcode‐based taxon identification (Kvist, 2013). The huge amount of barcode data with large number of already collected, identified, and barcoded specimens opens up to ecologists to use these data of biodiversity in a vast geographic scale. They enhance a fast and clear overview on the biodiversity. Indeed, population genetic analysis of ecological communities with *COI* sequences extends the value of the DNA barcode employed as identification and taxonomic tool. Whereas barcoding for taxonomic purposes was in past often limited by economic constraints to a very few individuals per species, larger comprehensive studies becoming more and more available (e.g., Bergsten et al., 2012; Dincă et al., 2011; Hartop et al., 2022; Hendrich et al., 2015; Rulik et al., 2017). These data‐rich studies with multiple sampling sites may provide useful population genetic information with a link to the entire species distribution range applicable to a range of ecological and historical questions (Baselga et al., 2013, 2015; Craft et al., 2010). DNA metabarcoding, which couples the principles of DNA barcoding with next‐generation sequencing technology and often works with pooled or environmental samples (Creedy et al., 2019; Schnell et al., 2012; Taberlet et al., 2012), provides an opportunity to easily expand existing barcode library data and produce large amounts of data on biodiversity (Bush et al., 2019; Creedy et al., 2019; Elbrecht & Leese, 2015; Huang et al., 2022; Steinke et al., 2022).

In this study, we analyze samples from Central European beetle metapopulations in order to run a meta‐analysis on large‐scale barcoding data (GBOL; Hendrich et al., 2015; Rulik et al., 2017) to investigate their intraspecific degree of genetic divergence compared to their spatial and ecological properties. Based on available metadata information contained in the BOLD database, we perform a complete survey on Central European beetle fauna to explore patterns among different ecological guilds considering the spatial scale to investigate circumstances driving the intraspecific genetic (mtDNA) divergence in beetle species (Coleoptera).

## 2 MATERIAL AND METHODS

### 2.1 Data, species taxonomy and ecological information

**FIGURE 1:**
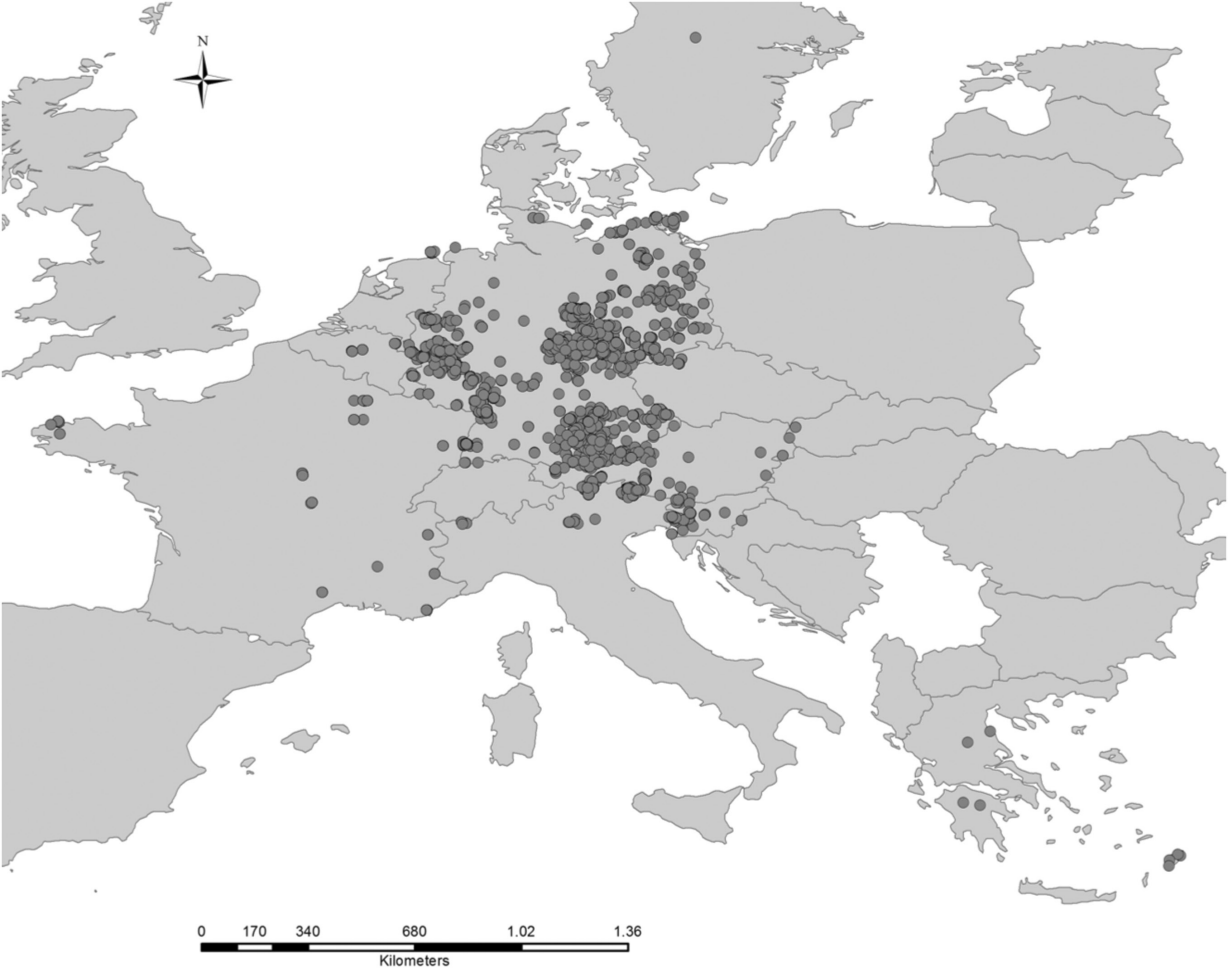
Map of the sampling sites of specimens considered for this study across the Central Europe.

The specimen's data for this study have been collected from two previous barcoding studies on central European beetles (Insecta: Coleoptera) (Hendrich et al., 2015; Rulik et al., 2017) performed in the framework of German Barcoding initiatives (i.e., German Barcode of Life project: https://www.bolgermany.de; Bavarian Barcoding project: http://www.faunabavarica.de/). These projects aimed at building a reference library of DNA barcodes for all available organisms in Germany collecting, where possible, multiple adult specimens per species, from locations as distinct as possible throughout the Germany and the neighboring countries in order to capture genetic variability (Figure 1). Final data assembly was based on the combined dataset of Rulik et al. (2017) which had dealt carefully with contaminated samples and misidentifications.

In order to avoid an underestimation of intraspecific genetic differences and to maximize the amount of complete available data from BOLD (http://www.barcodinglife.org), we excluded from the analysis redundant genetic information represented by identical syntopic haplotypes with equal geographical coordinates and incomplete ecological or geographical information on the specimens. From these, we retained only those species for which were available comprehensive ecological information (see below).

Species taxonomy used as backbone for this study is derived from Köhler and Klausnitzer (1998) and subsequent works (Bleich et al., 2016; Köhler, 2000, 2011a, 2011b) reflecting the current species taxonomy applied in German coleopterist's community (http://www.coleokat.de/de/fhl/ (status: 2016)). Eventual inconsistencies of current classification with the source of ecological data (Koch, 1989, 1991, 1992) were adopted by F. K. in his curated database with help of the numerical identifier for each species (Lohse & Lucht, 1999). For our meta‐analysis, we selected among available data to examine the effect of four major ecological variables (body size, biotype preference, habitat preference, and feeding habits) in the context of geographical distance and genetic (mtDNA) differentiation.

For the autecological information on the species (Table S1), which we regard here as a proxy for the entire species ecology, we used data on feeding habits, habitat preference, biotope preferences, and body size (derived from a data base curated by F. K. based mainly on Koch, 1989, 1991, 1992 and many further, more detailed publications not mentioned here). These data are mostly based on adult specimen observations from faunistic literature and records since larvae are only rarely observed or identified. Feeding habits included eight different, more generic classes: coprophagous, polyphagous, mycetophagous, necrophagous, phytophagous, saprophagous, xylophagous, and zoophagous (Figure 3b). While the same number of classes was used for the habitat preference: soil; eurytop, rotting matters, nests, mushrooms, vegetation, water, and dead wood (Figure 3a), the biotope preferences were represented by four different categories: no biotope preference, wetlands, open‐land biotypes, and forests (Figure 3c). Five size classes were arbitrary defined: 0–2 mm, 2–5 mm, 5–10 mm, 10–20 mm, 20–50 mm. These reflect roughly eco‐functional groupings in the food web due to body size. However, therefore, the ecological classes are not equally represented among the sampled species (Figure 3d).

### 2.2 Metadata analysis

**FIGURE 2:**
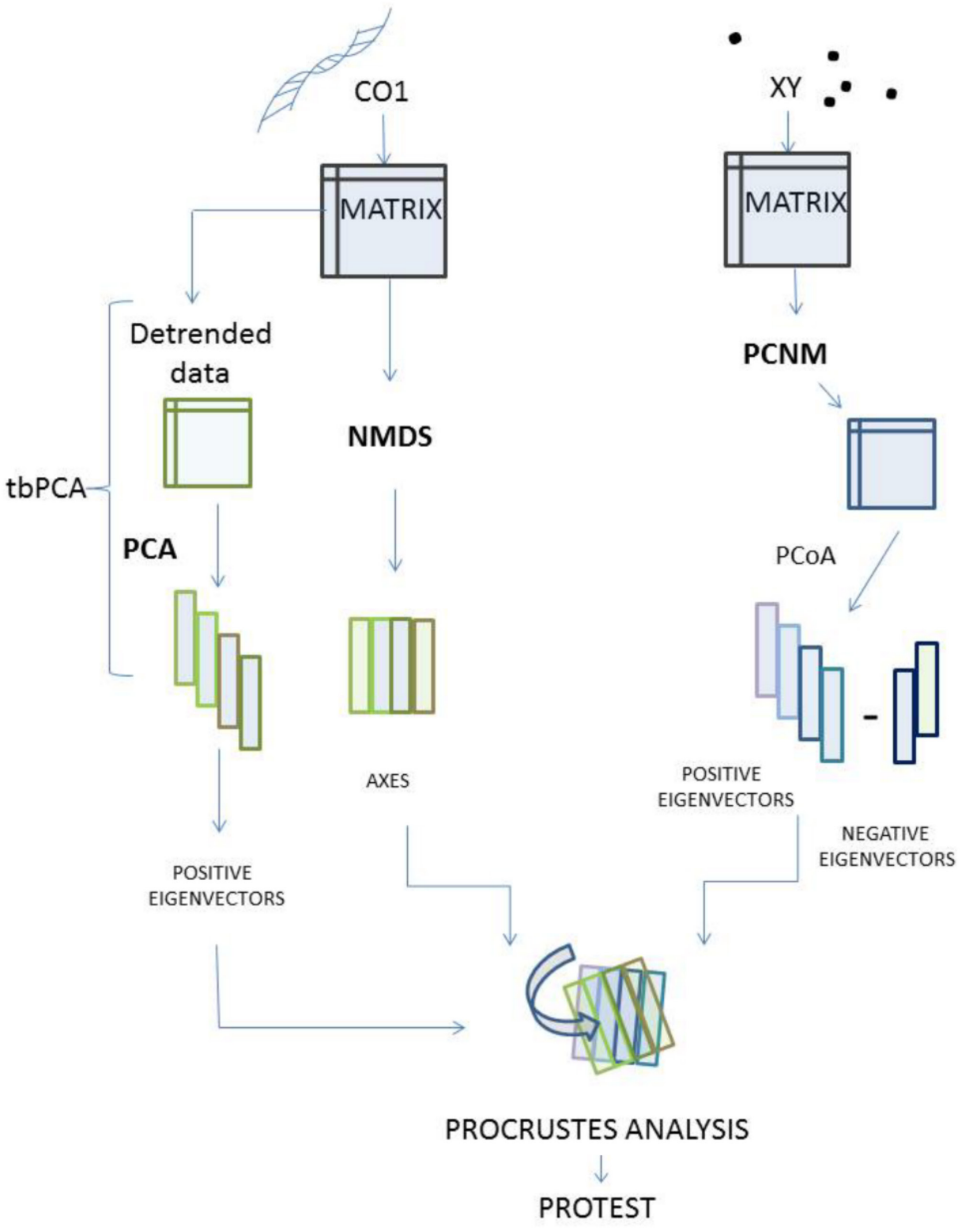
Flow chart illustrating the two alternative analyses to the Mantel test used to detect the intraspecific relationship between genetic distances (*COI*) and geographical distances (XY) starting from pairwise distance matrices of samples. Left side: PCA method; middle: The NMDS method; it starts with the same matrix and ends with a set of axes representing the DNA distances. Right side: PCNM technique performed on geographic coordinates. The PCNM produced a set of eigenvectors which had been regressed in Procrustes superimposition analysis first against NMDS axis, then against PCA eigenvectors. Finally, we performed a significance test with Protest (Jackson, 1995).

In order to inquire which ecological species properties influence the intraspecific genetic differentiation, we investigated for correlation between infraspecific genetic distances of *COI* barcode data (5′ end; i.e., HCO/LCO region) and geographical distances among infraspecific samples using three different approaches (Figure 2, see below). Due to some controversy in the scientific community about Mantel test (Legendre et al., 2015; Peres‐Neto & Jackson, 2001), we included here also ordination techniques (Legendre & Legendre, 2012) and combined these to the method of principal coordinates of neighbor matrices in a Procrustes analysis (PCNM; Borcard et al., 2004; Borcard & Legendre, 2002; Dray et al., 2006). In a second step, we linked the outcome of these comparisons to the ecological properties for each species. Significance is expected to be in major part is also dependent from intraspecific sampling of each species which is in many species rather limited. The sheer amount of available data nevertheless promises sufficient significant results.

To investigate the impact of sampling area size on the results, a fifth categorical, sampling‐dependent variable has been introduced: the distance classes which represent the average distance between all individuals of the same species. Geographical distances were calculated based on the geographical coordinates of the barcoded specimens used in this study (Hendrich et al., 2015; Rulik et al., 2017; see BOLD repositories https://doi.org/10.5883/ds‐gcolzfmk and https://doi.org/10.5883/DS‐COLBYGER). A high distance class is equivalent with a larger sampling area of a species. The distance class thus represents the distance in which most of the sampled specimens were found. All mean distances within 100 km were in the distance class “100,” all those within 101 and 150 km were in the distance class “150,” those between 151 and 200 km in class “200,” to class “300” belong all those species ranging between 201 and 300 km and the last class includes all the species with a mean geographic distance higher than 301 km.

The meta‐analysis was performed using R v. 3.4.3 (R Core Team, 2017) in R‐studio v. 0.99.878 (Rstudio Team, 2016). Euclidean distance matrices from the set of geographical coordinates of collection sites of each specimen were generated with *geosphere* package (Hijmans, 2016). Pairwise distances from *COI* sequences using the Kimura 2‐parameters DNA substitution model (Kimura, 1980) were calculated in the *ape* package (Version 4.1; Paradis et al., 2004). Even there was some controversy about the use of Kimura 2‐parameters as DNA substitution model in barcodes (Magnacca & Brown, 2010; Moniz & Kaczmarska, 2010; Srivathsan & Meier, 2012), we use this substitution model as recommended by Hebert, Cywinska, et al. (2003) and Hebert, Ratnasingham, and de Waard (2003) since this study inquires at “population” level.

On these two distance matrices, we subsequently performed the following three different analyses to infer the number of species of each ecological class whose intraspecific genetic distances were correlated with the geographical distances.

Initially, we used a Mantel test (Mantel, 1967) which calculates the correlation between values in the corresponding positions of two matrices. Significance of the linear relationship between matrices is assessed through permutation of objects (Peres‐Neto & Jackson, 2001). Being first applied in population genetics by Sokal (1979), the Mantel test is currently one of the most commonly used methods to evaluate the relationship between geographic distance and genetic divergence (Mantel, 1967; see Diniz‐Filho et al., 2013). Despite recent controversy and criticism about its statistical performance (e.g., Castellano & Balletto, 2002; Guillot & Rousset, 2013; Harmon & Glor, 2010; Legendre & Fortin, 2010) and the existence of more sophisticated and complex approaches to analyze spatial multivariate data (Diniz‐Filho et al., 2013), the Mantel test is still one of the most employed methods in matrix data correlations. We ran the Mantel test performing 10,000 permutations on submatrices, each one representing a single species composed from more than four specimens. The null hypothesis, i.e., the absence of relationship, was rejected when the p value was lower than 0.05. To run these analyses, we used the function “mantel()” in the *vegan* package (Version: 2.6‐4; Oksanen et al., 2017) where the Mantel statistics was defined as a matrix correlation between the two, geographical and genetic, dissimilarity matrices.

Alternatively, we ran two variants of Procrustes analyses (Figure 2) in which the genetic distances were treated in different ways. The geographical distance matrix was transformed using principal coordinates of neighbor matrices (PCNM) (Borcard et al., 2004; Borcard & Legendre, 2002; Dray et al., 2006) implementing the spatial Eigenfunction analysis (SEA) (Manel et al., 2003; Manel & Holderegger, 2013) and principal coordinates analysis (PcoA). For PCNM, we used the tool in the *vegan* package that automatically sets the threshold distance above distances which are simply considered “large”. Any geographical distance above this value was set to four times the threshold value (see Borcard & Legendre, 2002). This modified distance matrix is then subject to principal coordinates analysis (PCoA). All resulting eigenvectors with positive eigenvalues have been used as a new set of explanatory spatial variables in a multiple regression approach trough Procrustes superimposition method.

The genetic distance matrix was analyzed with a transformation‐based principal components analysis (Tb‐PCA) or with NMDS. The transformation‐based principal components analysis (Tb‐PCA) is considered to be the two‐step equivalent of the principal coordinates analysis (PcoA) which includes a detrending procedure (Legendre & Gallagher, 2001). To detrend the data, we used (decostand() function in “vegan” package) before performing the PCA. Statistical significance in these comparisons indicates strength of evidence about the probable trend of differentiation of the populations involved (Allendorf & Luikart, 2007). Again, only the resulting positive eigenvectors have been combined with PCNM eigenvectors in a Procrustes analysis, which aims to find the species where the two distance matrices are better correlated.

We have chosen the Procrustes superimposition technique because variables could be either ordination axes (usually those that explain most of the variation in a data set) or original variables; it may also be applied to (dis)similarity matrices describing the same objects. In contrast to the Mantel test, Procrustes analysis allows to determine how much variance in one matrix is attributable to the variance in the other. Finally, we performed linear regressions one by one within the same species on the sets of axes representing genetic distances against the PCNM axes representing the geographic distances to test for significance of the relationship between the two variables (PROTEST, Jackson, 1995, Figure 2). PCNM axes were computed both times using pcnm() function in *vegan* package (Oksanen et al., 2017), while tb‐PCA and NMDS axes were computed, respectively, using the pcoa() function in *vegan* and the nmds() function in *ecodist* package (Version: 1.2.9‐1; Goslee & Urban, 2007). Regarding the results of Procrustes analysis, we refer in the following text (statistics and significance values) to “NMDS statistics,” “NMDS significance,” or generally to “NMDS results” for results that were computed using regressing NMDS and PCNM axes, while we use “PCA statistics,” “PCA significance,” and “PCA results” for results referring to the Procrustes output involving tb‐PCA axes and PCNM eigenvectors.

### 2.3 Role of ecological characteristics

Pairwise intraspecific geographical and genetic distances were plotted in subsets of ecological properties (see above). Subsequently, sampling variables (i.e., the number of sampled localities, the number of sampled specimens and the mean geographic distance among sampled individuals per species) were plotted against test statistics and significance to investigate the degree of their correlation. To check for the role of sampling design on the meta‐analyses through plot choice, we compared statistics and significance scores of every species against the other variables with the number of individuals examined per species, the number of sampled localities and the mean geographic distance among sampled individuals per species. To detect potential population genetic “anomalies” or “regularities” referable to ecological dynamics, we produced boxplots on all data and on subsets of data, observing the variance in the mean values of statistic and significance scores obtained as Mantel/NMDS/PCA output.

## 3 RESULTS

Of the 29,464 available *COI* barcode sequences, geographical coordinates were available for 29,349 individuals (Figure 1). Excluding identical syntopic haplotypes and individuals with incomplete geographical coordinates, we retained for the analysis 16,283 individuals of 3967 species from 124 Coleoptera families. From these, we retained only those species for which comprehensive ecological information was available, such as feeding style, habitat and biotope preference, and body size resulting in 12,207 specimens available for final meta‐analysis representing 1785 species and 95 families of Coleoptera from Germany. The largest geographical distance value was found to be 719 km (*Longitarsus parvulus* Paykull, 1799) and the smallest was about 5 km (*Chrysolina marginata* Linnaeus, 1758).

The largest number of species (>28%; *n* > 500) was found in the distance class “300,” the fewest species were in the class “>300” (Figure S1). About half of all recovered species were sampled from few localities only (≤6) (Figure S1). Most species were zoophagous (37%; *n* = 674) or phytophagous (*n* = 585), while 35% of all species were associated with vegetation as their habitat (*n* = 632). Dead wood and soil habitats accounted for 22% (*n* = 398 and 400, respectively) (Figure S2). Across the preferred biotopes, 38% of the species (*n* = 691) preferred forest, many others (23%; *n* = 420) either open land or wetland (20%; *n* = 369). 17% (*n* = 305) had not specific preferences (Figure S2). The most represented size‐classes were, as expected, very small (33% of all species; *n* = 594) and small (35%; *n* = 626) species. The medium size class accounted for 21% of all species (*n* = 386), while the large and largest beetles counted 9% and 157 and 22 species, respectively (Figure S2).

**FIGURE 3:**
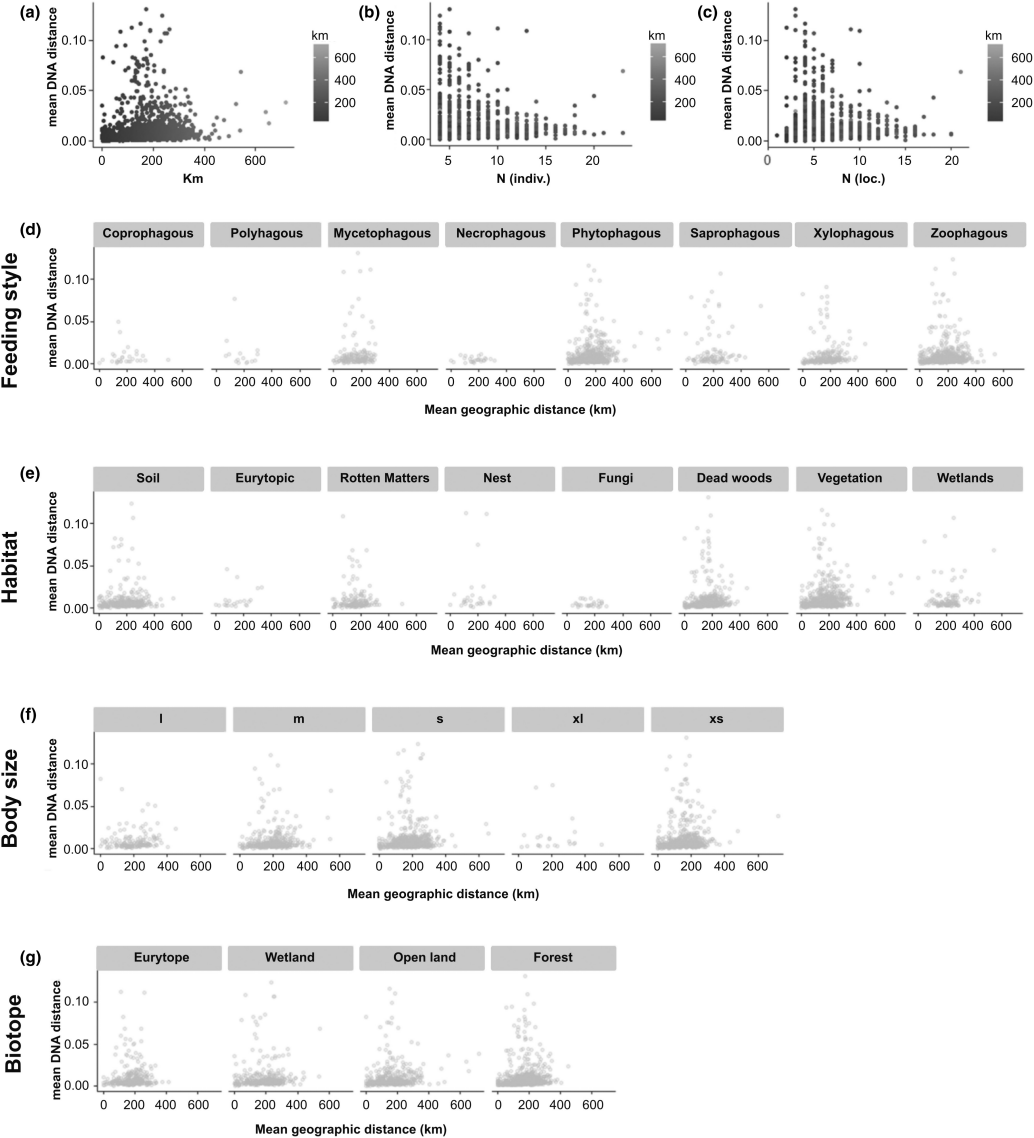
Mean intraspecific genetic distances plotted vs a) mean geographic intraspecific distances (in km); (b) number of sampled individuals per species (N_ind); (c) number of sampling sites per species (loc); (d) vs geographic distances (km) for guilds with different feeding styles; (e) vs geographic distances (km) for guilds with different habitat preferences; (f) vs geographic distances (km) for body size classes; (g) vs geographic distances (km) for guilds of biotope preference.

Infraspecific genetic distances resulted to be inversely proportional to the number of sampled individuals and number of sampling localities (Figure 3b, c), while intraspecific geographic distances were not affected by the restricted sampling (in the original design of sampling had an upper limit for infraspecific samples to due limited budget of the barcoding projects). Despite numerous cases with elevated infraspecific divergence, distance plots across the four major eco‐classes of central European beetles (habitat, feeding preferences, biotope, and size class) revealed generally low intraspecific distances (<3% sequence divergence). Patterns of geographical and genetic differentiation differed between some of the ecological and eco‐morphological traits. Indeed, the plots (all data) of geographical distance matrices versus genetic matrices showed in some cases different data distributions across different ecological guilds (Figure 3d–g, Figure S3), particularly for habitat and feeding style. For the biotope types, no major differences were visually evident (Figure 3g), although in biotopes, wetland, and open land more higher distances were occurring than among forest and eurytopic biotopes. We also observed a trend that with the increase of body size (from extra‐small to extra‐large), the infraspecific genetic distances decreased (Figure 3f, Figure S3C). Larger geographical distances occurred in saprophagous and phytophagous taxa or species preferring wetland and vegetation, for which specimens had been also sequenced from outside of Germany (Figure S3). In saprophagous taxa, a large amount of pairwise distances of specimens followed a nearly proportional increase in both genetic and geographic distances. The same was found for the xylophagous and zoophagous species. A different pattern was evident in mycetophagous species in which genetic distances were already much higher even with short geographic distances. In necrophagous, polyphagous, and coprophagous species, we observed the opposite (with some minor exceptions), i.e., a clearly limited amount of genetic variation even with increased geographic distance (Figure 3d). In regard of habitat preferences, vegetation, rotten matters, and dead woods had similar trends as taxa with phyto‐, sapro‐, and xylophagous feeding style, obviously due to the connection between these eco‐classes. Species with habitats such as soil and nests had also a higher genetic variation at same geographical scale (i.e., distance class), while others such as the “mushrooms/ fungi” inhabitants had comparatively lower genetic structure despite a vast geographical sampling (Figure 3e).

All the three methods of correlation analysis between genetic and geographical distances detected cases, in which the expected relationship between geographical and genetic distances was confirmed. Here all correlation approaches were positive (Figures S4, S5). The Mantel test identified 250 species having a significant relationship between the two distances (14% of the total number of examined species), NMDS 160 species (9% of the total number of examined species) and PCA 123 (6.8% of the total number of examined species). Overlap of significant species between NMDS and Mantels test was 9.2%, while species overlap of PCA with Mantel test was 9.6% of and 32.5% with NMDS. This apparent discordance in identifying the significant species with correlated patterns was evident from pairwise plots of significance and statistic values of each species from the three methods (Figure S6): while the PCA and NMDS methods agreed in both significance and statistic values in most of the cases, both methods highly diverged with the mantel resulting scores. Furthermore, maximum and minimal values of significant species differed between the three methods (Table 1).

**TABLE 1:** Number and their respective percentages (%) of species resulting to be significant in the correlation analysis of infraspecific distances vs. geographic distances, as performed by the Mantel test, NMDS + PCA, and PCNM + PCA (in comparison to the total species number of each category; *N*_spec_), respective selected life traits of the species (feedings style, habitat, body size, and biotope).

	*N*_spec_	Mantel	%	NMDS + PCA	%	PCNM + PCA	%
Feeding style							
Coprophagous	29	8	27.59	6	20.69	5	17.24
Polyphagous	15	4	26.67	3	20.00	1	6.67
Mycetophagous	156	27.3	17.50	14	8.97	13	8.33
Necrophagous	27	3	11.11	0	0	1	3.70
Phytophgous	585	80	13.68	60	10.26	40	6.84
Saprophagous	99	17	17.17	9	9.09	8	8.08
Xylophagous	200	32.3	16.15	19	9.50	14	7.00
Zoophagous	674	101	14.99	55	8.16	39	5.79
Habitat							
Soil	398	60	15.08	36	9.05	26	6.53
Eurytopic	22	3	13.64	0	0	0	0
Rotten matters	167	21	12.57	12	7.19	9	5.39
Nest	28	2	7.14	4	14.29	3	10.71
Mushrooms	29	5	17.24	1	3.45	2	6.90
Dead woods	404	41	10.15	39	9.65	32	7.92
Vegetation	632	88	13.92	68	10.76	41	6.49
Wetlands	105	30	28.57	7	6.67	8	7.62
Biotope							
Eurytope	305	41	13.44	22	7.21	17	5.57
Wetland	369	75	20.33	30	8.13	26	7.05
Open land	420	56	13.33	52	12.38	30	7.14
Forest	691	78	11.29	63	9.12	48	6.95
Body size							
xs	594	77	12.96	47	7.91	33	5.56
s	626	99	15.81	63	10.06	49	7.83
m	386	53	13.73	40	10.36	22	5.70
l	157	18	11.46	15	9.55	13	8.28
xl	22	3	13.64	3	13.64	4	18.18
Overall mean			15.57		9.44		7.50

Significance for the ecological traits and their sub‐guilds was rather rare in all three approaches of correlation analysis (Figure S5). Significance levels were too low to further use the outcome for further going trait investigation. These were in mean for all ecological guilds 7.5, 9.4, or 15.6% for PCNM + PCA, NMDS + PCA, and Mantel test, respectively, or at best 28% for a single guild (Table 2). Only few families provided significant results (Figure S7). All three correlation tests found coprophagous species to have relatively more species with significant correlation between genetic and geographic distances (Tables 1 and 2). While the mantel test identified nest dwellers as having the lowest percentage of significances, the opposite was the case for NMDS and PCNM in which the nest dweller guild was the most dominant in significance. PCA and NMDS recorded high percentages in significant species in nest habitat and body size (i.e., extra‐large), while small species were those dominant for the Mantel test.

**TABLE 2:** Comparison between the three analytic methods.

ANOVA	Mantel			NMDS + PCA			PCNM + PCA		
	Statistic	Signifi‐cance	Binary sig.^a^	Statistic	Signifi‐cance	Binary sig.	Statistic	Signifi‐cance	Binary sig.
Feeding style	–	3.5	–	–	–	–	–	–	–
Coprophagous (i)	0.21	0.4	−6.51	0.67	0.57	−10.39	0.73	0.49	−23.25
Polyphagous	–	–	–	–	–	–	–	–	–
Mycetophagous	–	–	–	–	–	–	–	–	–
Necrophagous	–	–	–	–	–	–	–	–	–
Phytophagous	–	–	–	–	–	–	–	–	–
Saprophagous	–	–	–	–	–	–	–	–	–
Xylophagous	–	–	−1.33	–	–	–	–	–	–
Zoophagous	–	–	–	–	–	–	–	–	–
Habitat	6.9	4.9	–	2.8	2.9	–	–	–	–
Soil (i)	0.27	0.41	−7.57	0.64	0.52	−11.17	0.7	0.52	−8.04
Eurytopic	–	–	–	–	–	–	–	–	–
Rotten matters	–	–	–	–	–	–	–	–	–
Nest	–	–	–	–	–	–	–	–	–
Mushrooms	–	–	–	0.08	−0.11	–	–	–	–
Dead woods	−0.06	0.05	–	–	–	–	–	–	–
Vegetation	−0.06	–	–	0.03	−0.05	–	0.02	−0.04	–
Wetlands	0.11	−0.07	–	–	–	–	–	–	–
Biotope	19.9	17.1	–	–	3	–	–	2.6	–
Eurytopic (i)	0.2	0.46	−6.93	0.64	0.52	−11.15	0.7	0.52	−23.91
Wetland	0.1	−0.05	–	–	–	–	–	–	–
Open land	–	–	–	–	–	–	–	−0.04	–
Forest	–	–	−0.62	–	–	–	–	−0.04	–
Body size	–	–	–	–	–	–	–	–	–
l (i)	0.22	0.44	−7.5	0.65	0.5	−10.98	0.69	0.52	−23.63
m	–	–	–	–	–	–	–	–	−0.91
s	–	–	–	–	–	–	–	–	–
xl	–	–	–	–	–	–	–	–	–
xs	–	–	–	–	–	–	–	–	–
Distance classes	8	4.2	–	3.5	4.9	–	16.4	2.3	–
>300 (i)	0.21	0.54	−7.85	0.62	0.53	−10.69	0.69	0.53	−25.1
100	–	–	–	–	–	–	–	–	–
150	–	–	–	–	–	–	–	–	–
200	–	–	–	–	–	–	–	–	–
300	–	–	–	–	–	–	–	–	–

^a^ NA is reported in correspondence of the GLM predictor (Binary sig.) as no analysis of variance had been computed for such categorical variable.

Only significant scores resulting from the multilinear models are reported. The first row of each eco‐class shows the *F*‐statistic score (when significant) for the correspondent pair of variables. A dash indicates no support by the models (i‐intercept).

## 4 DISCUSSION

Our investigation of the genetic vs. geographic distances within species across multiple guilds of different ecological traits and taxa (95 Coleoptera families) revealed that there are only few cases in which this relationship resulted to be significant and thus predictable. In these cases, an increase in geographic intraspecific distances was associated with increased genetic distances, or the other way around, and they were sufficiently sampled (in terms of number of samples to examine) to support the trends statistically. The observed patterns of infraspecific genetic and geographical distances among most of the central European Coleoptera species and ecological guilds examined were, however, neither uniform nor entirely different among each other. Thus, the unlikely hypothesis that species generally increase their genetic diversity with the distance of their record, which could be interpreted as a signal of gradual dispersion and genetic differentiation in progress (Allendorf & Luikart, 2007), could be not confirmed. Yet, the study area suffered an almost entire biodiversity loss during the Pleistocene and was reoccupied afterward from external founder populations (e.g., Abellán et al., 2011; Birks & Tinner, 2016; Hewitt, 2000; Hofreiter & Stewart, 2009).

Besides insufficient sampling, cases, in which species did not show a positive relation between genetic and geographical distances, might be explained in two ways: (1) the geographic expansion of the species was not followed by a genetic diversification (intraspecific DNA distances are smaller than geographic distances). This case might occur in species with high dispersal capabilities with continuous genetic mixing and in a well interconnected area or (2) the genetic diversification in the study area was independent from the geographic scale, occurring mainly in species with a potentially different phylogeographic origin of their populations (refugia). It is well known that Central Europe experienced post‐glacial recolonization events from different Mediterranean and extra‐Mediterranean refugia located all across the continent (e.g., Ahrens et al., 2013; Kühne et al., 2017; Schmitt & Varga, 2012). Freijeiro and Baselga (2016) suggested that dispersal‐based processes in European beetles were probably taxon‐dependent, but also depended on dispersal ability and ecological traits (Gómez‐Rodríguez et al., 2015). Although patterns appear not very clear due to widely lacking significance in our data, we found several patterns in genetic/geographic relationships among ecological preference classes which might fit a more ecology‐dependent dispersal and differentiation (Papadopoulou et al., 2008). Indeed, causalities are expected to depend on both environmental and ecological processes in the species range. The distribution area as well as the relation of genetic distance vs. geographic distance in a species depends on several factors: the paleo‐biogeographic and biogeographic history (i.e., glacial expansion dynamics, glacial refugia presence, postglacial climatic gradients, and postglacial species expansion) (Stewart et al., 2010). It is widely accepted that present distribution patterns in Central Europe, besides other also important factors (Baselga et al., 2012), are related to post glacial recolonization dynamics (e.g., Schmitt, 2009; Schuldt & Assmann, 2011). In this context, geographic, climatic, and ecological exogenous factors (i.e., climatic gradients, habitat fragmentation, or presence/absence of corridors) and ecological endogenous factors (i.e., potential niche or dispersal capabilities due to physiological properties, level of adaptation) may play a crucial role to determine these patterns (e.g., Rundell & Price, 2009; Schmitt, 2009). So far, distribution patterns and genetic differentiation have been studied mainly for selected cases (taxa or/and study sites) in the framework of phylogeographic studies (e.g., Domènech et al., 2022; Garcia‐Raventós et al., 2021; Múrria et al., 2020). Only some studies with wider taxonomic and geographical scope do exist (e.g., Baselga et al., 2013, 2015; Dapporto et al., 2019; Fujisawa et al., 2014; Joly et al., 2014). With the upcoming barcode data, a vast amount of data is becoming available to address such questions routinely at large scale, and to uncover particularly responses at population level regarding many ecological and climatic factors which have so far been explored with limited systematic sampling.

Here, deeper going conclusions lack statistic support. This is basically due to the fact that barcoding campaigns were generally not designed to explore phylogeographic patterns in the context of species ecology. At this stage, we expect (yet) sampling bias since data were generated with the scope of collecting and barcoding as many species as possible for future DNA‐based species identification. However, the amount of available data on central European beetle species appeared valuable as starting point to enquire the relationships between ecological properties of the species and their intraspecific patterns of genetic differentiation and also to look at patterns that go beyond a single guild or species group (Baselga et al., 2013, 2015; Fujisawa et al., 2014). In fact, we faced severe problems on our analysis due to the quite limited number of available sampling localities and of individuals per species.

PCA and NMDS techniques captured different information compared to the Mantel test. Thus, the PCA technique was the least efficient in describing the relation of genetic and geographic distances in terms of amount of resulting significant species, followed by NMDS method and Mantel test. Results of both were similar but partially different from those obtained from Mantel test (Figure S5). This discordance limited even more our general conclusions. It is likely that the different algorithms behind the analytical techniques behaved differently in the presence of high level of noise in the data caused by spatial autocorrelation (Diniz‐Filho et al., 2003; Legendre et al., 2015; although not tested here) and lacking sufficient geographical sampling (see above).

However, because different methods may emphasize different aspects of the data, using different data analyses techniques (Figure 2) may reveal more and possibly alternative aspects of the data structure than a single method (Kenkel & Orloci, 1986). The Mantel test is considered to handle the limited number of available samples per species best. Being first applied in population genetics by Sokal (1979), the Mantel test is currently one of the most commonly used methods to evaluate the relationship between geographic distance and genetic divergence (Mantel, 1967; see Diniz‐Filho et al., 2013; Manly, 1985, 1997)—despite recent controversy and criticism about its statistical performance (e.g., Castellano & Balletto, 2002; Guillot & Rousset, 2013; Harmon & Glor, 2010; Legendre & Fortin, 2010) and the existence of more sophisticated and complex approaches to analyze spatial multivariate data (Diniz‐Filho et al., 2013).

The limited number of available samples was rather disadvantageous for the ordination techniques which better read and converted the data matrices in more readable and efficient row data (Legendre et al., 2015) for the further Procrustes analysis. Ordination techniques are known to work better when dealing with big amount of data. On the other hand, minimal sampling size in our data was below the generally suggested amount to robustly investigate phenomena depending on spatial scale (at least 20 sampling localities; Dale & Fortin, 2014). In our case study, the mean number was only five sampling localities per species. Even though ordination methods are better suited, less prone to type I error and better in describing patterns (Legendre et al., 2015; Legendre & Fortin, 2010; Wang et al., 2010, 2013), results were here not congruent with those of the Mantel test. Nevertheless, PCNM methods combined to genetic information should be considered an alternative to the Mantel test and further analysis on a richer data set could then possibly lead to clearer ecological conclusions.

However, it is known from literature that the occupied habitat type has significant effects on both extent of the species range and latitudinal distribution (Fujisawa et al., 2014; Hof et al., 2006; Ribera & Vogler, 2000). This extends by some aspects the results of Fujisawa et al. (2014) who found infraspecific genetic variation of *COI* in water beetles positively correlated with occupancy (numbers of sites of species presence, i.e., a similar but not identical measure to geographical distance) and negatively with latitude; substitution rates across species (which we did not examine here) were influenced mainly by habitat types. Specialized species that occurred in more stable environments (e.g., running water) had the highest rate. Baselga et al. (2015) expected dispersal to be high in aquatic beetles (of standing waters) because of the need for movement between ephemeral water bodies, while dispersal of leaf beetles does not require long‐range movement for population persistence due to more stable conditions and generally wide expansion of vegetation. This is also reflected by our findings for the species using vegetation as habitat. Our data thus seem to confirm the habitat stability hypothesis (Ribera et al., 2003). The latter sees in Pleistocene glacial events and the following climatic stability the major causes in producing equilibrium conditions, either with environmental factors due to niche‐based processes or with spatial distributions from long‐term stochastic dispersal.

**FIGURE 4:**
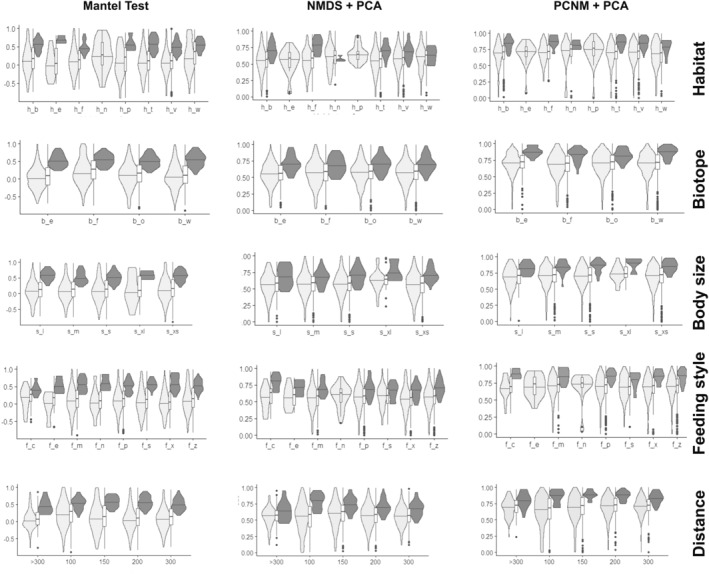
Violin plots and bar plots of statistic scores from Mantel, NMDS + PCA, and PCNM + PCA analyses across the different ecological guilds and subcategories, differentiated for species with significant (dark gray) and nonsignificant (light gray) correlation. Habitat preferences: h_b = soil, h_e = eurytop, h_f = rotting matters, h_n = nest, h_p = vegetation, h_t = dead wood, h_v = vegetation; h_w = water; biotope preferences: b_e = eurytopic; b_f = wetlands; b_o = open‐land biotypes; b_w = forests; body size classes: s_xs = extra small; s_s = small; s_m = medium; s_l = large and s_xl = extra‐large; feeding style: f_c = coprophagous; f_e = polyphagous; f_m = mycetophagous; f_n = necrophagous; f_p = phytophagous; f_s = saprophagous; f_x = xylophagous; f_z = zoophagous; geographical distance classes: highest threshold measured in km.

Our results supported higher dispersal tendencies and lower infraspecific variation of mtDNA for more ephemeral food resources (dung, dead animals), or habitats (fungi/ mushrooms) (see also Baselga et al., 2015). However, low number of species in these guilds and a similar pattern for eurytopic species (Table 1) might indicate that this observed pattern could be also a result of sampling bias (i.e., the limited number of sample sites). Specimens' body size does not provide an answer to this question, as generally divergent patterns of infraspecific genetic vs geographical distances between smaller (x_s, s, m) and larger species (l, x_l) (Figure 3) are contrasted by the rather uniform correlation statistics between the size classes (Figure 4). Studies on ground beetles, however, have shown a generally higher genetic diversity even across larger species independent from their sample site distance (Assmann et al., 2010; Schuldt & Assmann, 2011). This pattern is explained by these authors by the lack of interconnection among populations due to their very specific habitat requirements, the habitat quality, and respective morphological adaptations (e.g., wing reduction; Jelaska & Durbešić, 2009). Indeed, habitat fragmentation is considered a major factor limiting gene flow in ground beetle populations (Liebherr, 1988). Consequently, the presence or absence of wings is an important factor for a better understanding of the geographical/genetic scale relationship (Freijeiro & Baselga, 2016).

Beside yet enormous sampling gaps and underrepresentation of many species, our results indicate that genetic differentiation reflects ecological niches and habitat preferences as well as past geographic dispersal. These results can be extremely helpful to further develop conservation strategies, from a simple species conservation approach toward the conservation of genetic diversity in habitats or landscapes (e.g., Hedrick, 2001; Vellend et al., 2014). Therefore, further molecular screening would be useful, particularly focusing on more intense and wider geographical sampling to cover more in detail the genetic variation within the study area (or a species range) and to uncover causalities of such patterns (i.e., extending the Barcoding toward population level). Indeed, our results showed that an increase in number of sampling localities was usually followed by a related increase in statistical significance and thus increase the explanatory power of barcode data to explain infraspecific genetic patterns among different ecological guilds.

The screening at the diversity patterns of the entire entomological fauna in such vast territory as Central Europe is a demanding task that requires efficiency and great sampling efforts. In the light of the emergency of current trends of insect decline (e.g., Hallmann et al., 2017; Wagner et al., 2021), it also becomes an important issue for deeper understanding of its causes. The German Barcoding of Life campaign (Hendrich et al., 2015; Rulik et al., 2017) and resulting database contributed to resolve some of these issues. A denser geographic sampling that will hopefully also result from future monitoring studies or metabarcoding projects will enhance the number of sampled localities and specimens, while concerted actions and good practice of documenting those records (maintaining vouchers!) would be desirable. This will in future allow bolder conclusions regarding biodiversity in a study area rather than simple species numbers.

## AUTHOR CONTRIBUTIONS

**Sara Ottati:** Conceptualization (equal); data curation (equal); formal analysis (equal); investigation (equal); methodology (equal); software (equal); visualization (equal); writing – original draft (equal); writing – review and editing (equal). **Jonas Eberle:** Conceptualization (equal); formal analysis (equal); methodology (equal); software (equal); supervision (equal); validation (equal); writing – original draft (equal); writing – review and editing (equal). **Frank Köhler:** Data curation (equal); resources (equal); validation (equal); writing – review and editing (equal). **Björn Rulik:** Data curation (equal); formal analysis (equal); funding acquisition (equal); investigation (equal); methodology (equal); project administration (equal); resources (equal); software (equal); supervision (equal); validation (equal); writing – review and editing (equal). **Dirk Ahrens:** Conceptualization (equal); funding acquisition (equal); investigation (equal); methodology (equal); resources (equal); supervision (equal); validation (equal); writing – original draft (equal); writing – review and editing (equal).

## Supporting information


Figures S1‐S7

Table S1

## Data Availability

The molecular data that support the findings of this study are openly available in BOLD, NCBI and GBOL. The ecologcial metadata that support the findings of this study are available on request from F.K. The data are not publicly available due to privacy or ethical restrictions.
