# Supplementary material for: From DNA barcodes to ecology: Meta‐analysis of central European beetles reveal link with species ecology but also to data pattern and gaps
Source: Ecol Evol. 2022 Dec 21;12(12):e9650. doi: 10.1002/ece3.9650 (PMC9771709; doi:10.1002/ece3.9650)
Supplement: Supplementary file 1 — Figures S1‐S7 [file ECE3-12-e9650-s002.pdf]

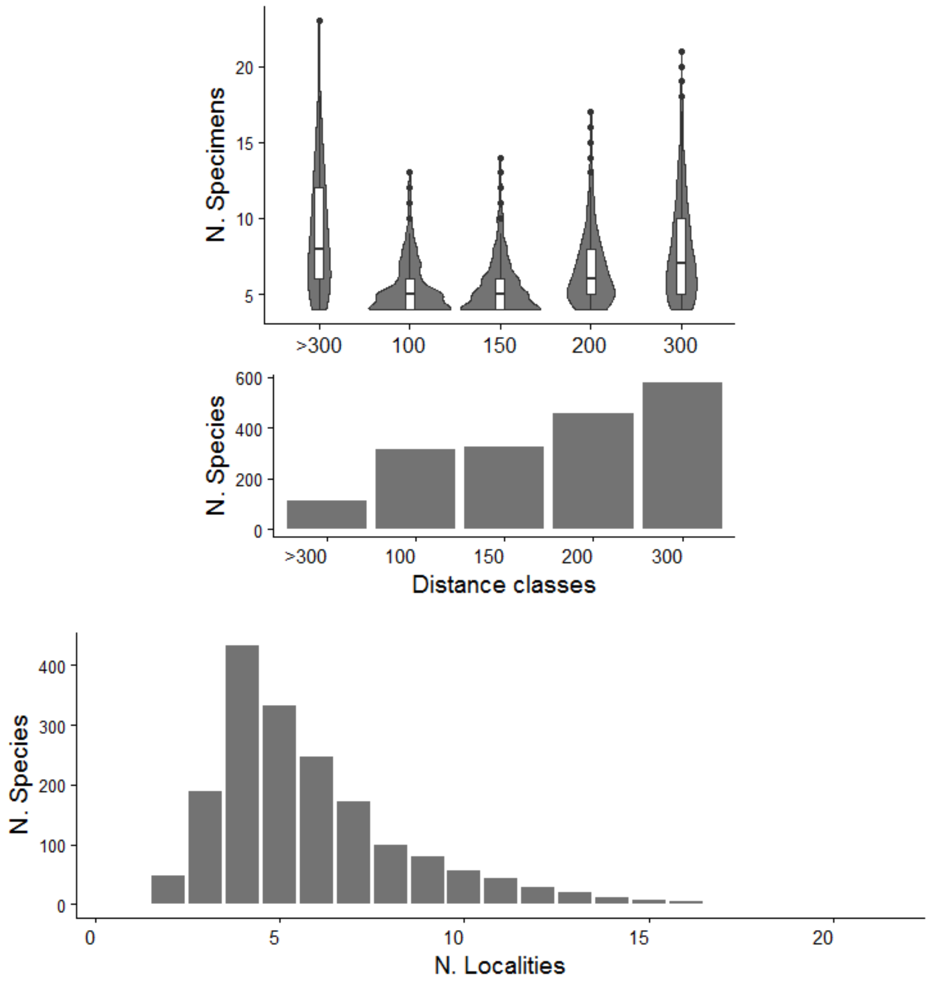

Supplement Figure 1. Distribution of the number of specimens (above) and species (middle) on the five classes of geographical distances and the amount of sampling localities (below).

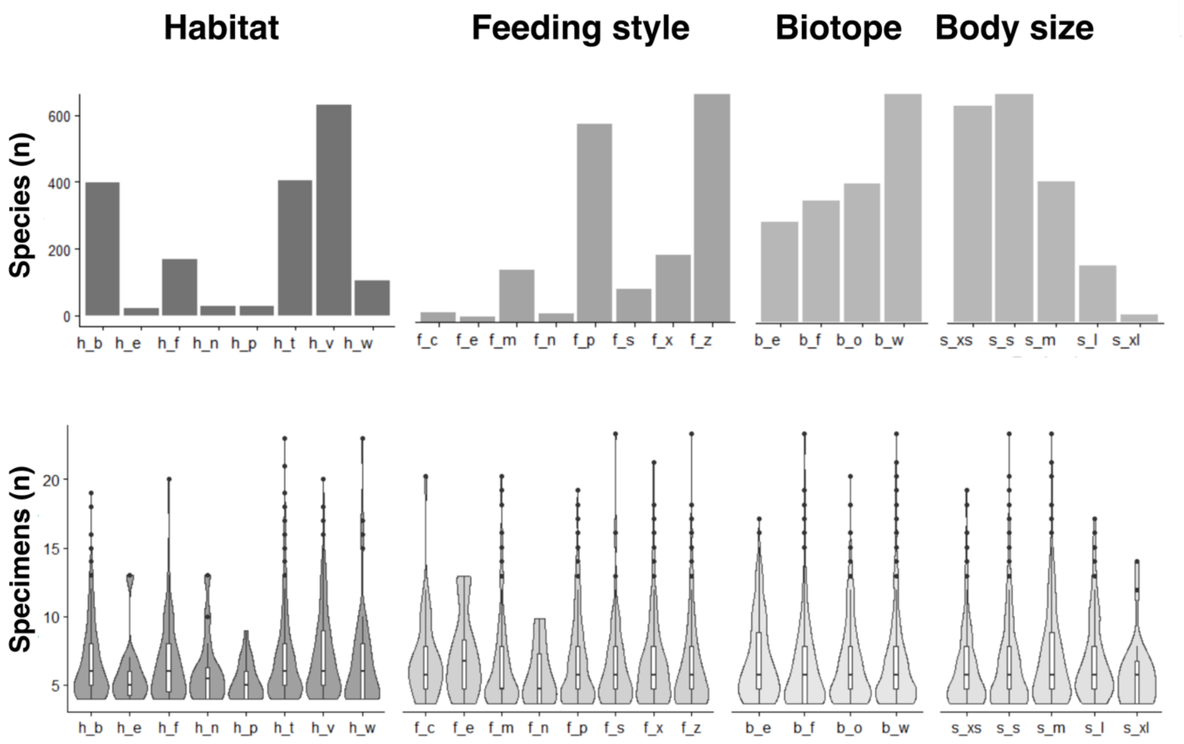

Supplement Figure 2. Distribution of number of species (bar plots, above) and specimens (violin plots, below) across the ecological guilds with major ecological characteristics, such as habitat preference, feeding style, biotope preference, and body size classes. The width of violin indicates the total number of such cases.

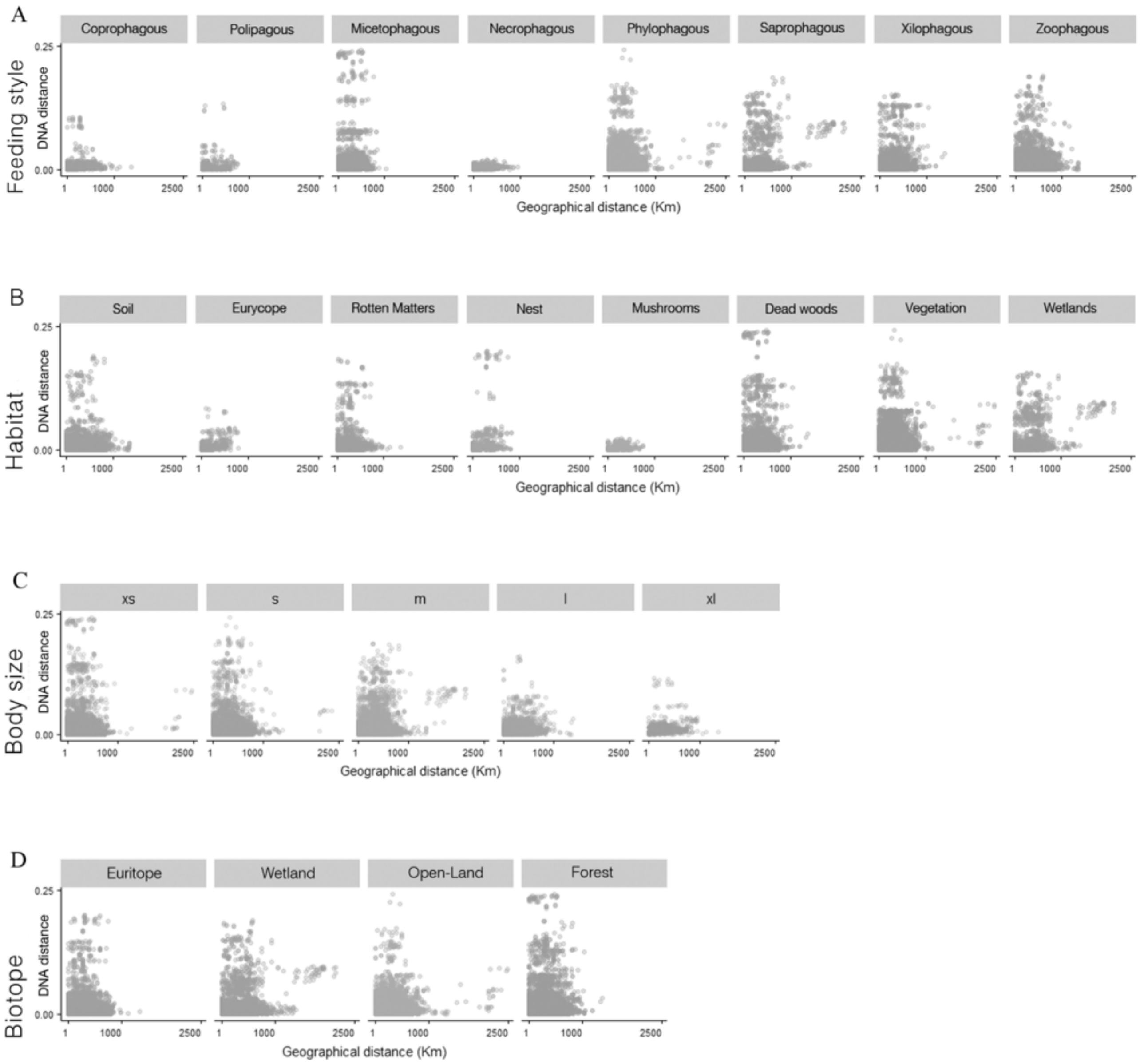

Supplement Figure 3. Relationships between pairwise geographic and genetic distances across guilds for each ecological variable.

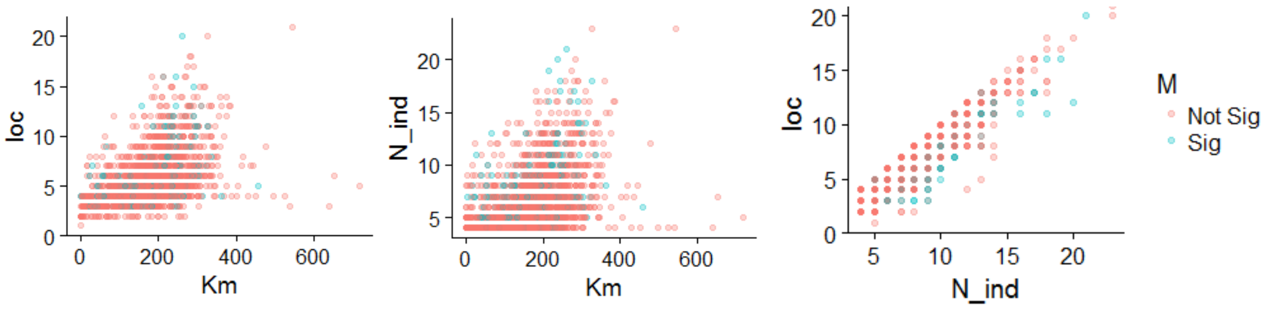

Supplement Figure 4. Dot plot showing the relationship between the sampling variables: number of localities (loc); number of individuals per species (N_ind); mean intraspecific geographic distance (km). Significant plots based on the Mantel test are indicated by color.

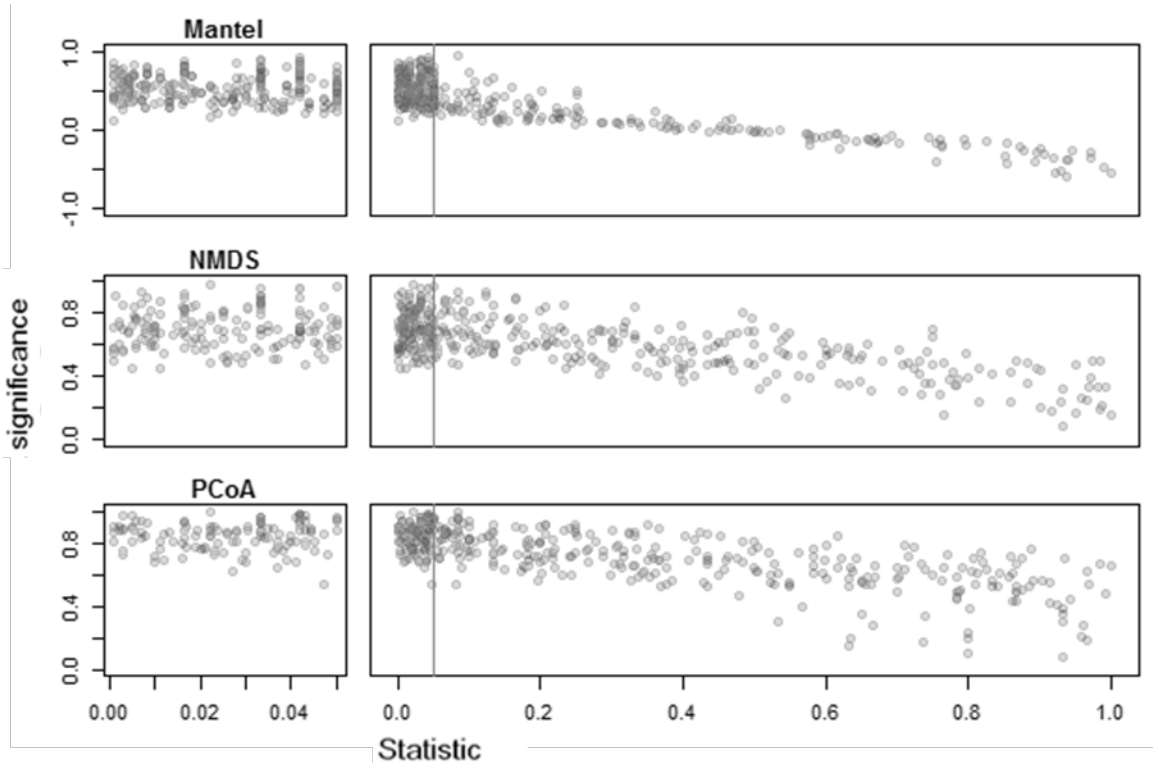

Supplement Figure 5. Significance levels for Mantel, NMDS, and PCA tests (y-axis: significance value; x-axis: p-value). On the left panel, significant species are shown in detail; right panel: all the examined species that showed a positive and significant relationship for at least one test.

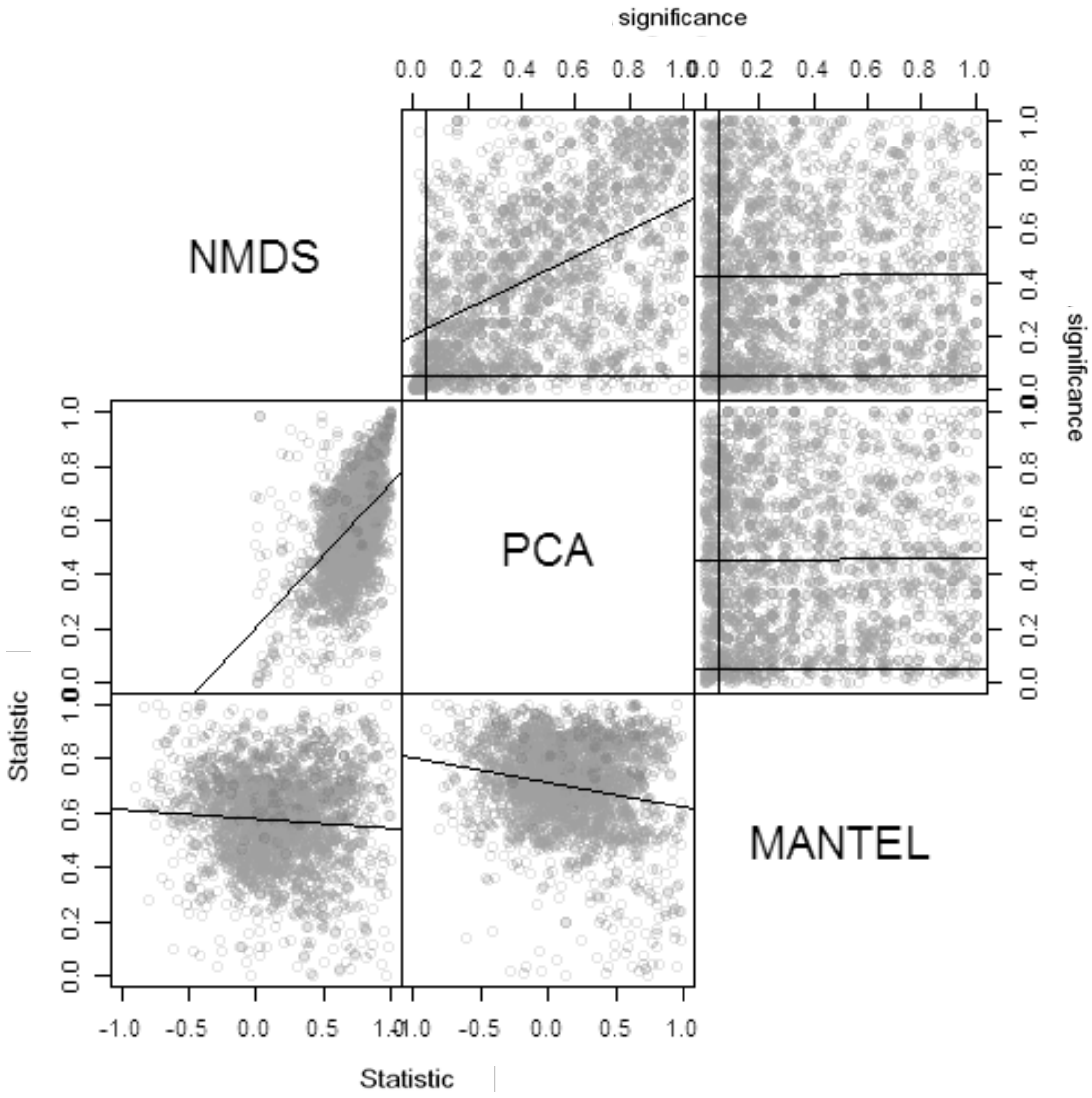

Supplement Figure 6. Comparison between the three correlation methods. on the bottom-left are regressed the three technique Statistic scores. On the top-left are regressed the significance scores.

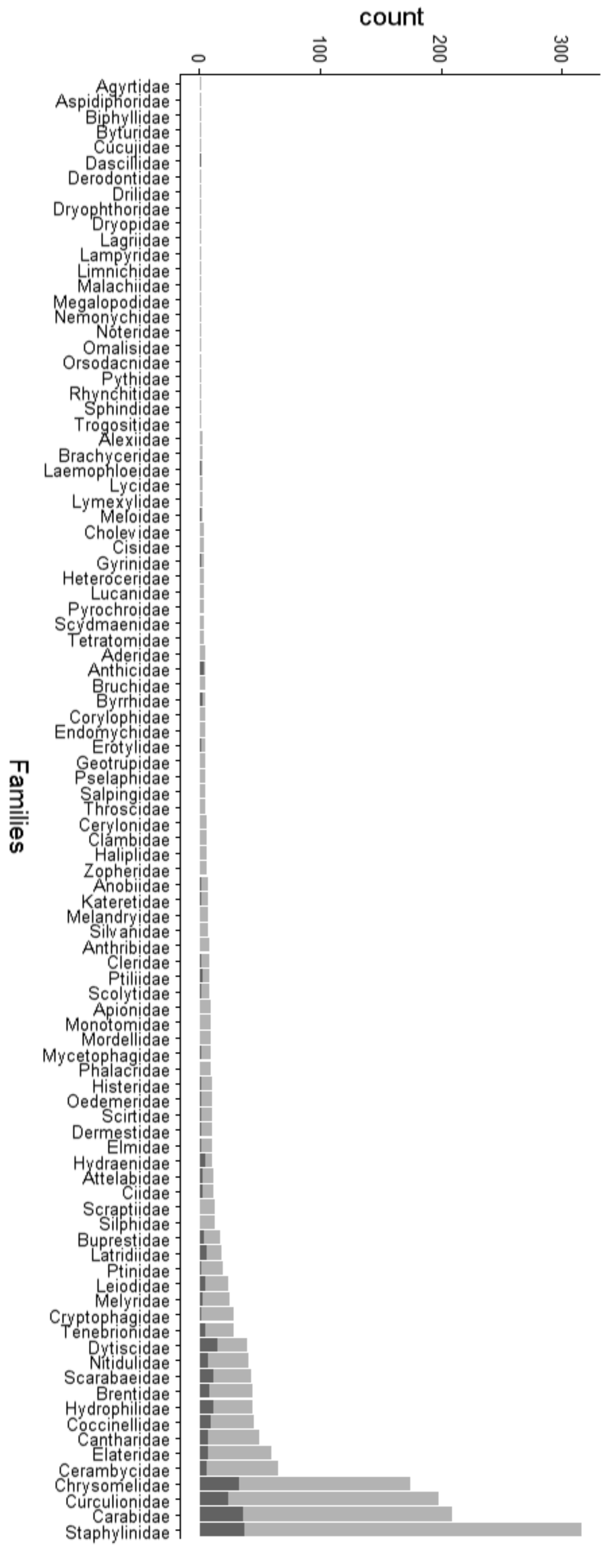

Supplement Figure 7. Histogram showing the distribution (species number/ =count) of the 1785 examined species across the 95 families. Species resulting significant in at least one of the three statistical tests performed in the study are indicated in dark grey, others are light grey.
