# Supplementary material for: From DNA barcodes to ecology: Meta‐analysis of central European beetles reveal link with species ecology but also to data pattern and gaps
Source: Ecol Evol. 2022 Dec 21;12(12):e9650. doi: 10.1002/ece3.9650 (PMC9771709; doi:10.1002/ece3.9650)
Supplement: Supplementary file 2 — Table S1 [file ECE3-12-e9650-s001.pdf]

Supplementary materials

S1

Tab. 1 metadata list inuclding all the examined species and theire relative ecological classes appartenence. The habitat abbreviations (hab) stand for: h_b =soil, h_e=eurytop, h_f= rotting matters, h_n=nest, h_p=vegetation, h_t=dead wood, h_v=vegetation; h_w= water; Biotope preference (Biot): b_e=no preference; b_f =wetlands ; b_o= open-land biotypes; b_w=forests. Body size classes (Size): s_xs= extra small; s_s= small; s_m=medium; s_l=large and s_xl= extra-large. Feeding style (Feed.Style): f_c = Coprophagous; f_e =Polyphagous; f_m= Mycetophagous; f_n= Necrophagous;f_p= Phytophagous; f_s= Saprophagous; f_x= Xylophagous; f_z= Zoophagous; Geographical distance classes: highest treashold measured in km.

Species name	Autor	Family	N.ind	Loc	Size	Biot	Hab	Feed	Dist
Abax ovalis	(Duftschmid, 1812)	Carabidae	7	7	s_l	b_w	h_b	f_z	300
Abax parallelepipedus	(Piller & Mitterpacher)	Carabidae	13	11	s_l	b_w	h_b	f_z	300
Abax parallelus	(Duftschmid, 1812)	Carabidae	6	6	s_l	b_w	h_b	f_z	200
Abdera quadrifasciata	(Curtis, 1829)	Melandryidae	5	4	s_s	b_w	h_t	f_x	100
Abraeus perpusillus	(Marsham, 1802)	Histeridae	5	5	s_xs	b_w	h_t	f_z	100
Acalles micros	(Dieckmann, 1982)	Curculionidae	4	3	s_xs	b_w	h_t	f_x	100
Acalles ptinoides	(Marsham, 1802)	Curculionidae	7	7	s_xs	b_w	h_t	f_x	150
Acanthocinus griseus	(Fabricius, 1792)	Cerambycidae	5	3	s_m	b_w	h_t	f_x	200
Acilius sulcatus	(Linné, 1758)	Dytiscidae	4	4	s_l	b_f	h_w	f_z	300
Acrotona parens	(Mulsant & Rey, 1852)	Staphylinidae	6	4	s_xs	b_e	h_b	f_z	100
Acrotona parvula	(Mannerheim, 1830)	Staphylinidae	8	6	s_xs	b_e	h_f	f_z	100
Acupalpus dubius	Schilsky, 1888	Carabidae	4	4	s_s	b_f	h_b	f_z	300
Acupalpus exiguus	(Dejean, 1829)	Carabidae	5	5	s_xs	b_f	h_b	f_z	150
Acupalpus luteatus	(Duftschmid, 1812)	Carabidae	5	3	s_s	b_f	h_b	f_z	100
Acupalpus meridianus	(Linné, 1761)	Carabidae	5	4	s_s	b_o	h_b	f_z	200
Acupalpus parvulus	(J. Sturm, 1825)	Carabidae	10	9	s_s	b_f	h_b	f_z	300
Acylophorus wagenschieberi	(Kiesenwetter, 1850)	Staphylinidae	5	3	s_m	b_f	h_b	f_z	100
Adalia bipunctata	(Linné, 1758)	Coccinellidae	9	8	s_s	b_e	h_v	f_z	300
Adalia decempunctata	(Linné, 1758)	Coccinellidae	4	4	s_s	b_e	h_v	f_z	150
Aderus populneus	(Creutzer, 1796)	Aderidae	12	8	s_xs	b_w	h_t	f_x	300
Adrastus axillaris	Erichson, 1841	Elateridae	6	6	s_s	b_f	h_v	f_e	200
Adrastus lacertosus	Erichson, 1841	Elateridae	13	9	s_s	b_f	h_v	f_e	100
Adrastus montanus	(Scopoli, 1763)	Elateridae	4	4	s_s	b_o	h_v	f_e	100
Adrastus pallens	(Fabricius, 1792)	Elateridae	12	11	s_s	b_e	h_v	f_e	300
Adrastus rachifer	(Geoffroy, 1785)	Elateridae	8	8	s_s	b_e	h_v	f_e	200
Agabus affinis	(Paykull, 1798)	Dytiscidae	6	5	s_m	b_f	h_w	f_z	300
Agabus congener	(Thunberg, 1794)	Dytiscidae	7	4	s_m	b_f	h_w	f_z	300
Agabus guttatus	(Paykull, 1798)	Dytiscidae	4	3	s_m	b_f	h_w	f_z	150
Agabus melanarius	Aubé, 1837	Dytiscidae	4	3	s_m	b_f	h_w	f_z	150
Agabus nebulosus	(Forster, 1771)	Dytiscidae	7	5	s_m	b_f	h_w	f_z	200
Agabus sturmii	(Gyllenhal, 1808)	Dytiscidae	4	4	s_m	b_f	h_w	f_z	200
Agabus uliginosus	(Linné, 1761)	Dytiscidae	10	6	s_m	b_f	h_w	f_z	150
Agabus undulatus	(Schrank, 1776)	Dytiscidae	8	6	s_m	b_f	h_w	f_z	300
Agapanthia villosoviridescens	(DeGeer, 1775)	Cerambycidae	9	9	s_l	b_o	h_v	f_p	>300

Agaricochara latissima	(Stephens, 1832)	Staphylinidae	6	4	s_xs	b_w	h_t	f_m	200
Agathidium atrum	(Paykull, 1798)	Leiodidae	5	5	s_s	b_w	h_p	f_m	200
Agathidium laevigatum	Erichson, 1845	Leiodidae	7	5	s_xs	b_e	h_p	f_m	200
Agathidium marginatum	J. Sturm, 1807	Leiodidae	4	4	s_xs	b_e	h_p	f_m	150
Agathidium nigrinum	J. Sturm, 1807	Leiodidae	5	3	s_s	b_w	h_p	f_m	150
Agathidium nigripenne	(Fabricius, 1792)	Leiodidae	5	5	s_s	b_w	h_t	f_m	200
Agathidium rotundatum	(Gyllenhal, 1827)	Leiodidae	5	4	s_xs	b_w	h_p	f_m	100
Agathidium seminulum	(Linné, 1758)	Leiodidae	8	8	s_xs	b_w	h_p	f_m	300
Agathidium varians	L. Beck, 1817	Leiodidae	4	4	s_xs	b_w	h_p	f_m	100
Agelastica alni	(Linné, 1758)	Chrysomelidae	7	7	s_m	b_w	h_v	f_p	300
Agonum duftschmidi	J. Schmidt, 1994	Carabidae	5	2	s_m	b_f	h_b	f_z	300
Agonum emarginatum	(Gyllenhal, 1827)	Carabidae	6	5	s_m	b_f	h_b	f_z	200
Agonum fuliginosum	(Panzer, 1809)	Carabidae	9	9	s_m	b_f	h_b	f_z	300
Agonum lugens	(Duftschmid, 1812)	Carabidae	6	6	s_m	b_f	h_b	f_z	300
Agonum marginatum	(Linné, 1758)	Carabidae	8	7	s_m	b_f	h_b	f_z	200
Agonum micans	(Nicolai, 1822)	Carabidae	12	11	s_m	b_f	h_b	f_z	200
Agonum muelleri	(Herbst, 1784)	Carabidae	9	8	s_m	b_e	h_b	f_z	300
Agonum piceum	(Linné, 1758)	Carabidae	5	2	s_m	b_f	h_b	f_z	100
Agonum thoreyi	Dejean, 1828	Carabidae	6	6	s_m	b_f	h_b	f_z	300
Agonum viduum	(Panzer, 1796)	Carabidae	6	5	s_m	b_f	h_b	f_z	>300
Agrilus biguttatus	(Fabricius, 1777)	Buprestidae	4	4	s_m	b_w	h_t	f_x	150
Agrilus convexicollis	L. Redtenbacher, 1849	Buprestidae	4	4	s_s	b_w	h_t	f_x	150
Agrilus integerrimus	(Ratzeburg, 1837)	Buprestidae	4	3	s_m	b_w	h_t	f_x	150
Agrilus sulcicollis	Lacordaire, 1835	Buprestidae	8	7	s_m	b_w	h_t	f_x	300
Agrilus viridis	(Linné, 1758)	Buprestidae	7	6	s_m	b_w	h_t	f_x	200
Agriotes acuminatus	(Stephens, 1830)	Elateridae	6	6	s_m	b_o	h_v	f_p	200
Agriotes lineatus	(Linné, 1767)	Elateridae	7	7	s_m	b_o	h_v	f_p	200
Agriotes obscurus	(Linné, 1758)	Elateridae	6	6	s_m	b_o	h_v	f_p	300
Agriotes pilosellus	(Schönherr, 1817)	Elateridae	7	7	s_l	b_w	h_v	f_p	200
Agriotes sputator	(Linné, 1758)	Elateridae	6	6	s_m	b_o	h_v	f_p	200
Agriotes ustulatus	(Schaller, 1783)	Elateridae	12	11	s_m	b_o	h_v	f_p	>300
Agrypnus murinus	(Linné, 1758)	Elateridae	7	7	s_l	b_o	h_v	f_z	300
Ahasverus advena	(Waltl, 1834)	Silvanidae	4	4	s_xs	b_e	h_f	f_m	100
Aizobius sedi	(Germar, 1818)	Brentidae	5	3	s_xs	b_o	h_v	f_p	100
Aleochara curtula	(Goeze, 1777)	Staphylinidae	6	6	s_m	b_e	h_f	f_z	200
Aleochara haemoptera	Kraatz, 1856	Staphylinidae	5	4	s_s	b_o	h_n	f_z	150
Aleochara intricata	Mannerheim, 1830	Staphylinidae	7	5	s_s	b_o	h_f	f_z	100
Aleochara sparsa	Heer, 1839	Staphylinidae	5	4	s_s	b_w	h_f	f_z	200
Aloconota cambrica	(Wollaston, 1855)	Staphylinidae	4	4	s_xs	b_f	h_b	f_z	300
Alosterna tabacicolor	(DeGeer, 1775)	Cerambycidae	14	13	s_m	b_w	h_t	f_x	>300
Alphitophagus bifasciatus	(Say, 1823)	Tenebrionidae	5	4	s_xs	b_e	h_f	f_s	200
Altica aenescens	J. Weise, 1888	Chrysomelidae	8	4	s_s	b_o	h_v	f_p	200
Altica oleracea	(Linné, 1758)	Chrysomelidae	5	4	s_s	b_o	h_v	f_p	150
Amalus scortillum	(Herbst, 1795)	Curculionidae	4	4	s_xs	b_o	h_v	f_p	200
Amara aenea	(DeGeer, 1774)	Carabidae	7	7	s_m	b_e	h_b	f_p	>300
Amara apricaria	(Paykull, 1790)	Carabidae	8	7	s_m	b_o	h_b	f_p	150
Amara aulica	(Panzer, 1796)	Carabidae	5	4	s_l	b_o	h_v	f_p	200

Amara bifrons	(Gyllenhal, 1810)	Carabidae	8	8	s_m	b_o	h_b	f_p	200
Amara convexior	Stephens, 1828	Carabidae	5	4	s_m	b_e	h_b	f_p	200
Amara convexiuscula	(Marsham, 1802)	Carabidae	4	4	s_l	b_o	h_b	f_p	200
Amara erratica	(Duftschmid, 1812)	Carabidae	7	6	s_m	b_o	h_b	f_p	100
Amara familiaris	(Duftschmid, 1812)	Carabidae	4	4	s_m	b_e	h_b	f_p	300
Amara fulva	(O. F. Müller, 1776)	Carabidae	4	4	s_m	b_o	h_b	f_p	300
Amara lunicollis	Schiødte, 1837	Carabidae	4	4	s_m	b_o	h_b	f_p	300
Amara majuscula	Chaudoir, 1850	Carabidae	4	4	s_m	b_o	h_b	f_p	200
Amara quenseli	(C. Zimmermann, 1832)	Carabidae	8	7	s_m	b_o	h_b	f_p	300
Amischa analis	(Gravenhorst, 1802)	Staphylinidae	6	6	s_xs	b_e	h_b	f_z	200
Amischa decipiens	(Sharp, 1869)	Staphylinidae	4	4	s_xs	b_f	h_b	f_z	150
Ampedus aethiops	(Lacordaire, 1835)	Elateridae	9	6	s_m	b_w	h_t	f_x	300
Ampedus balteatus	(Linné, 1758)	Elateridae	18	18	s_m	b_w	h_t	f_x	300
Ampedus elongatulus	(Fabricus, 1787)	Elateridae	11	10	s_m	b_w	h_t	f_x	200
Ampedus nigrinus	(Herbst, 1784)	Elateridae	5	5	s_m	b_w	h_t	f_x	200
Ampedus pomonae	(Stephens, 1830)	Elateridae	8	4	s_m	b_w	h_t	f_x	200
Ampedus pomorum	(Herbst, 1784)	Elateridae	14	13	s_m	b_w	h_t	f_x	300
Ampedus quercicola	(Buysson, 1887)	Elateridae	9	8	s_m	b_w	h_t	f_x	100
Ampedus rufipennis	(Stephens, 1830)	Elateridae	6	4	s_l	b_w	h_t	f_x	100
Ampedus sanguineus	(Linné, 1758)	Elateridae	7	7	s_l	b_w	h_t	f_x	300
Ampedus sanguinolentus	(Schrank, 1776)	Elateridae	4	4	s_m	b_w	h_t	f_x	300
Amphichroum canaliculatum	(Erichson, 1840)	Staphylinidae	8	3	s_s	b_w	h_v	f_z	100
Amphicyllis globus	(Fabricius, 1792)	Leiodidae	5	5	s_xs	b_w	h_p	f_m	300
Amphimallon solstitiale	(Linné, 1758)	Scarabaeidae	11	9	s_l	b_o	h_v	f_p	>300
Amphotis marginata	(Fabricius, 1781)	Nitidulidae	8	7	s_s	b_w	h_t	f_s	200
Anacaena globulus	(Paykull, 1798)	Hydrophilidae	16	13	s_s	b_f	h_w	f_s	>300
Anacaena limbata	(Fabricius, 1792)	Hydrophilidae	8	7	s_xs	b_f	h_w	f_s	300
Anacaena lutescens	(Stephens, 1829)	Hydrophilidae	6	6	s_xs	b_f	h_w	f_s	>300
Anaesthetis testacea	(Fabricius, 1781)	Cerambycidae	5	4	s_m	b_w	h_t	f_x	150
Anaglyptus mysticus	(Linné, 1758)	Cerambycidae	5	5	s_m	b_w	h_t	f_x	150
Anaspis fasciata	(Forster, 1771)	Scraptiidae	5	5	s_xs	b_w	h_t	f_x	100
Anaspis flava	(Linné, 1758)	Scraptiidae	6	6	s_s	b_w	h_t	f_x	200
Anaspis frontalis	(Linné, 1758)	Scraptiidae	10	10	s_s	b_w	h_t	f_x	300
Anaspis kiesenwetteri	Emery, 1876	Scraptiidae	5	4	s_s	b_o	h_t	f_x	100
Anaspis latiuscula	(Mulsant, 1856)	Scraptiidae	4	2	s_xs	b_w	h_t	f_x	100
Anaspis maculata	(Geoffroy, 1785)	Scraptiidae	5	5	s_xs	b_w	h_t	f_x	300
Anaspis pulicaria	A. Costa, 1854	Scraptiidae	7	5	s_xs	b_w	h_t	f_x	200
Anaspis ruficollis	(Fabricius, 1792)	Scraptiidae	7	7	s_xs	b_w	h_t	f_x	300
Anaspis rufilabris	(Gyllenhal, 1827)	Scraptiidae	7	6	s_xs	b_w	h_t	f_x	200
Anaspis thoracica	(Linné, 1758)	Scraptiidae	6	5	s_xs	b_w	h_t	f_x	300
Anaspis varians	(Mulsant, 1856)	Scraptiidae	5	5	s_xs	b_o	h_t	f_x	100
Anastrangalia dubia	(Scopoli, 1763)	Cerambycidae	10	8	s_l	b_w	h_t	f_x	150
Anastrangalia reyi	(Heyden, 1889)	Cerambycidae	8	6	s_m	b_w	h_t	f_x	100
Anastrangalia sanguinolenta	(Linné, 1760)	Cerambycidae	16	13	s_m	b_w	h_t	f_x	>300
Anatis ocellata	(Linné, 1758)	Coccinellidae	4	4	s_m	b_w	h_v	f_z	100
Anchomenus dorsalis	(Pontoppidan, 1763)	Carabidae	7	7	s_m	b_o	h_b	f_z	300
Ancistronycha abdominalis	(Fabricius, 1798)	Cantharidae	5	5	s_l	b_w	h_v	f_z	150

Ancistronycha tigurina	Dietrich, 1857	Cantharidae	7	6	s_l	b_w	h_v	f_z	200
Andrion regensteinense	(Herbst, 1797)	Curculionidae	5	4	s_s	b_o	h_v	f_p	100
Anidorus nigrinus	(Germar, 1842)	Aderidae	5	3	s_xs	b_w	h_t	f_x	100
Anisandrus dispar	(Fabricius, 1792)	Curculionidae	5	4	s_xs	b_w	h_t	f_m	150
Anisodactylus binotatus	(Fabricius, 1787)	Carabidae	9	9	s_l	b_o	h_b	f_p	300
Anisodactylus poeciloides	(Stephens, 1828)	Carabidae	5	3	s_l	b_f	h_b	f_p	100
Anisosticta novemdecimpunctata	(Linné, 1758)	Coccinellidae	6	6	s_s	b_f	h_v	f_z	300
Anisotoma castanea	(Herbst, 1792)	Leiodidae	7	6	s_s	b_w	h_t	f_m	200
Anisotoma humeralis	(Fabricius, 1792)	Leiodidae	11	11	s_s	b_w	h_t	f_m	300
Anisoxya fuscula	(Illiger, 1798)	Melandryidae	7	7	s_s	b_w	h_t	f_x	200
Anogcodes rufiventris	(Scopoli, 1763)	Oedemeridae	7	6	s_m	b_o	h_t	f_x	100
Anomala dubia	(Scopoli, 1763)	Scarabaeidae	5	5	s_l	b_o	h_v	f_p	300
Anomognathus cuspidatus	(Erichson, 1839)	Staphylinidae	6	5	s_xs	b_w	h_t	f_z	300
Anoplodera sexguttata	(Fabricius, 1775)	Cerambycidae	11	9	s_m	b_w	h_t	f_x	100
Anoplotrupes stercorosus	(Scriba, 1791)	Geotrupidae	7	6	s_l	b_w	h_f	f_s	300
Anotylus clypeonitens	(Pandellé, 1867)	Staphylinidae	4	4	s_xs	b_o	h_f	f_s	150
Anotylus insecatus	(Gravenhorst, 1806)	Staphylinidae	5	5	s_s	b_o	h_f	f_s	300
Anotylus inustus	(Gravenhorst, 1806)	Staphylinidae	5	5	s_s	b_o	h_f	f_s	100
Anotylus mutator	(Lohse, 1963)	Staphylinidae	8	7	s_s	b_w	h_f	f_s	150
Anotylus nitidulus	(Gravenhorst, 1802)	Staphylinidae	8	7	s_xs	b_f	h_f	f_s	100
Anotylus rugosus	(Fabricius, 1775)	Staphylinidae	7	7	s_s	b_e	h_f	f_s	300
Anotylus sculpturatus	(Gravenhorst, 1806)	Staphylinidae	5	4	s_s	b_e	h_f	f_s	150
Anotylus tetracarinatus	(Block, 1799)	Staphylinidae	6	5	s_xs	b_e	h_f	f_s	150
Anthaxia godeti	Gory & Laporte, 1839	Buprestidae	4	4	s_s	b_w	h_t	f_x	200
Anthaxia helvetica	Stierlin, 1868	Buprestidae	11	11	s_m	b_w	h_t	f_x	300
Anthaxia morio	(Fabricius, 1792)	Buprestidae	4	4	s_m	b_w	h_t	f_x	100
Anthaxia nitidula	(Linné, 1758)	Buprestidae	8	7	s_m	b_o	h_t	f_x	150
Anthaxia quadripunctata	(Linné, 1758)	Buprestidae	12	11	s_m	b_w	h_t	f_x	300
Antherophagus pallens	(Linné, 1758)	Cryptophagidae	10	9	s_s	b_e	h_n	f_m	300
Anthicus flavipes	(Panzer, 1796)	Anthicidae	10	7	s_xs	b_o	h_b	f_z	300
Anthobium atrocephalum	(Gyllenhal, 1827)	Staphylinidae	13	12	s_s	b_w	h_b	f_s	300
Anthobium unicolor	(Marsham, 1802)	Staphylinidae	4	4	s_s	b_w	h_b	f_s	150
Anthocomus fasciatus	(Linné, 1758)	Melyridae	11	10	s_s	b_e	h_v	f_m	200
Anthocomus rufus	(Herbst, 1784)	Melyridae	8	6	s_s	b_f	h_v	f_m	>300
Anthonomus pedicularius	(Linné, 1758)	Curculionidae	13	12	s_s	b_w	h_v	f_p	200
Anthonomus phyllocola	(Herbst, 1795)	Curculionidae	8	7	s_xs	b_w	h_v	f_p	200
Anthonomus pomorum	(Linné, 1758)	Curculionidae	5	5	s_s	b_w	h_v	f_p	200
Anthonomus rectirostris	(Linné, 1758)	Curculionidae	5	4	s_s	b_w	h_v	f_p	200
Anthonomus rubi	(Herbst, 1795)	Curculionidae	11	11	s_xs	b_e	h_v	f_p	300
Anthophagus alpestris	Heer, 1839	Staphylinidae	11	8	s_s	b_e	h_v	f_z	100
Anthophagus alpinus	(Paykull, 1790)	Staphylinidae	9	7	s_s	b_f	h_v	f_z	100
Anthophagus bicornis	(Block, 1799)	Staphylinidae	9	9	s_s	b_e	h_v	f_z	300
Anthophagus fallax	Kiesenwetter, 1848	Staphylinidae	5	4	s_s	b_w	h_v	f_z	100
Anthophagus omalinus	Koch, 1933	Staphylinidae	6	4	s_s	b_f	h_v	f_z	100
Anthracus consputus	(Duftschmid, 1812)	Carabidae	8	8	s_s	b_w	h_b	f_z	200
Anthrenus museorum	(Linné, 1761)	Dermestidae	9	8	s_xs	b_o	h_f	f_n	300

Anthrenus pimpinellae	Fabricius, 1775	Dermestidae	4	4	s_s	b_e	h_n	f_n	200
Anthrenus scrophulariae	(Linné, 1758)	Dermestidae	4	4	s_xs	b_o	h_f	f_n	300
Anthribus nebulosus	Forster, 1770	Anthribidae	9	9	s_xs	b_w	h_v	f_z	300
Aphidecta obliterata	(Linné, 1758)	Coccinellidae	11	10	s_s	b_w	h_v	f_z	300
Aphodius abdominalis	Bonelli, 1812	Scarabaeidae	8	5	s_s	b_o	h_b	f_p	100
Aphodius ater	(DeGeer, 1774)	Scarabaeidae	10	7	s_s	b_e	h_f	f_c	150
Aphodius contaminatus	(Herbst, 1783)	Scarabaeidae	6	5	s_s	b_e	h_f	f_c	300
Aphodius depressus	(Kugelann, 1792)	Scarabaeidae	11	9	s_m	b_e	h_f	f_c	300
Aphodius distinctus	(O. F. Müller, 1776)	Scarabaeidae	5	4	s_s	b_o	h_f	f_c	200
Aphodius erraticus	(Linné, 1758)	Scarabaeidae	4	4	s_m	b_o	h_f	f_c	150
Aphodius fimetarius	(Linné, 1758)	Scarabaeidae	6	5	s_m	b_e	h_f	f_c	150
Aphodius fossor	(Linné, 1758)	Scarabaeidae	10	7	s_l	b_o	h_f	f_c	>300
Aphodius granarius	(Linné, 1767)	Scarabaeidae	5	5	s_s	b_e	h_f	f_s	200
Aphodius haemorrhoidalis	(Linné, 1758)	Scarabaeidae	8	6	s_s	b_o	h_f	f_c	150
Aphodius obscurus	(Fabricius, 1792)	Scarabaeidae	5	4	s_m	b_o	h_f	f_c	100
Aphodius prodromus	(Brahm, 1790)	Scarabaeidae	6	5	s_s	b_e	h_f	f_c	300
Aphodius pusillus	(Herbst, 1789)	Scarabaeidae	7	7	s_s	b_o	h_f	f_c	150
Aphodius rufipes	(Linné, 1758)	Scarabaeidae	6	6	s_l	b_e	h_f	f_c	200
Aphodius rufus	(Moll, 1782)	Scarabaeidae	7	6	s_m	b_o	h_f	f_c	200
Aphodius sphacelatus	(Panzer, 1798)	Scarabaeidae	10	5	s_s	b_e	h_f	f_c	200
Aphodius sticticus	(Panzer, 1798)	Scarabaeidae	4	3	s_s	b_e	h_f	f_c	100
Aphthona cyparissiae	(Koch, 1803)	Chrysomelidae	8	7	s_s	b_o	h_v	f_p	300
Aphthona herbigrada	(Curtis, 1837)	Chrysomelidae	9	8	s_xs	b_o	h_v	f_p	200
Aphthona nonstriata	(Goeze, 1777)	Chrysomelidae	12	8	s_xs	b_f	h_v	f_p	200
Aphthona venustula	(Kutschera, 1861)	Chrysomelidae	11	9	s_xs	b_o	h_v	f_p	200
Apion haematodes	Kirby, 1808	Brentidae	5	3	s_xs	b_o	h_v	f_p	100
Apion rubiginosum	Grill, 1893	Brentidae	5	3	s_xs	b_o	h_v	f_p	100
Aplocnemus impressus	(Marsham, 1802)	Melyridae	4	4	s_s	b_w	h_t	f_z	150
Aplocnemus virens	(SuffYian, 1843)	Melyridae	4	3	s_s	b_o	h_t	f_z	100
Aplotarsus incanus	(Gyllenhal, 1827)	Elateridae	8	7	s_m	b_o	h_v	f_e	300
Apoderus coryli	(Linné, 1758)	Attelabidae	4	3	s_m	b_w	h_v	f_p	150
Apteropeda globosa	(Illiger, 1794)	Chrysomelidae	4	3	s_xs	b_w	h_v	f_p	150
Apteropeda orbiculata	(Marsham, 1802)	Chrysomelidae	4	4	s_xs	b_w	h_v	f_p	150
Archarius pyrrhoceras	(Marsham, 1802)	Curculionidae	6	5	s_xs	b_e	h_v	f_p	150
Archarius salicivorus	(Paykull, 1792)	Curculionidae	4	4	s_xs	b_e	h_v	f_p	300
Arhopalus rusticus	(Linné, 1758)	Cerambycidae	17	16	s_l	b_w	h_t	f_x	300
Aromia moschata	(Linné, 1758)	Cerambycidae	6	6	s_xl	b_w	h_t	f_x	>300
Asaphidion flavipes	(Linné, 1761)	Carabidae	5	4	s_s	b_o	h_b	f_z	300
Aspidapion aeneum	(Fabricius, 1775)	Brentidae	5	4	s_s	b_o	h_v	f_p	200
Aspidapion radiolus	(Marsham, 1802)	Brentidae	5	5	s_xs	b_o	h_v	f_p	100
Aspidiphorus orbiculatus	(Gyllenhal, 1808)	Aspidiphoridae	8	7	s_xs	b_w	h_t	f_m	300
Astenus gracilis	(Paykull, 1789)	Staphylinidae	7	5	s_s	b_o	h_b	f_z	200
Atheta aegra	(Heer, 1841)	Staphylinidae	4	3	s_xs	b_e	h_f	f_z	100
Atheta aeneicollis	(Sharp, 1869)	Staphylinidae	4	4	s_s	b_w	h_f	f_z	150
Atheta cinnamoptera	(C. G. Thomson, 1856)	Staphylinidae	6	5	s_xs	b_w	h_f	f_z	300
Atheta corvina	(C. G. Thomson, 1856)	Staphylinidae	4	4	s_xs	b_w	h_p	f_z	150
Atheta crassicornis	(Fabricius, 1792)	Staphylinidae	4	4	s_s	b_w	h_p	f_z	100

Atheta elongatula	(Gravenhorst, 1802)	Staphylinidae	5	4	s_s	b_f	h_b	f_z	150
Atheta hygrotopora	(Kraatz, 1856)	Staphylinidae	8	7	s_s	b_f	h_b	f_z	200
Atheta incognita	(Sharp, 1869)	Staphylinidae	5	4	s_s	b_w	h_b	f_z	200
Atheta laticollis	(Stephens, 1832)	Staphylinidae	7	7	s_xs	b_e	h_f	f_z	200
Atheta marcida	(Erichson, 1837)	Staphylinidae	4	4	s_s	b_w	h_p	f_z	200
Atheta oblita	(Erichson, 1839)	Staphylinidae	4	3	s_xs	b_w	h_t	f_z	150
Atheta orbata	(Erichson, 1837)	Staphylinidae	4	4	s_xs	b_o	h_b	f_z	100
Atheta pallidicornis	(C. G. Thomson, 1856)	Staphylinidae	4	3	s_xs	b_w	h_p	f_z	300
Atheta palustris	(Kiesenwetter, 1844)	Staphylinidae	10	7	s_xs	b_f	h_b	f_z	300
Atheta picipes	(C. G. Thomson, 1856)	Staphylinidae	6	3	s_xs	b_w	h_t	f_z	200
Atheta pittionii	Scheerpeltz, 1950	Staphylinidae	4	2	s_xs	b_e	h_f	f_z	100
Atheta ravilla	(Erichson, 1839)	Staphylinidae	4	4	s_xs	b_e	h_f	f_z	150
Atheta sodalis	(Erichson, 1837)	Staphylinidae	6	6	s_xs	b_w	h_f	f_z	300
Atheta testaceipes	(Heer, 1839)	Staphylinidae	4	3	s_xs	b_e	h_f	f_z	100
Atheta tibialis	(Heer, 1839)	Staphylinidae	7	2	s_xs	b_w	h_b	f_z	100
Atheta triangulum	(Kraatz, 1856)	Staphylinidae	4	3	s_s	b_e	h_f	f_z	200
Atheta vaga	(Heer, 1839)	Staphylinidae	7	6	s_xs	b_e	h_f	f_z	150
Athous bicolor	(Goeze, 1777)	Elateridae	5	5	s_m	b_o	h_v	f_p	300
Athous haemorrhoidalis	(Fabricius, 1801)	Elateridae	4	4	s_l	b_e	h_v	f_p	100
Athous subfuscus	(O. F. Müller, 1764)	Elateridae	6	6	s_m	b_w	h_v	f_p	300
Athous vittatus	(Fabricius, 1792)	Elateridae	4	4	s_m	b_w	h_v	f_p	300
Atomaria analis	Erichson, 1846	Cryptophagidae	4	3	s_xs	b_e	h_e	f_m	100
Atomaria basalis	Erichson, 1846	Cryptophagidae	5	3	s_xs	b_f	h_b	f_m	150
Atomaria fuscata	(Schönherr, 1808)	Cryptophagidae	7	7	s_xs	b_e	h_e	f_m	200
Atomaria gutta	Newman, 1834	Cryptophagidae	4	4	s_xs	b_f	h_f	f_m	150
Atomaria lewisi	Reitter, 1877	Cryptophagidae	5	5	s_xs	b_e	h_f	f_m	100
Atomaria linearis	Stephens, 1830	Cryptophagidae	9	8	s_xs	b_e	h_f	f_m	200
Atomaria mesomela	(Herbst, 1792)	Cryptophagidae	4	3	s_xs	b_f	h_b	f_m	100
Atomaria nigrirostris	Stephens, 1830	Cryptophagidae	6	5	s_xs	b_e	h_b	f_m	200
Atomaria ornata	Heer, 1841	Cryptophagidae	5	2	s_xs	b_w	h_t	f_m	100
Atomaria pulchra	Erichson, 1846	Cryptophagidae	5	3	s_xs	b_w	h_t	f_m	100
Atomaria turgida	Erichson, 1846	Cryptophagidae	6	5	s_xs	b_w	h_t	f_m	300
Atrecus affinis	(Paykull, 1789)	Staphylinidae	12	12	s_m	b_w	h_t	f_z	300
Attagenus pellio	(Linné, 1758)	Dermestidae	5	5	s_s	b_e	h_f	f_n	200
Attagenus unicolor	(Brahm, 1790)	Dermestidae	4	4	s_s	b_e	h_f	f_n	150
Attelabus nitens	(Scopoli, 1763)	Attelabidae	9	9	s_s	b_w	h_v	f_p	200
Augyles hispidulus	(Kiesenwetter, 1843)	Heteroceridae	5	4	s_s	b_f	h_b	f_p	150
Aulonothroscus brevicollis	(Bonvouloir, 1859)	Throscidae	5	4	s_xs	b_w	h_b	f_m	300
Autalia longicornis	Scheerpeltz, 1947	Staphylinidae	5	5	s_xs	b_w	h_p	f_z	200
Autalia rivularis	(Gravenhorst, 1802)	Staphylinidae	7	7	s_xs	b_e	h_f	f_z	200
Axinotarsus marginalis	(Laporte, 1840)	Melyridae	8	8	s_xs	b_w	h_v	f_z	150
Axinotarsus pulicarius	(Fabricius, 1777)	Melyridae	9	8	s_s	b_o	h_v	f_z	150
Axinotarsus ruficollis	(A. G. Olivier, 1790)	Melyridae	9	7	s_xs	b_w	h_v	f_z	300
Badister bullatus	(Schrank, 1798)	Carabidae	14	13	s_s	b_e	h_b	f_z	300
Badister collaris	Motschulsky, 1844	Carabidae	9	7	s_s	b_f	h_b	f_z	300
Badister dilatatus	Chaudoir, 1837	Carabidae	4	3	s_s	b_w	h_b	f_z	200
Badister sodalis	(Duftschmid, 1812)	Carabidae	5	5	s_s	b_f	h_b	f_z	300

Baeocrara variolosa	(Mulsant & Rey, 1861)	Ptiliidae	7	6	s_xs	b_w	h_t	f_m	300
Baris artemisiae	(Herbst, 1794)	Curculionidae	5	4	s_s	b_o	h_v	f_p	300
Batophila rubi	(Paykull, 1799)	Chrysomelidae	14	13	s_xs	b_e	h_v	f_p	>300
Batrisodes delaporti	(Aubé, 1833)	Staphylinidae	5	5	s_xs	b_w	h_t	f_z	150
Bembidion articulatum	(Panzer, 1796)	Carabidae	7	7	s_s	b_f	h_b	f_z	300
Bembidion aspericolle	(Germar, 1829)	Carabidae	7	6	s_xs	b_f	h_b	f_z	100
Bembidion assimile	(Gyllenhal, 1810)	Carabidae	18	13	s_s	b_f	h_b	f_z	300
Bembidion atrocaeruleum	(Stephens, 1828)	Carabidae	4	3	s_s	b_f	h_b	f_z	150
Bembidion azurescens	(Dalla Torre, 1877)	Carabidae	7	6	s_xs	b_f	h_b	f_z	300
Bembidion biguttatum	(Fabricius, 1779)	Carabidae	10	9	s_s	b_f	h_b	f_z	>300
Bembidion bipunctatum	(Heer, 1837)	Carabidae	6	5	s_s	b_f	h_b	f_z	100
Bembidion complanatum	Heer, 1837	Carabidae	4	4	s_s	b_f	h_b	f_z	150
Bembidion conforme	(Dejean, 1831)	Carabidae	4	2	s_s	b_f	h_b	f_z	100
Bembidion cruciatum	Jacquelin du Val, 1852	Carabidae	4	3	s_s	b_f	h_b	f_z	300
Bembidion decorum	(Panzer, 1799)	Carabidae	8	7	s_s	b_f	h_b	f_z	200
Bembidion deletum	Audinet-Serville, 1821	Carabidae	5	5	s_s	b_f	h_b	f_z	300
Bembidion dentellum	(Thunberg, 1787)	Carabidae	12	11	s_s	b_f	h_b	f_z	>300
Bembidion femoratum	J. Sturm, 1825	Carabidae	6	5	s_s	b_o	h_b	f_z	300
Bembidion fluviatile	(Dejean, 1831)	Carabidae	8	3	s_s	b_f	h_b	f_z	150
Bembidion gilvipes	(J. Sturm, 1825)	Carabidae	6	3	s_xs	b_f	h_b	f_z	150
Bembidion glaciale	(Heer, 1837)	Carabidae	4	2	s_s	b_f	h_b	f_z	100
Bembidion guttula	(Fabricius, 1792)	Carabidae	9	8	s_s	b_f	h_b	f_z	200
Bembidion illigeri	(Netolitzky, 1914)	Carabidae	7	5	s_s	b_f	h_b	f_z	300
Bembidion incognitum	J. [G.] Müller, 1931	Carabidae	4	2	s_s	b_f	h_b	f_z	100
Bembidion lampros	(Herbst, 1784)	Carabidae	9	8	s_s	b_e	h_b	f_z	300
Bembidion litorale	(A. G. Olivier, 1790)	Carabidae	8	4	s_s	b_f	h_b	f_z	100
Bembidion lunulatum	(Geoffroy, 1785)	Carabidae	6	6	s_s	b_f	h_b	f_z	300
Bembidion mannerheimii	(C. R. Sahlberg, 1827)	Carabidae	6	6	s_s	b_f	h_b	f_z	300
Bembidion modestum	(Fabricius, 1801)	Carabidae	4	3	s_s	b_f	h_b	f_z	150
Bembidion monticola	J. Sturm, 1825	Carabidae	5	3	s_s	b_f	h_b	f_z	100
Bembidion obliquum	J. Sturm, 1825	Carabidae	5	5	s_s	b_f	h_b	f_z	100
Bembidion obtusum	(Audinet-Serville, 1821)	Carabidae	11	9	s_s	b_o	h_b	f_z	150
Bembidion octomaculatum	(Goeze, 1777)	Carabidae	13	12	s_xs	b_f	h_b	f_z	200
Bembidion properans	(Stephens, 1828)	Carabidae	10	7	s_s	b_f	h_b	f_z	300
Bembidion punctulatum	Drapiez, 1820	Carabidae	10	10	s_s	b_f	h_b	f_z	>300
Bembidion pygmaeum	(Fabricius, 1792)	Carabidae	4	3	s_s	b_f	h_b	f_z	150
Bembidion quadrimaculatum	(Linné, 1761)	Carabidae	16	15	s_s	b_e	h_b	f_z	>300
Bembidion saxatile	Gyllenhal, 1827	Carabidae	4	3	s_s	b_f	h_b	f_z	300
Bembidion schueppelii	(Dejean, 1831)	Carabidae	5	3	s_s	b_f	h_b	f_z	200
Bembidion semipunctatum	(Donovan, 1806)	Carabidae	6	6	s_s	b_f	h_b	f_z	300
Bembidion stephensii	Crotch, 1866	Carabidae	4	3	s_s	b_f	h_b	f_z	200
Bembidion stomoides	(Dejean, 1831)	Carabidae	6	4	s_s	b_f	h_b	f_z	300
Bembidion tenellum	(Erichson, 1837)	Carabidae	7	4	s_xs	b_f	h_b	f_z	300
Bembidion testaceum	(Duftschmid, 1812)	Carabidae	7	7	s_s	b_f	h_b	f_z	300
Bembidion tetracolum	Say, 1823	Carabidae	13	12	s_s	b_e	h_b	f_z	>300
Bembidion tibiale	(Duftschmid, 1812)	Carabidae	13	11	s_m	b_f	h_b	f_z	>300
Bembidion varium	(A. G. Olivier, 1795)	Carabidae	11	7	s_s	b_f	h_b	f_z	300

Bembidion velox	(Linné, 1761)	Carabidae	7	4	s_s	b_f	h_b	f_z	150
Berosus luridus	(Linné, 1760)	Hydrophilidae	6	3	s_s	b_f	h_w	f_s	150
Betulapion simile	(Kirby, 1811)	Brentidae	13	12	s_xs	b_e	h_v	f_p	300
Bibloplectus pusillus	(Denny, 1825)	Staphylinidae	5	4	s_xs	b_f	h_b	f_z	100
Bibloporus bicolor	(Denny, 1825)	Staphylinidae	9	8	s_xs	b_w	h_t	f_z	200
Bisnius fimetarius	(Gravenhorst, 1802)	Staphylinidae	11	9	s_m	b_e	h_f	f_z	300
Bisnius sordidus	(Gravenhorst, 1802)	Staphylinidae	4	4	s_m	b_e	h_f	f_z	150
Bisnius subuliformis	(Gravenhorst, 1802)	Staphylinidae	7	7	s_m	b_w	h_t	f_z	300
Bitoma crenata	(Fabricius, 1775)	Zopheridae	17	15	s_s	b_w	h_t	f_z	300
Bledius erraticus	Erichson, 1839	Staphylinidae	8	7	s_s	b_o	h_b	f_p	300
Bledius gallicus	(Gravenhorst, 1806)	Staphylinidae	8	8	s_s	b_f	h_b	f_p	300
Bledius opacus	(Block, 1799)	Staphylinidae	11	9	s_s	b_f	h_b	f_p	300
Bledius tricornis	(Herbst, 1784)	Staphylinidae	12	9	s_m	b_f	h_b	f_p	300
Blethisa multipunctata	(Linné, 1758)	Carabidae	5	4	s_l	b_f	h_b	f_z	200
Bolitochara bella	Märkel, 1844	Staphylinidae	4	4	s_s	b_w	h_t	f_z	150
Bolitochara mulsanti	Sharp, 1875	Staphylinidae	9	8	s_s	b_w	h_t	f_z	300
Bolitochara obliqua	Erichson, 1837	Staphylinidae	11	11	s_s	b_w	h_t	f_z	300
Bolitochara tecta	Gravenhorst, 1802	Staphylinidae	4	4	s_s	b_w	h_t	f_z	150
Bolitophagus reticulatus	(Linné, 1767)	Tenebrionidae	16	15	s_m	b_w	h_t	f_m	300
Brachida exigua	(Heer, 1839)	Staphylinidae	4	3	s_xs	b_o	h_b	f_z	100
Brachinus crepitans	(Linné, 1758)	Carabidae	11	8	s_m	b_o	h_b	f_z	200
Brachinus explodens	Duftschmid, 1812	Carabidae	13	12	s_m	b_o	h_b	f_z	300
Brachonyx pineti	(Paykull, 1792)	Curculionidae	5	5	s_xs	b_w	h_v	f_p	200
Brachyderes incanus	(Linné, 1758)	Curculionidae	5	3	s_m	b_w	h_v	f_p	150
Brachygluta fossulata	(Reichenbach, 1816)	Pselaphidae	9	9	s_xs	b_e	h_b	f_z	300
Brachygonus megerlei	(Lacordaire, 1835)	Elateridae	5	4	s_m	b_w	h_t	f_x	150
Brachypterolus pulicarius	(Linné, 1758)	Kateretidae	9	6	s_xs	b_e	h_v	f_p	100
Brachypterus glaber	(Newman, 1834)	Kateretidae	6	6	s_xs	b_e	h_v	f_p	100
Brachypterus urticae	(Fabricius, 1792)	Kateretidae	9	9	s_xs	b_e	h_v	f_p	200
Bradybatus kellneri	Bach, 1854	Curculionidae	8	8	s_s	b_w	h_v	f_p	200
Bradycellus verbasci	(Duftschmid, 1812)	Carabidae	8	6	s_s	b_o	h_b	f_p	300
Bromius obscurus	(Linné, 1758)	Chrysomelidae	7	6	s_s	b_o	h_v	f_p	300
Broscus cephalotes	(Linné, 1758)	Carabidae	5	5	s_l	b_o	h_b	f_z	>300
Bruchela rufipes	(A. G. Olivier, 1790)	Anthribidae	5	5	s_xs	b_o	h_v	f_p	200
Bruchela suturalis	(Fabricius, 1792)	Anthribidae	5	4	s_xs	b_o	h_v	f_p	100
Bruchidius marginalis	(Fabricius, 1776)	Bruchidae	8	8	s_s	b_o	h_v	f_p	200
Bruchidius varius	(A. G. Olivier, 1795)	Chrysomelidae	4	4	s_xs	b_o	h_v	f_p	150
Bruchidius villosus	(Fabricius, 1792)	Bruchidae	11	10	s_xs	b_o	h_v	f_p	200
Bruchus atomarius	(Linné, 1761)	Bruchidae	4	4	s_xs	b_o	h_v	f_p	200
Bruchus loti	Paykull, 1800	Bruchidae	4	3	s_xs	b_o	h_v	f_p	100
Bruchus luteicornis	Illiger, 1794	Chrysomelidae	10	7	s_xs	b_o	h_v	f_p	200
Bruchus rufimanus	Boheman, 1833	Chrysomelidae	8	7	s_s	b_o	h_v	f_p	200
Bryaxis puncticollis	(Denny, 1825)	Staphylinidae	4	4	s_xs	b_e	h_b	f_z	200
Brychius elevatus	(Panzer, 1793)	Haliplidae	4	2	s_s	b_f	h_w	f_p	150
Byctiscus populi	(Linné, 1758)	Attelabidae	5	5	s_s	b_e	h_v	f_p	200
Byrrhus fasciatus	(Forster, 1771)	Byrrhidae	5	5	s_m	b_e	h_b	f_p	150
Byrrhus pilula	(Linné, 1758)	Byrrhidae	4	4	s_m	b_e	h_b	f_p	150

Byturus ochraceus	(Scriba, 1790)	Byturidae	10	9	s_s	b_e	h_v	f_p	300
Caenorhinus mannerheimii	(Hummel, 1823)	Attelabidae	4	2	s_s	b_w	h_v	f_p	100
Cafius xantholoma	(Gravenhorst, 1806)	Staphylinidae	4	3	s_m	b_f	h_b	f_z	>300
Calamobius filum	(P. Rossi, 1790)	Cerambycidae	4	4	s_m	b_o	h_v	f_p	>300
Calathus cinctus	(Motschulsky, 1850)	Carabidae	7	6	s_m	b_o	h_b	f_z	300
Calathus fuscipes	(Goeze, 1777)	Carabidae	8	8	s_l	b_o	h_b	f_z	>300
Calathus melanocephalus	(Linné, 1758)	Carabidae	10	10	s_m	b_o	h_b	f_z	300
Callidium coriaceum	(Paykull, 1800)	Cerambycidae	4	3	s_l	b_w	h_t	f_x	100
Callidium violaceum	(Fabricius, 1775)	Cerambycidae	7	5	s_l	b_w	h_t	f_x	300
Calodromius spilotus	(Illiger, 1798)	Carabidae	4	4	s_s	b_w	h_v	f_z	200
Calomicrus circumfusus	(Marsham, 1802)	Chrysomelidae	9	7	s_s	b_o	h_v	f_p	>300
Calomicrus pinicola	(Duftschmid, 1825)	Chrysomelidae	6	5	s_s	b_w	h_v	f_p	200
Calosoma inquisitor	(Linné, 1758)	Carabidae	4	4	s_l	b_w	h_v	f_z	100
Calvia decemguttata	(Linné, 1767)	Coccinellidae	10	9	s_s	b_w	h_v	f_z	200
Calvia quatuordecimguttata	(Linné, 1758)	Coccinellidae	7	7	s_s	b_e	h_v	f_z	300
Cantharis cryptica	Ashe, 1947	Cantharidae	7	6	s_m	b_w	h_v	f_z	150
Cantharis decipiens	Baudi di Selve, 1872	Cantharidae	6	5	s_m	b_w	h_v	f_z	200
Cantharis figurata	Mannerheim, 1843	Cantharidae	12	11	s_m	b_w	h_v	f_z	200
Cantharis flavilabris	Fallén, 1807	Cantharidae	8	8	s_m	b_f	h_v	f_z	>300
Cantharis fusca	Linné, 1758	Cantharidae	4	4	s_l	b_o	h_v	f_z	200
Cantharis lateralis	Linné, 1758	Cantharidae	6	5	s_m	b_o	h_v	f_z	300
Cantharis livida	Linné, 1758	Cantharidae	5	5	s_l	b_e	h_v	f_z	300
Cantharis nigra	DeGeer, 1774	Cantharidae	6	5	s_m	b_w	h_v	f_z	300
Cantharis nigricans	(O.F. Müller, 1776)	Cantharidae	5	4	s_m	b_w	h_v	f_z	150
Cantharis obscura	Linné, 1758	Cantharidae	8	8	s_m	b_w	h_v	f_z	200
Cantharis pagana	Rosenhauer, 1847	Cantharidae	5	4	s_m	b_w	h_v	f_z	300
Cantharis pallida	Goeze, 1777	Cantharidae	5	4	s_m	b_w	h_v	f_z	200
Cantharis paludosa	Fallén, 1807	Cantharidae	8	7	s_s	b_f	h_v	f_z	300
Cantharis paradoxa	Hicker, 1960	Cantharidae	4	4	s_m	b_w	h_v	f_z	200
Cantharis pellucida	Fabricius, 1792	Cantharidae	11	11	s_l	b_w	h_v	f_z	300
Cantharis rufa	Linné, 1758	Cantharidae	7	7	s_m	b_o	h_v	f_z	150
Cantharis rustica	Fallén, 1807	Cantharidae	15	13	s_l	b_o	h_v	f_z	300
Cantharis tristis	Fabricius, 1798	Cantharidae	9	9	s_m	b_w	h_v	f_z	150
Carabus alpestris	J. Sturm, 1815	Carabidae	5	5	s_l	b_o	h_b	f_z	100
Carabus auronitens	Fabricius, 1792	Carabidae	12	11	s_xl	b_w	h_b	f_z	>300
Carabus coriaceus	Linné, 1758	Carabidae	5	5	s_xl	b_w	h_b	f_z	150
Carabus germarii	J. Sturm, 1815	Carabidae	5	5	s_xl	b_w	h_b	f_z	150
Carabus glabratus	Paykull, 1790	Carabidae	4	3	s_xl	b_w	h_b	f_z	100
Carabus granulatus	Linné, 1758	Carabidae	10	10	s_l	b_w	h_b	f_z	>300
Carabus hortensis	Linné, 1758	Carabidae	4	3	s_xl	b_w	h_b	f_z	200
Carabus nemoralis	O. F. Müller, 1764	Carabidae	6	6	s_xl	b_e	h_b	f_z	300
Carabus problematicus	Herbst, 1786	Carabidae	14	13	s_xl	b_w	h_b	f_z	>300
Carabus sylvestris	Panzer, 1793	Carabidae	6	6	s_xl	b_w	h_b	f_z	200
Carabus violaceus	Linné, 1758	Carabidae	7	7	s_xl	b_w	h_b	f_z	>300
Carcinops pumilio	(Erichson, 1834)	Histeridae	4	3	s_xs	b_e	h_f	f_z	150
Cardiophorus erichsoni	Buysson, 1901	Elateridae	13	8	s_m	b_o	h_v	f_z	150
Carpelimus bilineatus	Stephens, 1834	Staphylinidae	16	14	s_s	b_f	h_b	f_p	200

Carpelimus corticinus	(Gravenhorst, 1806)	Staphylinidae	14	14	s_xs	b_f	h_b	f_p	300
Carpelimus elongatulus	(Erichson, 1839)	Staphylinidae	19	16	s_xs	b_f	h_b	f_p	300
Carpelimus fuliginosus	(Gravenhorst, 1802)	Staphylinidae	5	5	s_xs	b_e	h_f	f_s	200
Carpelimus gracilis	(Mannerheim, 1830)	Staphylinidae	6	5	s_xs	b_f	h_b	f_p	200
Carpelimus halophilus	(Kiesenwetter, 1844)	Staphylinidae	12	4	s_xs	b_f	h_b	f_p	100
Carpelimus impressus	(Lacordaire, 1835)	Staphylinidae	5	4	s_xs	b_f	h_b	f_p	150
Carpelimus lindrothi	(Palm, 1943)	Staphylinidae	5	3	s_xs	b_f	h_b	f_p	100
Carpelimus manchuricus	(Roubal, 1946)	Staphylinidae	4	4	s_xs	b_f	h_b	f_p	100
Carpelimus obesus	(Kiesenwetter, 1844)	Staphylinidae	9	5	s_xs	b_f	h_b	f_p	150
Carpelimus punctatellus	(Erichson, 1840)	Staphylinidae	4	2	s_xs	b_o	h_b	f_p	200
Carpelimus pusillus	(Gravenhorst, 1802)	Staphylinidae	10	10	s_xs	b_o	h_f	f_s	200
Carpelimus rivularis	(Motschulsky, 1860)	Staphylinidae	6	6	s_xs	b_f	h_b	f_p	150
Carpelimus similis	(Smetana, 1967)	Staphylinidae	7	5	s_s	b_f	h_b	f_p	300
Carpelimus subtilis	(Erichson, 1839)	Staphylinidae	9	5	s_xs	b_f	h_b	f_p	150
Carphacis striatus	(A. G. Olivier, 1795)	Staphylinidae	6	5	s_s	b_w	h_t	f_z	150
Cartodere constricta	(Gyllenhal, 1827)	Latridiidae	5	5	s_xs	b_w	h_b	f_m	300
Cassida canaliculata	Laicharting, 1781	Chrysomelidae	4	4	s_m	b_o	h_v	f_p	150
Cassida denticollis	Suffrian, 1844	Chrysomelidae	5	5	s_m	b_o	h_v	f_p	200
Cassida flaveola	Thunberg, 1794	Chrysomelidae	5	5	s_s	b_e	h_v	f_p	300
Cassida nebulosa	Linné, 1758	Chrysomelidae	4	4	s_m	b_o	h_v	f_p	150
Cassida rubiginosa	O. F. Müller, 1776	Chrysomelidae	15	14	s_m	b_o	h_v	f_p	300
Cassida sanguinosa	Suffrian, 1844	Chrysomelidae	7	5	s_m	b_o	h_v	f_p	100
Cassida stigmatica	Suffrian, 1844	Chrysomelidae	6	6	s_s	b_o	h_v	f_p	150
Cassida vibex	Linné, 1767	Chrysomelidae	12	11	s_m	b_w	h_v	f_p	>300
Cassida viridis	Linné, 1758	Chrysomelidae	4	3	s_m	b_o	h_v	f_p	300
Catapion meieri	(Desbrochers des Loges, 1	Brentidae	5	5	s_xs	b_o	h_v	f_p	200
Catapion seniculus	(Kirby, 1808)	Apionidae	7	6	s_xs	b_e	h_v	f_p	300
Cathormiocerus spinosus	(Goeze, 1777)	Curculionidae	4	4	s_xs	b_o	h_v	f_p	200
Catops fuliginosus	Erichson, 1837	Cholevidae	6	5	s_s	b_e	h_n	f_n	150
Catops neglectus	Kraatz, 1852	Leiodidae	4	3	s_s	b_w	h_n	f_n	200
Catops picipes	(Fabricius, 1787)	Leiodidae	7	7	s_s	b_w	h_n	f_n	300
Catops tristis	(Panzer, 1794)	Leiodidae	6	6	s_s	b_e	h_n	f_n	300
Cephennium gallicum	Ganglbauer, 1899	Scydmaenidae	5	5	s_xs	b_w	h_b	f_z	150
Cephennium thoracicum	P. W. J. Müller & Kunze,	Scydmaenidae	6	4	s_xs	b_w	h_b	f_z	150
Cerambyx scopolii	Füessly, 1775	Cerambycidae	6	6	s_xl	b_w	h_t	f_x	200
Ceratapion gibbirostre	(Gyllenhal, 1813)	Brentidae	11	11	s_xs	b_o	h_v	f_p	200
Ceratapion onopordi	(Kirby, 1808)	Brentidae	9	8	s_xs	b_e	h_v	f_p	200
Ceratomegilla alpina	(A. Villa & G. B. Villa,	Coccinellidae	4	3	s_s	b_o	h_v	f_z	100
Ceratomegilla notata	(Laicharting, 1781)	Coccinellidae	14	8	s_s	b_o	h_v	f_z	>300
Cercyon analis	(Paykull, 1798)	Hydrophilidae	10	10	s_xs	b_e	h_f	f_s	300
Cercyon bifenestratus	Küster, 1851	Hydrophilidae	7	4	s_xs	b_f	h_b	f_s	100
Cercyon haemorrhoidalis	(Fabricius, 1775)	Hydrophilidae	4	4	s_xs	b_e	h_f	f_s	300
Cercyon lateralis	(Marsham, 1802)	Hydrophilidae	4	4	s_xs	b_e	h_f	f_s	100
Cercyon littoralis	(Gyllenhal, 1808)	Hydrophilidae	4	4	s_xs	b_f	h_b	f_s	>300
Cercyon marinus	C. G. Thomson, 1853	Hydrophilidae	7	6	s_xs	b_f	h_b	f_s	>300
Cercyon melanocephalus	(Linné, 1758)	Hydrophilidae	7	6	s_xs	b_e	h_f	f_c	100
Cercyon quisquilius	(Linné, 1760)	Hydrophilidae	8	7	s_xs	b_e	h_f	f_c	300

Cercyon sternalis	Sharp, 1918	Hydrophilidae	5	5	s_xs	b_f	h_b	f_s	200
Cercyon unipunctatus	(Linné, 1758)	Hydrophilidae	4	4	s_xs	b_e	h_f	f_c	300
Cercyon ustulatus	(Preyssler, 1790)	Hydrophilidae	5	4	s_xs	b_f	h_b	f_s	150
Cerylon deplanatum	Gyllenhal, 1827	Cerylonidae	4	2	s_xs	b_w	h_t	f_z	100
Cerylon fagi	C. N. F. Brisout de Barne	Cerylonidae	6	6	s_xs	b_w	h_t	f_z	200
Cerylon ferrugineum	Stephens, 1830	Cerylonidae	9	6	s_xs	b_w	h_t	f_z	200
Cerylon histeroides	(Fabricius, 1792)	Cerylonidae	13	11	s_xs	b_w	h_t	f_z	300
Cetonia aurata	(Linné, 1761)	Scarabaeidae	9	9	s_l	b_w	h_t	f_x	>300
Ceutorhynchus atomus	Boheman, 1845	Curculionidae	4	3	s_xs	b_o	h_v	f_p	100
Ceutorhynchus cochleariae	(Gyllenhal, 1813)	Curculionidae	7	6	s_xs	b_f	h_v	f_p	200
Ceutorhynchus constrictus	(Marsham, 1802)	Curculionidae	6	4	s_xs	b_w	h_v	f_p	300
Ceutorhynchus contractus	(Marsham, 1802)	Curculionidae	4	4	s_xs	b_e	h_v	f_p	200
Ceutorhynchus erysimi	(Fabricius, 1787)	Curculionidae	7	7	s_xs	b_e	h_v	f_p	300
Ceutorhynchus napi	Gyllenhal, 1837	Curculionidae	4	3	s_s	b_o	h_v	f_p	200
Ceutorhynchus pallidactylus	(Marsham, 1802)	Curculionidae	11	11	s_xs	b_e	h_v	f_p	200
Ceutorhynchus picitarsis	Gyllenhal, 1837	Curculionidae	5	5	s_s	b_o	h_v	f_p	100
Ceutorhynchus rhenanus	(Schultze, 1895)	Curculionidae	5	4	s_xs	b_o	h_v	f_p	100
Ceutorhynchus roberti	Gyllenhal, 1837	Curculionidae	5	4	s_s	b_o	h_v	f_p	200
Ceutorhynchus rusticus	Gyllenhal, 1837	Curculionidae	4	4	s_s	b_o	h_v	f_p	100
Ceutorhynchus scapularis	Gyllenhal, 1837	Curculionidae	5	4	s_xs	b_f	h_v	f_p	150
Ceutorhynchus sulcicollis	(Paykull, 1800)	Curculionidae	4	3	s_xs	b_o	h_v	f_p	200
Ceutorhynchus turbatus	Schultze, 1903	Curculionidae	7	5	s_xs	b_o	h_v	f_p	100
Ceutorhynchus typhae	(Herbst, 1795)	Curculionidae	5	4	s_xs	b_e	h_v	f_p	200
Chaetarthria seminulum	(Herbst, 1797)	Hydrophilidae	9	7	s_xs	b_f	h_w	f_s	>300
Chaetocnema concinna	(Marsham, 1802)	Chrysomelidae	6	6	s_xs	b_e	h_v	f_p	200
Chaetocnema hortensis	(Geoffroy, 1785)	Chrysomelidae	6	6	s_xs	b_e	h_v	f_p	300
Chaetocnema picipes	Stephens, 1831	Chrysomelidae	4	4	s_xs	b_o	h_v	f_p	100
Charagmus gressorius	(Fabricius, 1792)	Curculionidae	5	5	s_m	b_o	h_v	f_p	100
Charopus flavipes	(Paykull, 1798)	Melyridae	5	5	s_xs	b_o	h_v	f_z	200
Charopus pallipes	(A. G. Olivier, 1790)	Melyridae	4	4	s_xs	b_o	h_v	f_z	100
Chilocorus bipustulatus	(Linné, 1758)	Coccinellidae	5	5	s_s	b_e	h_v	f_z	150
Chilocorus renipustulatus	(L. G. Scriba, 1791)	Coccinellidae	5	4	s_s	b_w	h_v	f_z	300
Chlaenius vestitus	(Paykull, 1790)	Carabidae	8	7	s_m	b_f	h_b	f_z	>300
Chlorophanus viridis	(Linné, 1758)	Curculionidae	7	6	s_m	b_f	h_v	f_p	300
Chlorophorus sartor	(O. F. Müller, 1766)	Cerambycidae	4	2	s_m	b_w	h_t	f_x	300
Choleva agilis	(Illiger, 1798)	Cholevidae	5	4	s_s	b_e	h_n	f_n	100
Chrysanthia viridissima	(Linné, 1758)	Oedemeridae	6	5	s_m	b_w	h_t	f_x	300
Chrysobothris affinis	(Fabricius, 1794)	Buprestidae	4	4	s_l	b_w	h_t	f_x	>300
Chrysobothris chrysostigma	(Linné, 1758)	Buprestidae	4	3	s_l	b_w	h_t	f_x	100
Chrysolina coerulans	(L. G. Scriba, 1791)	Chrysomelidae	5	5	s_m	b_f	h_v	f_p	300
Chrysolina fastuosa	(Scopoli, 1763)	Chrysomelidae	7	6	s_s	b_o	h_v	f_p	>300
Chrysolina haemoptera	(Linné, 1758)	Chrysomelidae	4	4	s_m	b_o	h_v	f_p	150
Chrysolina herbacea	(Duftschmid, 1825)	Chrysomelidae	7	7	s_m	b_f	h_v	f_p	300
Chrysolina hyperici	(Forster, 1771)	Chrysomelidae	14	11	s_m	b_o	h_v	f_p	300
Chrysolina marginata	(Linné, 1758)	Chrysomelidae	5	1	s_m	b_o	h_v	f_p	100
Chrysolina oricalcia	(O. F. Müller, 1776)	Chrysomelidae	4	4	s_m	b_o	h_v	f_p	150
Chrysolina polita	(Linné, 1758)	Chrysomelidae	10	10	s_m	b_f	h_v	f_p	300

Chrysolina sanguinolenta	(Linné, 1758)	Chrysomelidae	4	4	s_m	b_o	h_v	f_p	100
Chrysolina staphylaea	(Linné, 1758)	Chrysomelidae	4	4	s_m	b_o	h_v	f_p	150
Chrysolina varians	(Schaller, 1783)	Chrysomelidae	17	16	s_s	b_e	h_v	f_p	300
Chrysomela populi	Linné, 1758	Chrysomelidae	6	6	s_l	b_w	h_v	f_p	150
Chrysomela vigintipunctata	(Scopoli, 1763)	Chrysomelidae	11	8	s_m	b_w	h_v	f_p	200
Cicindela campestris	Linné, 1758	Carabidae	12	12	s_l	b_o	h_b	f_z	>300
Cicindela hybrida	Linné, 1758	Carabidae	8	6	s_l	b_o	h_b	f_z	>300
Cidnopus pilosus	(Leske, 1785)	Elateridae	14	12	s_m	b_w	h_v	f_p	300
Cilea silphoides	(Linné, 1767)	Staphylinidae	7	6	s_s	b_e	h_f	f_z	200
Cimberis attelaboides	(Fabricius, 1787)	Nemonychidae	4	4	s_s	b_w	h_v	f_p	200
Cionus alauda	(Herbst, 1784)	Curculionidae	4	4	s_s	b_w	h_v	f_p	150
Cionus hortulanus	(Geoffroy, 1785)	Curculionidae	7	6	s_s	b_e	h_v	f_p	300
Cionus olens	(Fabricius, 1792)	Curculionidae	6	5	s_s	b_o	h_v	f_p	100
Cionus tuberculosus	(Scopoli, 1763)	Curculionidae	13	10	s_s	b_w	h_v	f_p	300
Cis boleti	(Scopoli, 1763)	Ciidae	6	6	s_s	b_w	h_t	f_m	300
Cis castaneus	(Herbst, 1793)	Cisidae	13	10	s_xs	b_w	h_t	f_m	200
Cis comptus	Gyllenhal, 1827	Ciidae	4	4	s_xs	b_w	h_t	f_m	100
Cis dentatus	Mellié, 1848	Ciidae	6	6	s_xs	b_w	h_t	f_m	200
Cis fagi	Waltl, 1839	Ciidae	8	7	s_xs	b_w	h_t	f_m	200
Cis festivus	(Panzer, 1793)	Cisidae	4	4	s_xs	b_w	h_t	f_m	200
Cis glabratus	Mellié, 1848	Ciidae	8	6	s_xs	b_w	h_t	f_m	150
Cis micans	(Fabricius, 1792)	Ciidae	10	10	s_xs	b_w	h_t	f_m	200
Clambus armadillo	(DeGeer, 1774)	Clambidae	6	6	s_xs	b_e	h_f	f_m	150
Clambus minutus	(J. Sturm, 1807)	Clambidae	4	2	s_xs	b_f	h_f	f_m	100
Clambus nigrellus	Reitter, 1914	Clambidae	5	3	s_xs	b_f	h_f	f_m	100
Clambus punctulum	(L. Beck, 1817)	Clambidae	6	5	s_xs	b_w	h_f	f_m	150
Clambus simsoni	Blackburn, 1902	Clambidae	4	4	s_xs	b_e	h_f	f_m	150
Clanoptilus elegans	(A. G. Olivier, 1790)	Malachiidae	5	5	s_s	b_o	h_v	f_z	200
Clanoptilus geniculatus	(Germar, 1824)	Melyridae	4	2	s_s	b_o	h_v	f_z	200
Cleopus solani	(Fabricius, 1792)	Curculionidae	5	2	s_xs	b_o	h_v	f_p	100
Clitostethus arcuatus	(P. Rossi, 1794)	Coccinellidae	4	3	s_xs	b_o	h_v	f_z	150
Clivina collaris	(Herbst, 1784)	Carabidae	4	4	s_s	b_f	h_b	f_z	300
Clivina fossor	(Linné, 1758)	Carabidae	11	11	s_s	b_e	h_b	f_z	300
Clytra quadripunctata	(Linné, 1758)	Chrysomelidae	13	11	s_m	b_w	h_n	f_p	300
Clytus arietis	(Linné, 1758)	Cerambycidae	9	9	s_m	b_w	h_t	f_x	300
Clytus lama	Mulsant, 1847	Cerambycidae	6	4	s_l	b_w	h_t	f_x	100
Coccidula rufa	(Herbst, 1783)	Coccinellidae	12	9	s_xs	b_e	h_v	f_z	300
Coccidula scutellata	(Herbst, 1783)	Coccinellidae	10	8	s_xs	b_f	h_v	f_z	>300
Coccinella quinquepunctata	Linné, 1758	Coccinellidae	6	6	s_s	b_o	h_v	f_z	300
Coccinella septempunctata	Linné, 1758	Coccinellidae	10	9	s_m	b_e	h_v	f_z	300
Coccinella undecimpunctata	Linné, 1758	Coccinellidae	7	5	s_s	b_o	h_v	f_z	100
Coccinula quatuordecimpustulata	(Linné, 1758)	Coccinellidae	13	12	s_s	b_o	h_v	f_z	300
Coelostoma orbiculare	(Fabricius, 1775)	Hydrophilidae	4	4	s_s	b_f	h_w	f_s	150
Colenis immunda	(J. Sturm, 1807)	Leiodidae	5	4	s_xs	b_w	h_p	f_m	150
Colon serripes	(C. R. Sahlberg, 1822)	Leiodidae	5	5	s_xs	b_w	h_p	f_m	100
Colydium elongatum	(Fabricius, 1787)	Zopheridae	8	8	s_m	b_w	h_t	f_z	300

Colymbetes fuscus	(Linné, 1758)	Dytiscidae	9	8	s_l	b_f	h_w	f_z	300
Conopalpus brevicollis	Kraatz, 1855	Melandryidae	7	6	s_s	b_w	h_t	f_x	100
Conopalpus testaceus	(A. G. Olivier, 1790)	Melandryidae	9	7	s_m	b_w	h_t	f_x	200
Contacyphon coarctatus	(Paykull, 1799)	Scirtidae	6	6	s_s	b_f	h_w	f_s	200
Contacyphon laevipennis	(Tournier, 1868)	Scirtidae	4	4	s_s	b_f	h_w	f_s	150
Contacyphon padi	(Linné, 1758)	Scirtidae	5	5	s_xs	b_f	h_w	f_s	300
Contacyphon variabilis	(Thunberg, 1787)	Scirtidae	16	12	s_s	b_f	h_w	f_s	300
Coprophilus striatulus	(Fabricius, 1792)	Staphylinidae	8	8	s_m	b_e	h_f	f_s	200
Coptocephala rubicunda	(Laicharting, 1781)	Chrysomelidae	4	3	s_s	b_o	h_v	f_p	150
Coraebus elatus	(Fabricius, 1787)	Buprestidae	8	4	s_m	b_o	h_v	f_p	>300
Cordalia obscura	(Gravenhorst, 1802)	Staphylinidae	5	4	s_xs	b_e	h_f	f_z	150
Cordylepherus viridis	(Fabricius, 1787)	Melyridae	7	4	s_s	b_o	h_v	f_z	150
Corticaria alleni	Johnson, 1974	Latridiidae	8	4	s_xs	b_w	h_t	f_m	150
Corticaria elongata	(Gyllenhal, 1827)	Latridiidae	5	5	s_xs	b_e	h_e	f_m	150
Corticaria fulva	(Comolli, 1837)	Latridiidae	5	4	s_xs	b_e	h_e	f_m	300
Corticaria impressa	(A. G. Olivier, 1790)	Latridiidae	5	5	s_xs	b_e	h_e	f_m	300
Corticaria longicollis	(Zetterstedt, 1838)	Latridiidae	5	3	s_xs	b_w	h_t	f_m	150
Corticaria obscura	C. N. F. Brisout de Barne	Latridiidae	9	5	s_xs	b_o	h_v	f_m	200
Corticaria serrata	(Paykull, 1798)	Latridiidae	9	9	s_xs	b_e	h_f	f_m	300
Corticarina similata	(Gyllenhal, 1827)	Latridiidae	5	5	s_xs	b_e	h_e	f_m	200
Corticarina truncatella	(Mannerheim, 1844)	Latridiidae	9	6	s_xs	b_o	h_b	f_m	100
Corticeus bicolor	(A. G. Olivier, 1790)	Tenebrionidae	6	5	s_s	b_w	h_t	f_z	200
Corticeus unicolor	Piller & Mitterpacher, 17	Tenebrionidae	8	7	s_m	b_w	h_t	f_z	300
Cortinicara gibbosa	(Herbst, 1793)	Latridiidae	13	12	s_xs	b_e	h_e	f_m	300
Cortodera femorata	(Fabricius, 1787)	Cerambycidae	5	3	s_m	b_w	h_t	f_x	100
Corylophus cassidoides	(Marsham, 1802)	Corylophidae	13	10	s_xs	b_f	h_f	f_z	200
Coxelus pictus	(J. Sturm, 1807)	Zopheridae	5	2	s_xs	b_w	h_t	f_m	100
Cratosilis denticollis	(Schummel, 1844)	Cantharidae	6	5	s_m	b_f	h_v	f_z	200
Creophilus maxillosus	(Linné, 1758)	Staphylinidae	6	4	s_l	b_e	h_f	f_z	150
Crepidodera aurata	(Marsham, 1802)	Chrysomelidae	12	12	s_s	b_e	h_v	f_p	300
Crepidodera aurea	(Geoffroy, 1785)	Chrysomelidae	10	10	s_s	b_w	h_v	f_p	300
Crepidodera fulvicornis	(Fabricius, 1792)	Chrysomelidae	9	9	s_xs	b_w	h_v	f_p	300
Crepidodera plutus	(Latreille, 1804)	Chrysomelidae	5	3	s_xs	b_w	h_v	f_p	150
Crioceris asparagi	(Linné, 1758)	Chrysomelidae	6	5	s_s	b_o	h_v	f_p	200
Crioceris duodecimpunctata	(Linné, 1758)	Chrysomelidae	5	4	s_s	b_o	h_v	f_p	300
Cryphalus piceae	(Ratzeburg, 1837)	Curculionidae	4	4	s_xs	b_w	h_t	f_x	100
Crypticus quisquilius	(Linné, 1760)	Tenebrionidae	12	8	s_s	b_o	h_b	f_s	300
Cryptocephalus aureolus	Suffrian, 1847	Chrysomelidae	5	5	s_m	b_o	h_v	f_p	300
Cryptocephalus bameuli	Duhaldeborde, 1999	Chrysomelidae	11	7	s_s	b_o	h_v	f_p	200
Cryptocephalus bilineatus	(Linné, 1767)	Chrysomelidae	9	6	s_xs	b_o	h_v	f_p	100
Cryptocephalus bipunctatus	(Linné, 1758)	Chrysomelidae	14	13	s_s	b_o	h_v	f_p	300
Cryptocephalus coryli	(Linné, 1758)	Chrysomelidae	4	4	s_m	b_w	h_v	f_p	300
Cryptocephalus fulvus	(Goeze, 1777)	Chrysomelidae	7	7	s_xs	b_o	h_v	f_p	200
Cryptocephalus labiatus	(Linné, 1760)	Chrysomelidae	4	3	s_xs	b_w	h_v	f_p	300
Cryptocephalus moraei	(Linné, 1758)	Chrysomelidae	9	8	s_s	b_o	h_v	f_p	200
Cryptocephalus nitidus	(Linné, 1758)	Chrysomelidae	10	10	s_s	b_w	h_v	f_p	300
Cryptocephalus ocellatus	Drapiez, 1819	Chrysomelidae	4	4	s_s	b_w	h_v	f_p	150

Cryptocephalus populi	Suffrian, 1848	Chrysomelidae	5	2	s_xs	b_w	h_v	f_p	150
Cryptocephalus pusillus	Fabricius, 1777	Chrysomelidae	10	9	s_xs	b_w	h_v	f_p	300
Cryptocephalus pygmaeus	Fabricius, 1792	Chrysomelidae	8	6	s_xs	b_o	h_v	f_p	200
Cryptocephalus sexpunctatus	(Linné, 1758)	Chrysomelidae	4	4	s_s	b_w	h_v	f_p	150
Cryptocephalus violaceus	Laicharting, 1781	Chrysomelidae	5	3	s_s	b_w	h_v	f_p	300
Cryptocephalus vittatus	Fabricius, 1775	Chrysomelidae	4	4	s_s	b_o	h_v	f_p	150
Cryptolestes duplicatus	(Waltl, 1839)	Laemophloeidae	5	5	s_xs	b_w	h_t	f_z	200
Cryptophagus dentatus	(Herbst, 1793)	Cryptophagidae	4	4	s_xs	b_e	h_e	f_m	100
Cryptophagus distinguendus	J. Sturm, 1845	Cryptophagidae	6	6	s_xs	b_e	h_e	f_m	100
Cryptophagus lycoperdi	(Scopoli, 1763)	Cryptophagidae	6	6	s_xs	b_e	h_p	f_m	200
Cryptophagus pallidus	J. Sturm, 1845	Cryptophagidae	6	6	s_xs	b_e	h_f	f_m	100
Cryptophagus pubescens	J. Sturm, 1845	Cryptophagidae	6	6	s_xs	b_e	h_n	f_m	150
Cryptophagus scanicus	(Linné, 1758)	Cryptophagidae	4	4	s_xs	b_e	h_e	f_m	100
Cryptophagus schmidtii	J. Sturm, 1845	Cryptophagidae	5	4	s_xs	b_o	h_n	f_m	150
Cryptophilus obliteratus	Reitter, 1874	Erotylidae	9	4	s_xs	b_o	h_f	f_m	100
Cryptopleurum minutum	(Fabricius, 1775)	Hydrophilidae	10	9	s_xs	b_e	h_f	f_s	200
Cryptorhynchus lapathi	(Linné, 1758)	Curculionidae	6	4	s_m	b_w	h_t	f_x	300
Ctenicera pectinicornis	(Linné, 1758)	Elateridae	9	9	s_l	b_f	h_v	f_p	300
Cteniopus sulphureus	(Linné, 1758)	Tenebrionidae	10	7	s_m	b_o	h_v	f_p	>300
Ctesias serra	(Fabricius, 1792)	Dermestidae	4	4	s_s	b_w	h_n	f_n	200
Curculio elephas	(Gyllenhal, 1835)	Curculionidae	8	6	s_m	b_w	h_v	f_p	300
Curculio pellitus	(Boheman, 1843)	Curculionidae	4	4	s_m	b_w	h_v	f_p	150
Curculio venosus	(Gravenhorst, 1807)	Curculionidae	5	4	s_m	b_w	h_v	f_p	150
Cyanapion columbinum	(Germar, 1817)	Brentidae	7	5	s_xs	b_o	h_v	f_p	200
Cyanapion platalea	(Germar, 1817)	Brentidae	4	3	s_xs	b_e	h_v	f_p	150
Cyanapion spencii	(Kirby, 1808)	Brentidae	7	6	s_xs	b_o	h_v	f_p	300
Cychramus luteus	(Fabricius, 1787)	Nitidulidae	12	11	s_s	b_w	h_t	f_m	300
Cychrus attenuatus	Fabricius, 1792	Carabidae	8	7	s_l	b_w	h_b	f_z	200
Cychrus caraboides	(Linné, 1758)	Carabidae	6	6	s_l	b_w	h_b	f_z	>300
Cyclodinus humilis	(Germar, 1824)	Anthicidae	5	4	s_xs	b_f	h_b	f_z	100
Cyclorhipidion bodoanum	(Reitter, 1913)	Curculionidae	4	4	s_xs	b_w	h_t	f_m	150
Cymbiodyta marginella	(Fabricius, 1792)	Hydrophilidae	7	5	s_s	b_f	h_w	f_s	200
Cymindis vaporariorum	(Linné, 1758)	Carabidae	5	5	s_m	b_o	h_b	f_z	300
Cytilus sericeus	(Forster, 1771)	Byrrhidae	8	7	s_s	b_e	h_b	f_p	300
Dacne bipustulata	(Thunberg, 1781)	Erotylidae	8	8	s_xs	b_w	h_t	f_m	300
Dalopius marginatus	(Linné, 1758)	Elateridae	9	9	s_m	b_w	h_v	f_z	300
Danacea nigritarsis	(Küster, 1850)	Melyridae	14	10	s_s	b_o	h_v	f_z	200
Danacea pallipes	(Panzer, 1793)	Melyridae	5	5	s_s	b_o	h_v	f_z	100
Dascillus cervinus	(Linné, 1758)	Dascillidae	10	8	s_m	b_f	h_v	f_p	300
Dasytes aeratus	Stephens, 1830	Melyridae	7	7	s_s	b_w	h_t	f_z	100
Dasytes alpigradus	Kiesenwetter, 1863	Melyridae	10	7	s_s	b_w	h_t	f_z	100
Dasytes caeruleus	(DeGeer, 1774)	Melyridae	13	11	s_s	b_w	h_t	f_z	300
Dasytes niger	(Linné, 1761)	Melyridae	17	13	s_s	b_w	h_t	f_z	>300
Dasytes obscurus	Gyllenhal, 1813	Melyridae	5	5	s_s	b_w	h_t	f_z	200
Dasytes plumbeus	(O. F. Müller, 1776)	Melyridae	16	16	s_s	b_w	h_t	f_z	300
Dasytes virens	(Marsham, 1802)	Melyridae	12	9	s_s	b_w	h_t	f_z	150
Datonychus melanostictus	(Marsham, 1802)	Curculionidae	4	3	s_xs	b_f	h_v	f_p	100

Deleaster dichrous	(Gravenhorst, 1802)	Staphylinidae	18	14	s_m	b_f	h_b	f_z	>300
Demetrias atricapillus	(Linné, 1758)	Carabidae	7	6	s_s	b_o	h_v	f_z	200
Demetrias monostigma	Samouelle, 1819	Carabidae	7	6	s_s	b_f	h_v	f_z	300
Dendrophilus punctatus	(Herbst, 1792)	Histeridae	5	4	s_s	b_w	h_t	f_z	200
Dendrophilus pygmaeus	(Linné, 1758)	Histeridae	4	3	s_s	b_w	h_n	f_z	150
Denticollis linearis	(Linné, 1758)	Elateridae	9	9	s_m	b_w	h_t	f_x	300
Deporaus betulae	(Linné, 1758)	Attelabidae	10	10	s_s	b_e	h_v	f_p	300
Dermestes laniarius	Illiger, 1801	Dermestidae	5	4	s_m	b_o	h_f	f_n	>300
Dermestes lardarius	Linné, 1758	Dermestidae	5	5	s_m	b_e	h_f	f_n	300
Dermestes undulatus	Brahm, 1790	Dermestidae	4	4	s_m	b_w	h_f	f_n	100
Dermestoides sanguinicollis	(Fabricius, 1787)	Cleridae	4	4	s_m	b_w	h_t	f_z	100
Derocrepis rufipes	(Linné, 1758)	Chrysomelidae	12	12	s_s	b_o	h_v	f_p	200
Dexiogyia corticina	(Erichson, 1837)	Staphylinidae	6	4	s_xs	b_w	h_t	f_z	150
Diachromus germanus	(Linné, 1758)	Carabidae	4	3	s_m	b_o	h_b	f_p	200
Diaclina fagi	(Panzer, 1799)	Tenebrionidae	5	5	s_s	b_w	h_t	f_s	150
Diaperis boleti	(Linné, 1758)	Tenebrionidae	6	6	s_m	b_w	h_t	f_m	300
Dibolia rugulosa	L. Redtenbacher, 1849	Chrysomelidae	4	3	s_xs	b_o	h_v	f_p	100
Dibolia timida	(Illiger, 1803)	Chrysomelidae	4	3	s_s	b_o	h_v	f_p	100
Dienerella clathrata	(Mannerheim, 1844)	Latridiidae	4	3	s_xs	b_w	h_e	f_m	100
Dienerella vincenti	Johnson, 2007	Latridiidae	5	4	s_xs	b_e	h_e	f_m	100
Dinaraea aequata	(Erichson, 1837)	Staphylinidae	5	5	s_xs	b_w	h_t	f_z	200
Dinoptera collaris	(Linné, 1758)	Cerambycidae	14	14	s_m	b_w	h_t	f_x	300
Diplapion confluens	(Kirby, 1808)	Brentidae	4	3	s_xs	b_e	h_v	f_p	100
Diplocoelus fagi	(Chevrolat, 1837)	Biphyllidae	4	4	s_s	b_w	h_t	f_m	200
Dissoleucas niveirostris	(Fabricius, 1798)	Anthribidae	9	9	s_s	b_w	h_t	f_x	300
Dochmonota clancula	(Erichson, 1837)	Staphylinidae	5	2	s_xs	b_w	h_b	f_z	100
Dodecastichus geniculatus	(Germar, 1817)	Curculionidae	4	3	s_m	b_f	h_v	f_p	100
Dolichosoma lineare	(P. Rossi, 1794)	Melyridae	12	12	s_m	b_o	h_v	f_z	300
Domene scabricollis	(Erichson, 1840)	Staphylinidae	6	6	s_m	b_w	h_b	f_z	150
Donacia semicuprea	Panzer, 1796	Chrysomelidae	4	2	s_m	b_f	h_v	f_p	100
Donacia versicolorea	(Brahm, 1790)	Chrysomelidae	5	4	s_m	b_f	h_v	f_p	200
Donus ovalis	(Boheman, 1842)	Curculionidae	5	5	s_m	b_f	h_v	f_p	300
Dorcus parallelipipedus	(Linné, 1758)	Lucanidae	7	7	s_xl	b_w	h_t	f_x	300
Dorytomus ictor	(Herbst, 1795)	Curculionidae	7	4	s_s	b_w	h_v	f_p	150
Dorytomus longimanus	(Forster, 1771)	Curculionidae	9	7	s_m	b_w	h_v	f_p	200
Dorytomus nebulosus	(Gyllenhal, 1835)	Curculionidae	6	5	s_s	b_w	h_v	f_p	200
Dorytomus taeniatus	(Fabricius, 1781)	Curculionidae	7	7	s_s	b_w	h_v	f_p	200
Drilus concolor	Ahrens, 1812	Drilidae	4	4	s_s	b_o	h_v	f_z	200
Dromius quadrimaculatus	(Linné, 1758)	Carabidae	6	6	s_s	b_w	h_v	f_z	300
Dropephylla ioptera	(Stephens, 1834)	Staphylinidae	8	7	s_xs	b_w	h_t	f_z	200
Drusilla canaliculata	(Fabricius, 1787)	Staphylinidae	6	4	s_s	b_o	h_b	f_z	150
Dryocoetes autographus	(Ratzeburg, 1837)	Scolytidae	7	7	s_s	b_w	h_t	f_x	300
Dryocoetes villosus	(Fabricius, 1792)	Curculionidae	7	5	s_s	b_w	h_t	f_x	100
Dryophilus pusillus	(Gyllenhal, 1808)	Ptinidae	5	5	s_xs	b_w	h_t	f_x	150
Dryophthorus corticalis	(Paykull, 1792)	Dryophthoridae	4	3	s_s	b_w	h_t	f_x	100
Dryops luridus	(Erichson, 1847)	Dryopidae	4	4	s_s	b_f	h_w	f_p	300
Dyschirius angustatus	(Ahrens, 1830)	Carabidae	8	7	s_s	b_f	h_b	f_z	100

Dyschirius globosus	(Herbst, 1784)	Carabidae	10	9	s_xs	b_e	h_b	f_z	300
Dyschirius intermedius	Putzeys, 1846	Carabidae	4	2	s_s	b_f	h_b	f_z	100
Dyschirius obscurus	(Gyllenhal, 1827)	Carabidae	5	4	s_s	b_f	h_b	f_z	300
Dyschirius politus	(Dejean, 1825)	Carabidae	4	4	s_s	b_f	h_b	f_z	200
Dyschirius thoracicus	Fabricius, 1801	Carabidae	6	5	s_s	b_f	h_b	f_z	150
Dytiscus marginalis	Linné, 1758	Dytiscidae	4	4	s_xl	b_f	h_w	f_z	150
Ebaeus thoracicus	(Geoffroy, 1785)	Melyridae	5	4	s_xs	b_o	h_n	f_z	300
Echinodera hypocrita	(Boheman, 1837)	Curculionidae	5	5	s_s	b_w	h_t	f_x	200
Ectinus aterrimus	(Linné, 1761)	Elateridae	4	4	s_l	b_w	h_v	f_p	150
Elaphropus diabrachys	(Kolenati, 1845)	Carabidae	4	3	s_xs	b_f	h_b	f_z	300
Elaphropus parvulus	(Dejean, 1831)	Carabidae	11	8	s_xs	b_o	h_b	f_z	300
Elaphropus quadrisignatus	(Duftschmid, 1812)	Carabidae	4	3	s_xs	b_f	h_b	f_z	150
Elaphrus riparius	(Linné, 1758)	Carabidae	7	7	s_m	b_f	h_b	f_z	300
Elateroides dermestoides	(Linné, 1761)	Lymexylidae	11	10	s_l	b_w	h_t	f_x	>300
Eledona agricola	(Herbst, 1783)	Tenebrionidae	10	10	s_xs	b_w	h_t	f_m	300
Ellescus scanicus	(Paykull, 1792)	Curculionidae	7	4	s_xs	b_w	h_v	f_p	100
Elmis aenea	(P. W. J. Müller, 1806)	Elmidae	4	4	s_xs	b_f	h_w	f_p	300
Elmis maugetii	Latreille, 1802	Elmidae	4	4	s_xs	b_f	h_w	f_p	300
Elmis rioloides	(Kuwert, 1890)	Elmidae	4	3	s_xs	b_f	h_w	f_p	150
Elodes minuta	(Linné, 1767)	Scirtidae	10	8	s_s	b_f	h_w	f_s	300
Elodes pseudominuta	Klausnitzer, 1971	Scirtidae	4	2	s_s	b_f	h_w	f_s	100
Endomychus coccineus	(Linné, 1758)	Endomychidae	4	4	s_s	b_w	h_t	f_m	150
Enicmus rugosus	(Herbst, 1793)	Latridiidae	6	6	s_xs	b_e	h_e	f_m	>300
Ennearthron cornutum	(Gyllenhal, 1827)	Ciidae	7	7	s_xs	b_w	h_t	f_m	200
Enochrus affinis	(Thunberg, 1794)	Hydrophilidae	4	4	s_s	b_f	h_w	f_s	100
Enochrus bicolor	(Fabricius, 1792)	Hydrophilidae	8	6	s_s	b_f	h_w	f_s	150
Enochrus coarctatus	(Gredler, 1863)	Hydrophilidae	5	4	s_s	b_f	h_w	f_s	150
Enochrus melanocephalus	(A. G. Olivier, 1792)	Hydrophilidae	10	9	s_s	b_f	h_w	f_s	300
Enochrus ochropterus	(Marsham, 1802)	Hydrophilidae	8	5	s_s	b_f	h_w	f_s	200
Enochrus quadripunctatus	(Herbst, 1797)	Hydrophilidae	16	11	s_s	b_f	h_w	f_s	300
Enochrus testaceus	(Fabricius, 1801)	Hydrophilidae	7	6	s_m	b_f	h_w	f_s	300
Epaphius secalis	(Paykull, 1790)	Carabidae	9	6	s_s	b_f	h_b	f_z	300
Ephistemus globulus	(Paykull, 1798)	Cryptophagidae	7	6	s_xs	b_e	h_f	f_m	200
Ephistemus reitteri	T. L. Casey, 1900	Cryptophagidae	4	4	s_xs	b_o	h_f	f_m	100
Epitrix atropae	Foudras, 1861	Chrysomelidae	8	5	s_xs	b_w	h_v	f_p	150
Epuraea aestiva	(Linné, 1758)	Nitidulidae	8	6	s_s	b_e	h_n	f_z	200
Epuraea binotata	Reitter, 1873	Nitidulidae	13	8	s_xs	b_w	h_t	f_z	300
Epuraea distincta	(Grimmer, 1841)	Nitidulidae	4	3	s_xs	b_w	h_t	f_z	150
Epuraea marseuli	Reitter, 1873	Nitidulidae	5	5	s_s	b_w	h_t	f_z	200
Epuraea melanocephala	(Marsham, 1802)	Nitidulidae	7	6	s_xs	b_w	h_v	f_z	150
Epuraea muehli	Reitter, 1908	Nitidulidae	6	4	s_xs	b_w	h_t	f_z	150
Epuraea neglecta	(Heer, 1841)	Nitidulidae	5	3	s_xs	b_w	h_t	f_z	300
Epuraea pallescens	(Stephens, 1835)	Nitidulidae	5	5	s_xs	b_w	h_t	f_z	200
Epuraea thoracica	Tournier, 1872	Nitidulidae	4	3	s_s	b_w	h_t	f_z	150
Epuraea unicolor	(A. G. Olivier, 1790)	Nitidulidae	4	4	s_xs	b_e	h_e	f_z	100
Epuraea variegata	(Herbst, 1793)	Nitidulidae	5	5	s_xs	b_w	h_t	f_z	100
Erichsonius cinerascens	(Gravenhorst, 1802)	Staphylinidae	7	6	s_s	b_f	h_b	f_z	200

Ernobius abietis	(Fabricius, 1792)	Ptinidae	8	6	s_s	b_w	h_t	f_x	150
Ernobius angusticollis	(Ratzeburg, 1837)	Anobiidae	7	6	s_s	b_w	h_t	f_x	200
Ernobius longicornis	(J. Sturm, 1837)	Anobiidae	4	2	s_s	b_w	h_t	f_x	100
Ernobius mollis	(Linné, 1758)	Anobiidae	6	5	s_s	b_w	h_t	f_x	200
Ernobius nigrinus	(J. Sturm, 1837)	Anobiidae	7	4	s_s	b_w	h_t	f_x	150
Ernobius pini	(J. Sturm, 1837)	Ptinidae	4	3	s_xs	b_w	h_t	f_x	100
Ernoporus tiliae	(Panzer, 1793)	Curculionidae	6	5	s_xs	b_w	h_t	f_x	100
Esolus angustatus	(P. W. J. Müller, 1821)	Elmidae	4	4	s_xs	b_f	h_w	f_p	300
Esolus parallelepipedus	(P. W. J. Müller, 1806)	Elmidae	8	8	s_xs	b_f	h_w	f_p	300
Euaesthetus laeviusculus	Mannerheim, 1844	Staphylinidae	4	4	s_xs	b_f	h_b	f_z	100
Euaesthetus ruficapillus	Lacordaire, 1835	Staphylinidae	6	5	s_xs	b_f	h_b	f_z	200
Eubrychius velutus	(L. Beck, 1817)	Curculionidae	4	3	s_xs	b_f	h_v	f_p	100
Euconnus fimetarius	(Chaudoir, 1845)	Staphylinidae	5	4	s_xs	b_e	h_f	f_z	100
Euconnus rutilipennis	(P. W. J. Müller & Kunze,	Staphylinidae	6	5	s_xs	b_f	h_b	f_z	150
Euglenes oculatus	(Paykull, 1798)	Aderidae	6	6	s_xs	b_w	h_t	f_x	200
Euglenes pygmaeus	(DeGeer, 1775)	Aderidae	10	6	s_xs	b_w	h_t	f_x	300
Euplectus infirmus	Raffray, 1910	Staphylinidae	5	4	s_xs	b_w	h_t	f_z	150
Euplectus karstenii	Reichenbach, 1816	Pselaphidae	11	8	s_xs	b_w	h_t	f_z	300
Euplectus kirbii	Denny, 1825	Staphylinidae	5	4	s_xs	b_w	h_t	f_z	100
Euplectus nanus	(Reichenbach, 1816)	Staphylinidae	7	5	s_xs	b_w	h_t	f_z	300
Euplectus piceus	Motschulsky, 1835	Staphylinidae	6	3	s_xs	b_w	h_t	f_z	100
Euryusa castanoptera	Kraatz, 1856	Staphylinidae	4	4	s_s	b_w	h_t	f_z	200
Euryusa optabilis	Heer, 1839	Staphylinidae	6	6	s_xs	b_w	h_t	f_z	150
Eusphalerum alpinum	(Heer, 1839)	Staphylinidae	5	4	s_xs	b_w	h_v	f_p	300
Eusphalerum longipenne	(Erichson, 1839)	Staphylinidae	6	5	s_xs	b_w	h_v	f_p	300
Eusphalerum luteum	(Marsham, 1802)	Staphylinidae	7	7	s_xs	b_w	h_v	f_p	200
Eusphalerum minutum	(Fabricius, 1792)	Staphylinidae	7	7	s_xs	b_f	h_v	f_p	300
Eusphalerum pallens	(Heer, 1841)	Staphylinidae	4	4	s_xs	b_o	h_v	f_p	100
Eusphalerum primulae	(Stephens, 1834)	Staphylinidae	5	3	s_xs	b_w	h_v	f_p	100
Eusphalerum rectangulum	(Baudi di Selve, 1870)	Staphylinidae	5	5	s_xs	b_w	h_v	f_p	200
Eusphalerum semicoleoptratum	(Panzer, 1795)	Staphylinidae	5	5	s_s	b_w	h_v	f_p	150
Eusphalerum sorbi	(Gyllenhal, 1810)	Staphylinidae	9	6	s_xs	b_w	h_v	f_p	200
Eutrichapion ervi	(Kirby, 1808)	Brentidae	7	7	s_xs	b_e	h_v	f_p	200
Eutrichapion punctiger	(Paykull, 1792)	Apionidae	7	7	s_xs	b_o	h_v	f_p	200
Eutrichapion viciae	(Paykull, 1800)	Brentidae	14	12	s_xs	b_e	h_v	f_p	300
Evodinus clathratus	(Fabricius, 1792)	Cerambycidae	5	3	s_l	b_w	h_t	f_x	100
Exapion compactum	(Desbrochers des Loges, 1	Brentidae	5	3	s_xs	b_o	h_v	f_p	100
Exapion difficile	(Herbst, 1797)	Brentidae	4	4	s_xs	b_o	h_v	f_p	150
Exapion formaneki	(Wagner, 1929)	Brentidae	7	6	s_xs	b_o	h_v	f_p	200
Exapion fuscirostre	(Fabricius, 1775)	Brentidae	6	5	s_xs	b_o	h_v	f_p	150
Exocentrus adspersus	Mulsant, 1846	Cerambycidae	6	6	s_m	b_w	h_t	f_x	100
Exocentrus lusitanus	(Linné, 1767)	Cerambycidae	5	5	s_s	b_w	h_t	f_x	300
Exocentrus punctipennis	Mulsant & Guillebeau, 185	Cerambycidae	6	6	s_s	b_w	h_t	f_x	>300
Exochomus quadripustulatus	(Linné, 1758)	Coccinellidae	14	13	s_s	b_w	h_v	f_z	300
Exomias araneiformis	(Schrank, 1781)	Curculionidae	4	4	s_s	b_w	h_v	f_p	100
Exomias pellucidus	(Boheman, 1834)	Curculionidae	4	3	s_s	b_e	h_v	f_p	150

Fagniezia impressa	(Panzer, 1803)	Staphylinidae	5	4	s_xs	b_f	h_b	f_z	300
Fissocatops westi	(Krogerus, 1931)	Cholevidae	4	2	s_s	b_w	h_n	f_n	100
Foucartia squamulata	(Herbst, 1795)	Curculionidae	6	6	s_xs	b_o	h_v	f_p	100
Gabrius nigritulus	(Gravenhorst, 1802)	Staphylinidae	4	3	s_s	b_e	h_b	f_z	150
Gabrius osseticus	(Kolenati, 1846)	Staphylinidae	6	4	s_m	b_e	h_b	f_z	200
Gabrius splendidulus	(Gravenhorst, 1802)	Staphylinidae	7	7	s_s	b_w	h_t	f_z	200
Gabrius trossulus	(Nordmann, 1837)	Staphylinidae	6	5	s_s	b_f	h_b	f_z	150
Galeruca pomonae	(Scopoli, 1763)	Chrysomelidae	6	5	s_m	b_o	h_v	f_p	200
Galeruca tanaceti	(Linné, 1758)	Chrysomelidae	9	6	s_m	b_o	h_v	f_p	200
Galerucella aquatica	(Geoffroy, 1785)	Chrysomelidae	6	5	s_s	b_f	h_v	f_p	200
Galerucella nymphaeae	(Linné, 1758)	Chrysomelidae	4	4	s_s	b_f	h_v	f_p	300
Galerucella pusilla	(Duftschmid, 1825)	Chrysomelidae	9	5	s_s	b_f	h_v	f_p	200
Galerucella tenella	(Linné, 1761)	Chrysomelidae	12	10	s_s	b_f	h_v	f_p	200
Gastrophysa polygoni	(Linné, 1758)	Chrysomelidae	9	8	s_s	b_o	h_v	f_p	300
Gastrophysa viridula	(DeGeer, 1775)	Chrysomelidae	5	5	s_s	b_o	h_v	f_p	300
Gauropterus fulgidus	(Fabricius, 1787)	Staphylinidae	4	3	s_m	b_o	h_f	f_z	>300
Gaurotes virginea	(Linné, 1758)	Cerambycidae	6	5	s_m	b_w	h_t	f_x	300
Geodromicus nigrita	(P. W. J. Müller, 1821)	Staphylinidae	4	2	s_s	b_f	h_b	f_z	300
Geotrupes spiniger	(Marsham, 1802)	Geotrupidae	4	4	s_xl	b_o	h_f	f_c	>300
Glischrochilus hortensis	(Geoffroy, 1785)	Nitidulidae	8	7	s_s	b_e	h_f	f_z	300
Glischrochilus quadriguttatus	(Fabricius, 1777)	Nitidulidae	13	12	s_s	b_w	h_t	f_z	300
Glischrochilus quadripunctatus	(Linné, 1758)	Nitidulidae	7	4	s_s	b_w	h_t	f_z	100
Glischrochilus quadrisignatus	(Say, 1835)	Nitidulidae	8	8	s_s	b_e	h_f	f_z	300
Glocianus punctiger	(C. R. Sahlberg, 1835)	Curculionidae	5	4	s_s	b_o	h_v	f_p	300
Gnathotrichus materiarius	(Fitch, 1858)	Curculionidae	5	4	s_s	b_w	h_t	f_m	150
Gonioctena decemnotata	(Marsham, 1802)	Chrysomelidae	4	4	s_m	b_w	h_v	f_p	150
Gonioctena linnaeana	(Schrank, 1781)	Chrysomelidae	7	6	s_m	b_w	h_v	f_p	200
Gonioctena olivacea	(Forster, 1771)	Chrysomelidae	6	5	s_s	b_o	h_v	f_p	>300
Gonioctena quinquepunctata	(Fabricius, 1787)	Chrysomelidae	10	10	s_m	b_w	h_v	f_p	200
Gonioctena viminalis	(Linné, 1758)	Chrysomelidae	9	8	s_m	b_w	h_v	f_p	300
Gonodera luperus	(Herbst, 1783)	Tenebrionidae	10	8	s_m	b_w	h_v	f_x	300
Grammoptera abdominalis	(Stephens, 1831)	Cerambycidae	4	3	s_m	b_w	h_t	f_x	100
Grammoptera ruficornis	(Fabricius, 1781)	Cerambycidae	10	9	s_s	b_w	h_t	f_x	200
Grammoptera ustulata	(Schaller, 1783)	Cerambycidae	5	5	s_m	b_w	h_t	f_x	200
Graptodytes granularis	(Linné, 1767)	Dytiscidae	9	7	s_xs	b_f	h_w	f_z	>300
Grypus equiseti	(Fabricius, 1775)	Curculionidae	9	8	s_s	b_f	h_v	f_p	300
Gyrinus paykulli	G. Ochs, 1927	Gyrinidae	6	5	s_m	b_f	h_w	f_z	300
Gyrinus substriatus	Stephens, 1828	Gyrinidae	8	8	s_m	b_f	h_w	f_z	300
Gyrohypnus angustatus	Stephens, 1833	Staphylinidae	4	4	s_m	b_e	h_f	f_z	100
Gyrohypnus punctulatus	(Paykull, 1789)	Staphylinidae	5	5	s_m	b_e	h_f	f_z	200
Gyrophaena boleti	(Linné, 1758)	Staphylinidae	9	4	s_xs	b_w	h_t	f_m	200
Gyrophaena gentilis	Erichson, 1839	Staphylinidae	4	4	s_xs	b_w	h_p	f_m	300
Gyrophaena joyioides	Wüsthoff, 1937	Staphylinidae	5	4	s_xs	b_e	h_p	f_m	300
Gyrophaena manca	Erichson, 1839	Staphylinidae	4	4	s_xs	b_w	h_t	f_m	300
Gyrophaena munsteri	A. Strand, 1935	Staphylinidae	4	4	s_xs	b_w	h_p	f_m	100
Gyrophaena strictula	Erichson, 1839	Staphylinidae	4	3	s_xs	b_w	h_t	f_m	150

Habrocerus capillaricornis	(Gravenhorst, 1806)	Staphylinidae	11	10	s_s	b_w	h_b	f_z	300
Habroloma nanum	(Paykull, 1799)	Buprestidae	6	4	s_xs	b_o	h_v	f_p	100
Hadrobregmus denticollis	(Creutzer, 1796)	Ptinidae	5	5	s_s	b_w	h_t	f_x	100
Hadrobregmus pertinax	(Linné, 1758)	Anobiidae	4	3	s_s	b_w	h_t	f_x	300
Hadroplontus litura	(Fabricius, 1775)	Curculionidae	6	6	s_s	b_o	h_v	f_p	300
Haliplus laminatus	(Schaller, 1783)	Haliplidae	4	3	s_xs	b_f	h_w	f_p	150
Haliplus lineatocollis	(Marsham, 1802)	Haliplidae	8	8	s_s	b_f	h_w	f_p	300
Haliplus ruficollis	(DeGeer, 1774)	Haliplidae	4	3	s_xs	b_f	h_w	f_p	200
Hallomenus axillaris	(Illiger, 1807)	Tetratomidae	5	3	s_xs	b_w	h_t	f_m	200
Hallomenus binotatus	(Quensel, 1790)	Tetratomidae	6	6	s_s	b_w	h_t	f_m	300
Halyzia sedecimguttata	(Linné, 1758)	Coccinellidae	20	18	s_m	b_w	h_v	f_m	300
Haploglossa marginalis	(Gravenhorst, 1806)	Staphylinidae	6	4	s_s	b_w	h_n	f_z	100
Harmonia axyridis	(Pallas, 1773)	Coccinellidae	7	7	s_s	b_e	h_v	f_z	>300
Harmonia quadripunctata	(Pontoppidan, 1763)	Coccinellidae	4	4	s_s	b_w	h_v	f_z	>300
Harpalus affinis	(Schrank, 1781)	Carabidae	5	5	s_m	b_o	h_b	f_p	200
Harpalus anxius	(Duftschmid, 1812)	Carabidae	7	4	s_m	b_o	h_b	f_p	150
Harpalus atratus	Latreille, 1804	Carabidae	5	5	s_l	b_o	h_b	f_p	300
Harpalus autumnalis	(Duftschmid, 1812)	Carabidae	6	4	s_m	b_o	h_b	f_p	300
Harpalus dimidiatus	(P. Rossi, 1790)	Carabidae	6	6	s_l	b_o	h_b	f_p	100
Harpalus froelichii	J. Sturm, 1818	Carabidae	4	3	s_m	b_o	h_b	f_p	150
Harpalus griseus	(Panzer, 1796)	Carabidae	7	7	s_m	b_o	h_b	f_p	300
Harpalus honestus	(Duftschmid, 1812)	Carabidae	5	4	s_m	b_o	h_b	f_p	100
Harpalus latus	(Linné, 1758)	Carabidae	5	5	s_m	b_o	h_b	f_p	300
Harpalus melancholicus	Dejean, 1829	Carabidae	5	3	s_m	b_o	h_b	f_p	300
Harpalus pumilus	J. Sturm, 1818	Carabidae	5	5	s_s	b_o	h_b	f_p	200
Harpalus rufipes	(DeGeer, 1774)	Carabidae	5	5	s_l	b_o	h_b	f_p	150
Harpalus serripes	(Quensel, 1806)	Carabidae	11	8	s_m	b_o	h_b	f_p	300
Harpalus smaragdinus	(Duftschmid, 1812)	Carabidae	4	3	s_m	b_o	h_b	f_p	100
Harpalus tardus	(Panzer, 1796)	Carabidae	9	9	s_m	b_o	h_b	f_p	300
Helochares lividus	(Forster, 1771)	Hydrophilidae	4	4	s_s	b_f	h_w	f_s	>300
Helochares obscurus	(O. F. Müller, 1776)	Hydrophilidae	10	6	s_s	b_f	h_w	f_s	300
Helophorus brevipalpis	Bedel, 1881	Hydrophilidae	4	4	s_xs	b_f	h_w	f_s	300
Helophorus flavipes	Fabricius, 1792	Hydrophilidae	4	2	s_s	b_f	h_w	f_s	300
Helophorus grandis	Illiger, 1798	Hydrophilidae	5	4	s_m	b_f	h_w	f_s	150
Helophorus minutus	Fabricius, 1775	Hydrophilidae	7	5	s_s	b_f	h_w	f_s	300
Helophorus obscurus	Mulsant, 1844	Hydrophilidae	4	3	s_s	b_f	h_w	f_s	300
Helophorus strigifrons	C. G. Thomson, 1868	Hydrophilidae	4	3	s_s	b_f	h_w	f_s	100
Helops caeruleus	(Linné, 1758)	Tenebrionidae	4	3	s_l	b_w	h_t	f_x	300
Hemicoelus canaliculatus	(C. G. Thomson, 1863)	Ptinidae	6	5	s_s	b_w	h_t	f_x	200
Hemicoelus fulvicornis	(J. Sturm, 1837)	Ptinidae	5	5	s_s	b_w	h_t	f_x	100
Hemicrepidius hirtus	(Herbst, 1784)	Elateridae	11	9	s_l	b_e	h_v	f_p	300
Hemicrepidius niger	(Linné, 1758)	Elateridae	4	4	s_l	b_e	h_v	f_p	300
Hemitrichapion pavidum	(Germar, 1817)	Apionidae	9	8	s_xs	b_o	h_v	f_p	150
Hermaeophaga mercurialis	(Fabricius, 1792)	Chrysomelidae	5	5	s_xs	b_w	h_v	f_p	200
Hesperus rufipennis	(Gravenhorst, 1802)	Staphylinidae	8	4	s_m	b_w	h_t	f_z	100
Heterhelus scutellaris	(Heer, 1841)	Kateretidae	7	6	s_xs	b_w	h_v	f_p	200
Heterhelus solani	(Heer, 1841)	Kateretidae	4	4	s_xs	b_w	h_v	f_p	150

Heterocerus fenestratus	(Thunberg, 1784)	Heteroceridae	11	9	s_s	b_f	h_b	f_p	>300
Heterocerus obsoletus	Curtis, 1828	Heteroceridae	4	2	s_s	b_f	h_b	f_p	100
Heterothops dissimilis	(Gravenhorst, 1802)	Staphylinidae	6	5	s_s	b_e	h_b	f_z	150
Heterothops stiglundbergi	Israelson, 1979	Staphylinidae	4	2	s_s	b_e	h_n	f_z	150
Hippodamia variegata	(Goeze, 1777)	Coccinellidae	6	5	s_s	b_o	h_v	f_z	300
Hippuriphila modeeri	(Linné, 1760)	Chrysomelidae	9	7	s_xs	b_f	h_v	f_p	300
Hispa atra	Linné, 1767	Chrysomelidae	6	6	s_s	b_o	h_v	f_p	300
Hololepta plana	(Sulzer, 1776)	Histeridae	4	4	s_m	b_w	h_t	f_z	150
Holotrichapion pisi	(Fabricius, 1801)	Brentidae	7	6	s_xs	b_e	h_v	f_p	200
Homalota plana	(Gyllenhal, 1810)	Staphylinidae	5	5	s_xs	b_w	h_t	f_z	100
Hoplia argentea	(Poda von Neuhaus, 1761)	Scarabaeidae	7	6	s_m	b_w	h_v	f_p	150
Hoplia philanthus	(Füessly, 1775)	Scarabaeidae	7	5	s_m	b_o	h_v	f_p	300
Hydaticus seminiger	(DeGeer, 1774)	Dytiscidae	6	5	s_l	b_f	h_w	f_z	300
Hydaticus transversalis	(Pontoppidan, 1763)	Dytiscidae	4	4	s_l	b_f	h_w	f_z	300
Hydraena assimilis	Rey, 1885	Hydraenidae	4	4	s_xs	b_f	h_w	f_s	300
Hydraena britteni	Joy, 1907	Hydraenidae	5	3	s_xs	b_f	h_w	f_s	200
Hydraena gracilis	Germar, 1824	Hydraenidae	6	5	s_xs	b_f	h_w	f_s	>300
Hydraena lapidicola	Kiesenwetter, 1849	Hydraenidae	5	4	s_xs	b_f	h_w	f_s	100
Hydraena melas	Dalla Torre, 1877	Hydraenidae	4	4	s_xs	b_f	h_w	f_s	300
Hydraena minutissima	Stephens, 1829	Hydraenidae	6	6	s_xs	b_f	h_w	f_s	200
Hydraena palustris	Erichson, 1837	Hydraenidae	7	7	s_xs	b_f	h_w	f_s	300
Hydrobius fuscipes	(Linné, 1758)	Hydrophilidae	23	21	s_m	b_f	h_w	f_s	>300
Hydrochara caraboides	(Linné, 1758)	Hydrophilidae	6	6	s_l	b_f	h_w	f_s	200
Hydroporus angustatus	J. Sturm, 1835	Dytiscidae	4	4	s_s	b_f	h_w	f_z	300
Hydroporus erythrocephalus	(Linné, 1758)	Dytiscidae	5	4	s_s	b_f	h_w	f_z	300
Hydroporus incognitus	Sharp, 1869	Dytiscidae	4	3	s_s	b_f	h_w	f_z	200
Hydroporus memnonius	Nicolai, 1822	Dytiscidae	7	6	s_s	b_f	h_w	f_z	300
Hydroporus palustris	(Linné, 1761)	Dytiscidae	10	7	s_s	b_f	h_w	f_z	300
Hydroporus planus	(Fabricius, 1781)	Dytiscidae	10	9	s_s	b_f	h_w	f_z	>300
Hydroporus scalesianus	(Stephens, 1828)	Dytiscidae	4	2	s_xs	b_f	h_w	f_z	150
Hydroporus striola	(Gyllenhal, 1826)	Dytiscidae	4	3	s_s	b_f	h_w	f_z	300
Hygrotus decoratus	(Gyllenhal, 1810)	Dytiscidae	6	5	s_xs	b_f	h_w	f_z	300
Hygrotus inaequalis	(Fabricius, 1777)	Dytiscidae	7	7	s_s	b_f	h_w	f_z	>300
Hygrotus parallellogrammus	(Ahrens, 1812)	Dytiscidae	4	3	s_s	b_f	h_w	f_z	300
Hygrotus versicolor	(Schaller, 1783)	Dytiscidae	4	3	s_s	b_f	h_w	f_z	200
Hylastes ater	(Paykull, 1800)	Curculionidae	5	3	s_s	b_w	h_t	f_x	150
Hylastes cunicularius	Erichson, 1836	Scolytidae	4	3	s_s	b_w	h_t	f_x	100
Hylobius abietis	(Linné, 1758)	Curculionidae	9	9	s_m	b_w	h_t	f_x	>300
Hylurgops palliatus	(Gyllenhal, 1813)	Scolytidae	9	7	s_xs	b_w	h_t	f_x	200
Hymenalia rufipes	(Fabricius, 1792)	Tenebrionidae	6	5	s_m	b_w	h_t	f_x	300
Hypera meles	(Fabricius, 1792)	Curculionidae	5	5	s_s	b_o	h_v	f_p	300
Hypera miles	(Paykull, 1792)	Curculionidae	10	9	s_s	b_e	h_v	f_p	300
Hypera nigrirostris	(Fabricius, 1775)	Curculionidae	12	12	s_s	b_o	h_v	f_p	300
Hypera postica	(Gyllenhal, 1813)	Curculionidae	7	7	s_s	b_e	h_v	f_p	200
Hyperisus plumbeum	(Illiger, 1801)	Ptinidae	6	6	s_s	b_w	h_t	f_x	200
Hyphydrus ovatus	(Linné, 1761)	Dytiscidae	5	4	s_s	b_f	h_w	f_z	300

Hypnogyra angularis	(Ganglbauer, 1895)	Staphylinidae	5	5	s_m	b_w	h_t	f_z	200
Hypnoidus riparius	(Fabricius, 1792)	Elateridae	5	5	s_m	b_f	h_b	f_e	300
Hypocassida subferruginea	(Schrank, 1776)	Chrysomelidae	4	3	s_s	b_o	h_v	f_p	>300
Hypoganus inunctus	(Lacordaire, 1835)	Elateridae	4	4	s_m	b_w	h_t	f_x	150
Iberodorcadion fuliginator	(Linné, 1758)	Cerambycidae	5	5	s_l	b_o	h_b	f_p	150
Idolus picipennis	(Bach, 1852)	Elateridae	5	5	s_s	b_o	h_v	f_e	150
Ilybius ater	(DeGeer, 1774)	Dytiscidae	6	6	s_l	b_f	h_w	f_z	300
Ilybius fenestratus	(Fabricius, 1781)	Dytiscidae	6	4	s_l	b_f	h_w	f_z	200
Ilybius fuliginosus	(Fabricius, 1792)	Dytiscidae	9	9	s_m	b_f	h_w	f_z	300
Ilybius neglectus	Erichson, 1837	Dytiscidae	7	4	s_m	b_f	h_w	f_z	200
Ilybius quadriguttatus	(Lacordaire, 1835)	Dytiscidae	5	4	s_l	b_f	h_w	f_z	200
Ilybius subtilis	Erichson, 1837	Dytiscidae	9	4	s_m	b_f	h_w	f_z	300
Involvulus caeruleus	(DeGeer, 1775)	Attelabidae	5	4	s_s	b_w	h_v	f_p	100
Involvulus cupreus	(Linné, 1758)	Attelabidae	4	4	s_s	b_w	h_v	f_p	300
Ips typographus	(Linné, 1758)	Scolytidae	5	3	s_s	b_w	h_t	f_x	100
Ischnomera cyanea	(Fabricius, 1792)	Oedemeridae	4	4	s_m	b_w	h_t	f_x	100
Ischnopterapion loti	(Kirby, 1808)	Apionidae	7	7	s_xs	b_o	h_v	f_p	150
Ischnopterapion virens	(Herbst, 1797)	Brentidae	6	5	s_xs	b_e	h_v	f_p	200
Ischnosoma splendidum	(Gravenhorst, 1806)	Staphylinidae	8	7	s_s	b_e	h_b	f_z	150
Isomira murina	(Linné, 1758)	Tenebrionidae	9	7	s_m	b_o	h_v	f_p	300
Kalcapion semivittatum	(Gyllenhal, 1833)	Brentidae	6	3	s_xs	b_o	h_v	f_p	200
Kateretes pedicularius	(Linné, 1758)	Kateretidae	12	9	s_xs	b_f	h_v	f_p	300
Kyklioacalles roboris	(Curtis, 1834)	Curculionidae	7	4	s_s	b_w	h_t	f_x	100
Labidostomis longimana	(Linné, 1760)	Chrysomelidae	10	8	s_s	b_o	h_v	f_p	300
Laccobius bipunctatus	(Fabricius, 1775)	Hydrophilidae	9	7	s_s	b_f	h_w	f_s	300
Laccobius minutus	(Linné, 1758)	Hydrophilidae	7	6	s_xs	b_f	h_w	f_s	300
Laccobius striatulus	(Fabricius, 1801)	Hydrophilidae	8	6	s_s	b_f	h_w	f_s	300
Laccophilus hyalinus	(DeGeer, 1774)	Dytiscidae	4	3	s_s	b_f	h_w	f_z	200
Laccophilus minutus	(Linné, 1758)	Dytiscidae	4	3	s_s	b_f	h_w	f_z	150
Lagria atripes	Mulsant & Guillebeau, 185	Tenebrionidae	6	4	s_l	b_o	h_v	f_p	200
Lagria hirta	(Linné, 1758)	Lagriidae	13	12	s_m	b_e	h_v	f_p	300
Lampyris noctiluca	(Linné, 1767)	Lampyridae	6	6	s_l	b_w	h_b	f_z	300
Laricobius erichsonii	Rosenhauer, 1846	Derodontidae	5	5	s_xs	b_w	h_v	f_z	200
Larinus iaceae	(Fabricius, 1775)	Curculionidae	4	3	s_m	b_o	h_v	f_p	200
Larinus planus	(Fabricius, 1792)	Curculionidae	8	7	s_m	b_o	h_v	f_p	300
Larinus sturnus	(Schaller, 1783)	Curculionidae	8	6	s_m	b_o	h_v	f_p	300
Larinus turbinatus	Gyllenhal, 1835	Curculionidae	11	11	s_m	b_o	h_v	f_p	200
Lasiorhynchites olivaceus	(Gyllenhal, 1833)	Attelabidae	5	4	s_s	b_w	h_v	f_p	300
Lathrobium brunnipes	(Fabricius, 1792)	Staphylinidae	5	5	s_m	b_f	h_b	f_z	200
Lathrobium fovulum	Stephens, 1833	Staphylinidae	7	7	s_m	b_f	h_b	f_z	300
Lathrobium fulvipenne	(Gravenhorst, 1806)	Staphylinidae	9	7	s_m	b_e	h_b	f_z	300
Lathrobium longulum	Gravenhorst, 1802	Staphylinidae	7	6	s_s	b_f	h_b	f_z	200
Latridius minutus	(Linné, 1767)	Latridiidae	4	4	s_xs	b_e	h_f	f_m	300
Lebia cruxminor	(Linné, 1758)	Carabidae	6	5	s_m	b_o	h_v	f_z	150
Leiodes polita	(Marsham, 1802)	Leiodidae	4	4	s_xs	b_e	h_p	f_m	100
Leiopus linnei	Wallin, Nylander & Kvamme	Cerambycidae	5	5	s_m	b_w	h_t	f_x	300

Leistus ferrugineus	(Linné, 1758)	Carabidae	6	6	s_m	b_e	h_b	f_z	200
Leistus spinibarbis	(Fabricius, 1775)	Carabidae	4	4	s_m	b_o	h_b	f_z	300
Leptacinus pusillus	(Stephens, 1833)	Staphylinidae	4	3	s_s	b_e	h_f	f_z	150
Leptacinus sulcifrons	(Stephens, 1833)	Staphylinidae	7	6	s_s	b_o	h_f	f_z	100
Leptinotarsa decemlineata	(Say, 1824)	Chrysomelidae	5	5	s_m	b_o	h_v	f_p	>300
Leptinus testaceus	P. W. J. Müller, 1817	Leiodidae	6	6	s_xs	b_e	h_n	f_z	200
Leptura quadrifasciata	(Linné, 1758)	Cerambycidae	5	4	s_l	b_w	h_t	f_x	300
Lepturobosca virens	(Linné, 1758)	Cerambycidae	5	3	s_l	b_w	h_t	f_x	150
Leptusa fumida	(Erichson, 1839)	Staphylinidae	7	7	s_xs	b_w	h_t	f_z	150
Leptusa pulchella	(Mannerheim, 1830)	Staphylinidae	9	9	s_xs	b_w	h_t	f_z	300
Lepyrus capucinus	(Schaller, 1783)	Curculionidae	4	3	s_m	b_w	h_v	f_p	100
Lepyrus palustris	(Scopoli, 1763)	Curculionidae	4	4	s_m	b_w	h_v	f_p	200
Lesteva longoelytrata	(Goeze, 1777)	Staphylinidae	5	5	s_s	b_f	h_b	f_z	300
Lesteva punctata	Erichson, 1839	Staphylinidae	5	4	s_s	b_f	h_b	f_z	100
Lignyodes enucleator	(Panzer, 1798)	Curculionidae	5	5	s_s	b_w	h_v	f_p	300
Lilioceris lilii	(Scopoli, 1763)	Chrysomelidae	5	4	s_m	b_o	h_v	f_p	200
Lilioceris merdigera	(Linné, 1758)	Chrysomelidae	6	5	s_m	b_w	h_v	f_p	200
Limnebius truncatellus	(Thunberg, 1794)	Hydraenidae	4	4	s_xs	b_f	h_w	f_s	>300
Limnichus sericeus	(Duftschmidt, 1825)	Limnichidae	6	5	s_xs	b_f	h_b	f_p	300
Limnius opacus	P. W. J. Müller, 1806	Elmidae	5	5	s_xs	b_f	h_w	f_p	150
Limnius volckmari	(Panzer, 1793)	Elmidae	8	8	s_s	b_f	h_w	f_p	300
Limnobaris dolorosa	(Goeze, 1777)	Curculionidae	8	7	s_s	b_f	h_v	f_p	150
Limnobaris t-album	(Linné, 1758)	Curculionidae	4	3	s_s	b_f	h_v	f_p	100
Limobius borealis	(Paykull, 1792)	Curculionidae	6	6	s_xs	b_o	h_v	f_p	150
Limodromus assimilis	(Paykull, 1790)	Carabidae	15	13	s_l	b_w	h_b	f_z	>300
Limonius minutus	(Linné, 1758)	Elateridae	4	4	s_m	b_w	h_v	f_p	300
Limonius poneli	Leseigneur & Mertlik, 200	Elateridae	6	6	s_m	b_o	h_v	f_p	200
Liocyrtusa minuta	(Ahrens, 1812)	Leiodidae	7	6	s_xs	b_e	h_p	f_m	100
Liodopria serricornis	(Gyllenhal, 1813)	Leiodidae	5	5	s_xs	b_w	h_t	f_m	300
Liogluta longiuscula	(Gravenhorst, 1802)	Staphylinidae	9	8	s_s	b_e	h_b	f_z	200
Lionychus quadrillum	(Duftschmid, 1812)	Carabidae	4	3	s_s	b_f	h_b	f_z	200
Liophloeus tessulatus	(O. F. Müller, 1776)	Curculionidae	5	5	s_m	b_o	h_v	f_p	150
Lissodema cursor	(Gyllenhal, 1813)	Salpingidae	5	5	s_s	b_w	h_t	f_z	150
Litargus balteatus	LeConte, 1856	Mycetophagidae	4	4	s_xs	b_w	h_t	f_m	150
Litargus connexus	(Geoffroy, 1785)	Mycetophagidae	13	12	s_xs	b_w	h_t	f_m	300
Lithocharis nigriceps	(Kraatz, 1859)	Staphylinidae	4	4	s_s	b_e	h_f	f_z	200
Lixus iridis	A. G. Olivier, 1807	Curculionidae	8	6	s_l	b_f	h_v	f_p	200
Lixus pulverulentus	(Scopoli, 1763)	Curculionidae	4	3	s_l	b_o	h_v	f_p	100
Lobrathium multipunctum	Gravenhorst, 1802	Staphylinidae	4	4	s_m	b_f	h_b	f_z	>300
Lochmaea caprea	(Linné, 1758)	Chrysomelidae	10	10	s_s	b_e	h_v	f_p	300
Lochmaea crataegi	(Forster, 1771)	Chrysomelidae	10	8	s_s	b_w	h_v	f_p	200
Longitarsus anchusae	(Paykull, 1799)	Chrysomelidae	10	7	s_xs	b_e	h_v	f_p	150
Longitarsus atricillus	(Linné, 1760)	Chrysomelidae	6	6	s_xs	b_o	h_v	f_p	150
Longitarsus ballotae	(Marsham, 1802)	Chrysomelidae	6	4	s_xs	b_o	h_v	f_p	150
Longitarsus brunneus	(Duftschmid, 1825)	Chrysomelidae	5	4	s_xs	b_f	h_v	f_p	200
Longitarsus echii	(Koch, 1803)	Chrysomelidae	4	3	s_s	b_o	h_v	f_p	100
Longitarsus exsoletus	(Linné, 1758)	Chrysomelidae	7	5	s_xs	b_o	h_v	f_p	200

Longitarsus helvolus	Kutschera, 1863	Chrysomelidae	4	3	s_xs	b_o	h_v	f_p	100
Longitarsus jacobaeae	(Waterhouse, 1858)	Chrysomelidae	4	4	s_xs	b_o	h_v	f_p	100
Longitarsus luridus	(Scopoli, 1763)	Chrysomelidae	8	8	s_xs	b_e	h_v	f_p	300
Longitarsus melanocephalus	(DeGeer, 1775)	Chrysomelidae	5	5	s_xs	b_e	h_v	f_p	200
Longitarsus minusculus	(Foudras, 1860)	Chrysomelidae	4	2	s_xs	b_o	h_v	f_p	100
Longitarsus nigerrimus	(Gyllenhal, 1827)	Chrysomelidae	5	4	s_xs	b_f	h_v	f_p	100
Longitarsus obliteratus	(Rosenhauer, 1847)	Chrysomelidae	12	9	s_xs	b_o	h_v	f_p	>300
Longitarsus ochroleucus	(Marsham, 1802)	Chrysomelidae	5	4	s_xs	b_o	h_v	f_p	100
Longitarsus parvulus	(Paykull, 1799)	Chrysomelidae	5	5	s_xs	b_o	h_v	f_p	>300
Longitarsus pratensis	(Panzer, 1794)	Chrysomelidae	4	4	s_xs	b_o	h_v	f_p	200
Longitarsus reichei	(Allard, 1860)	Chrysomelidae	4	3	s_xs	b_f	h_v	f_p	100
Longitarsus rubiginosus	(Foudras, 1860)	Chrysomelidae	4	4	s_xs	b_e	h_v	f_p	200
Longitarsus tabidus	(Fabricius, 1775)	Chrysomelidae	7	6	s_s	b_o	h_v	f_p	>300
Lordithon lunulatus	(Linné, 1760)	Staphylinidae	4	4	s_m	b_w	h_p	f_z	200
Lordithon thoracicus	(Fabricius, 1777)	Staphylinidae	6	6	s_s	b_w	h_p	f_z	300
Lordithon trinotatus	(Erichson, 1839)	Staphylinidae	9	8	s_s	b_w	h_p	f_z	300
Loricera pilicornis	(Fabricius, 1775)	Carabidae	10	7	s_m	b_e	h_b	f_z	300
Lucanus cervus	(Linné, 1758)	Lucanidae	7	6	s_xl	b_w	h_t	f_x	150
Luperus longicornis	(Fabricius, 1781)	Chrysomelidae	7	7	s_s	b_o	h_v	f_p	300
Luperus luperus	(Sulzer, 1776)	Chrysomelidae	7	6	s_s	b_w	h_v	f_p	300
Luperus viridipennis	Germar, 1824	Chrysomelidae	6	6	s_s	b_f	h_v	f_p	150
Lygistopterus sanguineus	(Linné, 1758)	Lycidae	13	10	s_m	b_w	h_t	f_z	>300
Lymexylon navale	(Linné, 1758)	Lymexylidae	8	6	s_l	b_w	h_t	f_x	150
Lythraria salicariae	(Paykull, 1800)	Chrysomelidae	9	7	s_xs	b_f	h_v	f_p	200
Magdalis armigera	(Geoffroy, 1785)	Curculionidae	4	4	s_s	b_w	h_t	f_x	300
Magdalis duplicata	Germar, 1819	Curculionidae	7	5	s_s	b_w	h_t	f_x	300
Magdalis flavicornis	(Gyllenhal, 1836)	Curculionidae	5	5	s_s	b_w	h_t	f_x	200
Magdalis ruficornis	(Linné, 1758)	Curculionidae	11	9	s_s	b_w	h_t	f_x	200
Magdalis violacea	(Linné, 1758)	Curculionidae	8	6	s_s	b_w	h_t	f_x	200
Malachius bipustulatus	(Linné, 1758)	Melyridae	23	20	s_s	b_w	h_t	f_z	>300
Maladera holosericea	(Scopoli, 1772)	Scarabaeidae	5	3	s_m	b_o	h_b	f_p	300
Malthinus biguttatus	(Linné, 1758)	Cantharidae	7	4	s_s	b_w	h_t	f_z	300
Malthinus flaveolus	(Herbst, 1786)	Cantharidae	4	4	s_s	b_w	h_t	f_z	150
Malthinus frontalis	(Marsham, 1802)	Cantharidae	4	4	s_s	b_w	h_t	f_z	200
Malthinus seriepunctatus	Kiesenwetter, 1852	Cantharidae	5	5	s_s	b_w	h_t	f_z	100
Malthodes brevicollis	(Paykull, 1798)	Cantharidae	4	4	s_xs	b_w	h_t	f_z	300
Malthodes crassicornis	(Maeklin, 1846)	Cantharidae	4	2	s_xs	b_w	h_t	f_z	150
Malthodes fuscus	(Waltl, 1838)	Cantharidae	5	4	s_s	b_w	h_t	f_z	150
Malthodes guttifer	Kiesenwetter, 1852	Cantharidae	6	5	s_s	b_w	h_t	f_z	200
Malthodes hexacanthus	Kiesenwetter, 1852	Cantharidae	5	3	s_xs	b_w	h_t	f_z	200
Malthodes maurus	(Laporte, 1840)	Cantharidae	4	4	s_s	b_w	h_t	f_z	300
Malthodes minimus	(Linné, 1758)	Cantharidae	8	6	s_s	b_w	h_t	f_z	100
Malthodes mysticus	Kiesenwetter, 1852	Cantharidae	5	4	s_s	b_w	h_t	f_z	200
Malthodes pumilus	(Brébisson, 1835)	Cantharidae	5	3	s_xs	b_w	h_t	f_z	150
Malthodes spathifer	Kiesenwetter, 1852	Cantharidae	4	4	s_s	b_w	h_t	f_z	150
Malvapion malvae	(Fabricius, 1775)	Brentidae	9	6	s_xs	b_o	h_v	f_p	150
Mantura chrysanthemi	(Koch, 1803)	Chrysomelidae	7	4	s_xs	b_o	h_v	f_p	300

Mantura obtusata	(Gyllenhal, 1813)	Chrysomelidae	4	2	s_xs	b_f	h_v	f_p	150
Margarinotus striola	(C. R. Sahlberg, 1819)	Histeridae	4	3	s_m	b_w	h_f	f_z	150
Mecinus pascuorum	(Gyllenhal, 1813)	Curculionidae	8	7	s_xs	b_o	h_v	f_p	100
Mecinus pyraster	(Herbst, 1795)	Curculionidae	4	4	s_s	b_o	h_v	f_p	150
Medon apicalis	(Kraatz, 1857)	Staphylinidae	7	6	s_s	b_e	h_b	f_z	300
Medon brunneus	(Erichson, 1839)	Staphylinidae	5	5	s_s	b_w	h_b	f_z	150
Medon ripicola	(Kraatz, 1854)	Staphylinidae	6	5	s_s	b_f	h_b	f_z	100
Megarthrus denticollis	(L. Beck, 1817)	Staphylinidae	6	6	s_xs	b_e	h_f	f_s	300
Megasternum concinnum	(Marsham, 1802)	Hydrophilidae	5	5	s_xs	b_e	h_f	f_s	300
Melanophthalma transversalis	(Gyllenhal, 1827)	Latridiidae	10	9	s_xs	b_e	h_b	f_m	100
Melanotus brunnipes	(Germar, 1824)	Elateridae	13	5	s_l	b_w	h_v	f_e	200
Melanotus castanipes	(Paykull, 1800)	Elateridae	7	6	s_l	b_w	h_t	f_x	300
Melanotus crassicollis	(Erichson, 1841)	Elateridae	4	2	s_l	b_o	h_t	f_x	100
Melanotus villosus	(Geoffroy, 1785)	Elateridae	8	8	s_l	b_w	h_t	f_x	300
Meligethes aeneus	(Fabricius, 1775)	Nitidulidae	12	11	s_xs	b_e	h_v	f_p	300
Meligethes anthracinus	C. N. F. Brisout de Barne	Nitidulidae	4	3	s_xs	b_o	h_v	f_p	100
Meligethes brachialis	Erichson, 1845	Nitidulidae	5	4	s_xs	b_o	h_v	f_p	100
Meligethes brunnicornis	J. Sturm, 1845	Nitidulidae	5	4	s_xs	b_w	h_v	f_p	100
Meligethes denticulatus	(Heer, 1841)	Nitidulidae	6	6	s_xs	b_e	h_v	f_p	300
Meligethes flavimanus	Stephens, 1830	Nitidulidae	4	4	s_s	b_o	h_v	f_p	150
Meligethes haemorrhoidalis	Förster, 1849	Nitidulidae	4	2	s_xs	b_w	h_v	f_p	100
Meligethes lugubris	J. Sturm, 1845	Nitidulidae	6	5	s_xs	b_o	h_v	f_p	150
Meligethes ovatus	J. Sturm, 1845	Nitidulidae	4	4	s_xs	b_e	h_v	f_p	100
Meligethes serripes	(Gyllenhal, 1827)	Nitidulidae	4	3	s_xs	b_o	h_v	f_p	100
Meligethes solidus	(Kugelann, 1794)	Nitidulidae	7	3	s_xs	b_o	h_v	f_p	150
Meligethes subrugosus	(Gyllenhal, 1808)	Nitidulidae	5	5	s_xs	b_o	h_v	f_p	200
Meligethes sulcatus	C. N. F. Brisout de Barne	Nitidulidae	4	2	s_xs	b_o	h_v	f_p	150
Meligethes symphyti	(Heer, 1841)	Nitidulidae	4	4	s_xs	b_f	h_v	f_p	200
Meligethes viridescens	(Fabricius, 1787)	Nitidulidae	5	5	s_xs	b_e	h_v	f_p	300
Meloe proscarabaeus	Linné, 1758	Meloidae	4	3	s_xl	b_o	h_n	f_z	100
Meloe violaceus	Marsham, 1802	Meloidae	5	4	s_xl	b_w	h_n	f_z	300
Meotica exilis	(Gravenhorst, 1806)	Staphylinidae	4	4	s_xs	b_f	h_b	f_z	150
Mesocoelopus niger	(P. W. J. Müller, 1821)	Anobiidae	6	5	s_s	b_w	h_t	f_x	150
Mesosa nebulosa	(Fabricius, 1781)	Cerambycidae	5	5	s_l	b_w	h_t	f_x	150
Metacantharis clypeata	(Illiger, 1798)	Cantharidae	6	4	s_m	b_o	h_v	f_z	300
Metacantharis discoidea	(Ahrens, 1812)	Cantharidae	5	5	s_m	b_w	h_v	f_z	300
Miarus ajugae	(Herbst, 1795)	Curculionidae	7	6	s_s	b_o	h_v	f_p	300
Micrambe abietis	(Paykull, 1798)	Cryptophagidae	4	3	s_xs	b_w	h_t	f_m	100
Micrambe pilosula	(Erichson, 1846)	Cryptophagidae	5	5	s_xs	b_w	h_v	f_m	100
Micrelus ericae	(Gyllenhal, 1813)	Curculionidae	6	5	s_xs	b_o	h_v	f_p	300
Microcara testacea	(Linné, 1767)	Scirtidae	6	5	s_s	b_f	h_w	f_s	200
Microlestes maurus	(J. Sturm, 1827)	Carabidae	5	4	s_xs	b_o	h_b	f_z	150
Micropeplus fulvus	Erichson, 1840	Staphylinidae	7	7	s_xs	b_e	h_f	f_m	200
Microscydmus minimus	(Chaudoir, 1845)	Scydmaenidae	4	3	s_xs	b_w	h_t	f_z	150
Mogulones asperifoliarum	(Gyllenhal, 1813)	Curculionidae	13	8	s_xs	b_e	h_v	f_p	150
Mogulones crucifer	(Pallas, 1771)	Curculionidae	9	6	s_s	b_o	h_v	f_p	100

Mogulones geographicus	(Goeze, 1777)	Curculionidae	12	8	s_s	b_o	h_v	f_p	150
Molops piceus	(Panzer, 1793)	Carabidae	6	6	s_l	b_w	h_b	f_z	200
Molorchus minor	(Linné, 1758)	Cerambycidae	7	7	s_l	b_w	h_t	f_x	200
Monochamus sartor	(Fabricius, 1787)	Cerambycidae	8	5	s_xl	b_w	h_t	f_x	150
Mononychus punctumalbum	(Herbst, 1784)	Curculionidae	6	5	s_s	b_f	h_v	f_p	200
Monotoma longicollis	(Gyllenhal, V SZ)	Monotomidae	13	9	s_xs	b_e	h_f	f_m	300
Monotoma picipes	Herbst, 1793	Monotomidae	13	10	s_xs	b_e	h_f	f_m	300
Mordella aculeata	Linné, 1758	Mordellidae	4	4	s_m	b_w	h_t	f_x	100
Mordella brachyura	Mulsant, 1856	Mordellidae	6	5	s_m	b_w	h_t	f_x	200
Mordella holomelaena	Apfelbeck, 1914	Mordellidae	5	5	s_m	b_w	h_t	f_x	200
Mordellistena neuwaldeggiana	(Panzer, 1796)	Mordellidae	4	4	s_s	b_w	h_t	f_x	150
Mordellistena pseudobrevicauda	Ermisch, 1963	Mordellidae	5	4	s_s	b_o	h_v	f_p	200
Mordellistena variegata	(Fabricius, 1798)	Mordellidae	4	4	s_s	b_w	h_t	f_x	150
Mordellochroa abdominalis	(Fabricius, 1775)	Mordellidae	7	7	s_s	b_w	h_t	f_x	300
Mycetaea subterranea	(Fabricius, 1801)	Endomychidae	4	4	s_xs	b_e	h_f	f_m	300
Mycetina cruciata	(Schaller, 1783)	Endomychidae	5	3	s_s	b_w	h_t	f_m	100
Mycetochara maura	(Fabricius, 1792)	Tenebrionidae	5	3	s_s	b_w	h_t	f_x	200
Mycetophagus atomarius	(Fabricius, 1787)	Mycetophagidae	6	5	s_s	b_w	h_t	f_m	300
Mycetophagus multipunctatus	Fabricius, 1792	Mycetophagidae	4	4	s_s	b_w	h_t	f_m	200
Mycetophagus piceus	(Fabricius, 1777)	Mycetophagidae	8	8	s_s	b_w	h_t	f_m	300
Mycetophagus quadripustulatus	(Linné, 1760)	Mycetophagidae	5	5	s_s	b_w	h_t	f_m	200
Myllaena brevicornis	(A. [H.] Matthews, 1838)	Staphylinidae	5	4	s_xs	b_f	h_b	f_z	300
Myllaena dubia	(Gravenhorst, 1806)	Staphylinidae	7	6	s_xs	b_f	h_b	f_z	300
Myllaena intermedia	Erichson, 1837	Staphylinidae	9	8	s_xs	b_f	h_b	f_z	200
Myllaena minuta	(Gravenhorst, 1806)	Staphylinidae	5	4	s_xs	b_f	h_b	f_z	200
Myrmechixenus subterraneus	Chevrolat, 1835	Tenebrionidae	5	3	s_xs	b_o	h_n	f_z	150
Myrmecocephalus concinnus	(Erichson, 1839)	Staphylinidae	6	6	s_xs	b_o	h_f	f_z	150
Myrrha octodecimguttata	(Linné, 1758)	Coccinellidae	9	7	s_s	b_w	h_v	f_z	200
Myzia oblongoguttata	(Linné, 1758)	Coccinellidae	4	4	s_m	b_w	h_v	f_z	150
Nalassus laevioctostriatus	(Goeze, 1777)	Tenebrionidae	6	6	s_m	b_w	h_b	f_s	300
Nanophyes brevis	Boheman, 1845	Brentidae	5	3	s_xs	b_f	h_v	f_p	100
Nargus anisotomoides	(Spence, 1815)	Leiodidae	8	7	s_xs	b_w	h_b	f_n	200
Nargus wilkini	(Spence, 1815)	Leiodidae	6	5	s_xs	b_w	h_b	f_n	200
Nebria brevicollis	(Fabricius, 1792)	Carabidae	13	11	s_l	b_w	h_b	f_z	300
Nebria hellwigii	(Panzer, 1803)	Carabidae	6	4	s_l	b_f	h_b	f_z	100
Nebria picicornis	(Fabricius, 1801)	Carabidae	4	4	s_l	b_f	h_b	f_z	300
Nebria rufescens	(Ström, 1768)	Carabidae	8	6	s_m	b_f	h_b	f_z	200
Nebria salina	Fairmaire & Laboulbène, 1	Carabidae	6	6	s_m	b_o	h_b	f_z	200
Necrobia violacea	(Linné, 1758)	Cleridae	12	9	s_s	b_o	h_f	f_z	300
Necrodes littoralis	(Linné, 1758)	Silphidae	8	6	s_l	b_e	h_f	f_n	300
Necrophilus subterraneus	(Dahl, 1807)	Agyrtidae	4	2	s_m	b_w	h_b	f_z	200
Nedyus quadrimaculatus	(Linné, 1758)	Curculionidae	13	13	s_xs	b_e	h_v	f_p	300
Nemadus colonoides	(Kraatz, 1851)	Leiodidae	5	4	s_xs	b_w	h_t	f_n	150
Nemozoma elongatum	(Linné, 1761)	Trogositidae	5	5	s_s	b_w	h_t	f_z	200
Neobisnius procerulus	(Gravenhorst, 1806)	Staphylinidae	4	3	s_s	b_f	h_b	f_z	300

Neocoenorrhinus germanicus	(Herbst, 1797)	Rhynchitidae	9	9	s_xs	b_e	h_v	f_p	200
Neocoenorrhinus pauxillus	(Germar, 1824)	Attelabidae	8	7	s_xs	b_w	h_v	f_p	150
Neocrepidodera femorata	(Gyllenhal, 1813)	Chrysomelidae	12	10	s_s	b_f	h_v	f_p	300
Neocrepidodera ferruginea	(Scopoli, 1763)	Chrysomelidae	11	10	s_s	b_o	h_v	f_p	300
Neocrepidodera melanostoma	(L. Redtenbacher, 1849)	Chrysomelidae	5	3	s_xs	b_o	h_v	f_p	100
Neocrepidodera peirolerii	(Kutschera, 1860)	Chrysomelidae	8	7	s_s	b_o	h_v	f_p	100
Neocrepidodera transversa	(Marsham, 1802)	Chrysomelidae	10	10	s_s	b_o	h_v	f_p	300
Nephus quadrimaculatus	(Herbst, 1783)	Coccinellidae	6	6	s_xs	b_w	h_v	f_z	300
Nephus redtenbacheri	(Mulsant, 1846)	Coccinellidae	7	6	s_xs	b_f	h_v	f_z	300
Nicrophorus humator	(Gleditsch, 1767)	Silphidae	4	4	s_xl	b_e	h_f	f_n	100
Nicrophorus interruptus	Stephens, 1830	Silphidae	7	5	s_l	b_o	h_f	f_n	150
Nicrophorus investigator	Zetterstedt, 1824	Silphidae	10	8	s_l	b_w	h_f	f_n	300
Nicrophorus vespillo	(Linné, 1758)	Silphidae	10	10	s_l	b_o	h_f	f_n	300
Nicrophorus vespilloides	Herbst, 1783	Silphidae	10	10	s_l	b_w	h_f	f_n	300
Nitidula bipunctata	(Linné, 1758)	Nitidulidae	4	3	s_s	b_e	h_f	f_z	100
Nossidium pilosellum	(Marsham, 1802)	Ptiliidae	7	7	s_xs	b_w	h_t	f_m	300
Notaris scirpi	(Fabricius, 1792)	Brachyceridae	4	4	s_m	b_f	h_v	f_p	200
Noterus crassicornis	(O. F. Müller, 1776)	Noteridae	8	5	s_s	b_f	h_w	f_z	200
Nothodes parvulus	(Panzer, 1799)	Elateridae	5	5	s_m	b_o	h_v	f_p	100
Notiophilus aestuans	Dejean, 1826	Carabidae	4	4	s_s	b_o	h_b	f_z	200
Notiophilus aquaticus	(Linné, 1758)	Carabidae	7	6	s_s	b_f	h_b	f_z	300
Notiophilus germinyi	Fauvel, 1863	Carabidae	5	5	s_s	b_o	h_b	f_z	200
Notiophilus palustris	(Duftschmid, 1812)	Carabidae	8	8	s_s	b_e	h_b	f_z	300
Notoxus monoceros	(Linné, 1760)	Anthicidae	10	9	s_s	b_o	h_b	f_z	300
Oberea oculata	(Linné, 1758)	Cerambycidae	5	4	s_l	b_w	h_t	f_x	300
Ocalea picata	(Stephens, 1832)	Staphylinidae	6	6	s_s	b_f	h_b	f_z	300
Ochina ptinoides	(Marsham, 1802)	Ptinidae	4	2	s_s	b_w	h_t	f_x	100
Ochthebius exsculptus	Germar, 1824	Hydraenidae	4	4	s_xs	b_f	h_w	f_s	200
Ochthebius marinus	(Paykull, 1798)	Hydraenidae	5	3	s_xs	b_f	h_w	f_s	200
Ochthephilum fracticorne	(Paykull, 1800)	Staphylinidae	8	7	s_s	b_f	h_b	f_z	300
Ochthephilus aureus	(Fauvel, 1871)	Staphylinidae	5	2	s_s	b_f	h_b	f_z	100
Ochthephilus omalinus	(Erichson, 1840)	Staphylinidae	6	4	s_xs	b_f	h_b	f_z	100
Ocypus nitens	(Schrank, 1781)	Staphylinidae	11	11	s_l	b_o	h_b	f_z	300
Ocypus olens	(O. Muller, 1764)	Staphylinidae	6	6	s_xl	b_e	h_b	f_z	>300
Ocypus ophthalmicus	(Scopoli, 1763)	Staphylinidae	7	6	s_l	b_o	h_b	f_z	300
Ocys harpaloides	(Audinet-Serville, 1821)	Carabidae	5	4	s_s	b_f	h_v	f_z	>300
Odacantha melanura	(Linné, 1767)	Carabidae	8	6	s_m	b_f	h_v	f_z	200
Odeles marginata	(Fabricius, 1798)	Scirtidae	4	4	s_s	b_f	h_w	f_s	300
Odonteus armiger	(Scopoli, 1772)	Geotrupidae	7	7	s_m	b_o	h_p	f_m	300
Oedemera femorata	(Scopoli, 1763)	Oedemeridae	6	6	s_m	b_w	h_v	f_p	300
Oedemera flavipes	(Fabricius, 1792)	Oedemeridae	13	10	s_m	b_o	h_v	f_p	300
Oedemera lurida	(Marsham, 1802)	Oedemeridae	10	10	s_m	b_o	h_v	f_p	300
Oedemera podagrariae	(Linné, 1767)	Oedemeridae	6	6	s_m	b_o	h_v	f_p	200
Oedemera pthysica	(Scopoli, 1763)	Oedemeridae	5	5	s_m	b_o	h_v	f_p	>300
Oedemera virescens	(Linné, 1767)	Oedemeridae	8	8	s_m	b_w	h_v	f_p	300
Oenopia conglobata	(Linné, 1758)	Coccinellidae	4	4	s_s	b_w	h_v	f_z	100

Oiceoptoma thoracicum	(Linné, 1758)	Silphidae	5	5	s_l	b_e	h_f	f_n	200
Olibrus aeneus	(Fabricius, 1792)	Phalacridae	6	6	s_xs	b_o	h_v	f_p	200
Olibrus affinis	(J. Sturm, 1807)	Phalacridae	4	4	s_xs	b_o	h_v	f_p	150
Olibrus bicolor	(Fabricius, 1792)	Phalacridae	14	11	s_xs	b_o	h_v	f_p	150
Olibrus corticalis	(Panzer, 1797)	Phalacridae	8	7	s_xs	b_o	h_v	f_p	100
Olibrus liquidus	Erichson, 1845	Phalacridae	10	8	s_xs	b_o	h_v	f_p	150
Olibrus millefolii	(Paykull, 1800)	Phalacridae	10	9	s_xs	b_o	h_v	f_p	150
Oligomerus brunneus	(A. G. Olivier, 1790)	Ptinidae	8	6	s_s	b_w	h_t	f_x	300
Oligota pumilio	Kiesenwetter, 1858	Staphylinidae	5	5	s_xs	b_e	h_e	f_z	150
Omalisus fontisbellaquei	Geoffroy, 1785	Omalisidae	11	10	s_m	b_e	h_b	f_z	300
Omalium oxyacanthae	Gravenhorst, 1806	Staphylinidae	6	4	s_xs	b_e	h_f	f_s	100
Omalium rivulare	(Paykull, 1789)	Staphylinidae	11	10	s_s	b_e	h_f	f_s	300
Omalium rugatum	Mulsant & Rey, 1880	Staphylinidae	5	5	s_s	b_w	h_b	f_s	200
Omonadus floralis	(Linné, 1758)	Anthicidae	4	4	s_s	b_e	h_f	f_z	150
Omophron limbatum	(Fabricius, 1777)	Carabidae	6	6	s_s	b_f	h_b	f_z	>300
Omosita depressa	(Linné, 1758)	Nitidulidae	5	5	s_s	b_w	h_f	f_z	200
Omphalapion hookerorum	(Kirby, 1808)	Brentidae	8	7	s_xs	b_o	h_v	f_p	200
Ontholestes haroldi	(Eppelsheim, 1884)	Staphylinidae	5	4	s_l	b_o	h_f	f_z	100
Ontholestes murinus	(Linné, 1758)	Staphylinidae	7	6	s_l	b_o	h_f	f_z	300
Ontholestes tessellatus	(Geoffroy, 1785)	Staphylinidae	5	4	s_l	b_e	h_f	f_z	200
Onthophagus coenobita	(Herbst, 1783)	Scarabaeidae	4	4	s_m	b_o	h_f	f_c	150
Onthophagus fracticornis	(Preyssler, 1790)	Scarabaeidae	12	8	s_m	b_o	h_f	f_c	300
Onthophagus joannae	Goljan, 1953	Scarabaeidae	7	6	s_s	b_o	h_f	f_c	200
Onthophagus ovatus	(Linné, 1758)	Scarabaeidae	6	5	s_s	b_e	h_f	f_c	150
Onthophagus similis	(Scriba, 1790)	Scarabaeidae	20	12	s_s	b_o	h_f	f_c	300
Onthophagus verticicornis	(Laicharting, 1781)	Scarabaeidae	8	6	s_m	b_w	h_f	f_c	>300
Oodes helopioides	(Fabricius, 1792)	Carabidae	6	6	s_m	b_f	h_b	f_z	200
Opatrum sabulosum	(Linné, 1760)	Tenebrionidae	6	5	s_m	b_o	h_b	f_s	150
Ophonus ardosiacus	Lutshnik, 1922	Carabidae	7	4	s_l	b_o	h_b	f_p	200
Ophonus azureus	(Fabricius, 1775)	Carabidae	5	5	s_m	b_o	h_b	f_p	300
Ophonus laticollis	(Mannerheim, 1825)	Carabidae	5	4	s_m	b_o	h_b	f_p	300
Ophonus melletii	Heer, 1837	Carabidae	10	7	s_m	b_o	h_b	f_p	150
Ophonus rufibarbis	(Fabricius, 1792)	Carabidae	12	10	s_m	b_o	h_b	f_p	300
Opilo mollis	(Linné, 1758)	Cleridae	10	10	s_m	b_w	h_t	f_z	300
Opsilia coerulescens	(Scopoli, 1763)	Cerambycidae	14	11	s_l	b_o	h_v	f_p	300
Orchesia micans	(Panzer, 1793)	Melandryidae	5	5	s_s	b_w	h_t	f_m	300
Orchestes fagi	(Linné, 1758)	Curculionidae	18	11	s_xs	b_w	h_v	f_p	>300
Orchestes hortorum	(Fabricius, 1792)	Curculionidae	8	7	s_xs	b_w	h_v	f_p	150
Orchestes pilosus	(Fabricius, 1781)	Curculionidae	5	5	s_s	b_w	h_v	f_p	200
Orchestes quercus	(Linné, 1758)	Curculionidae	5	5	s_s	b_w	h_v	f_p	300
Orchestes testaceus	(O. F. Müller, 1776)	Curculionidae	7	7	s_s	b_w	h_v	f_p	300
Orectochilus villosus	(O. F. Müller, 1776)	Gyrinidae	17	13	s_m	b_f	h_w	f_z	300
Oreina alpestris	(J. Weise, 1883)	Chrysomelidae	4	3	s_m	b_w	h_v	f_p	150
Oreina cacaliae	(Schrank, 1785)	Chrysomelidae	7	3	s_m	b_w	h_v	f_p	100
Oreina speciosa	(Linné, 1767)	Chrysomelidae	11	8	s_l	b_f	h_v	f_p	100
Oreodytes sanmarkii	(C. R. Sahlberg, 1826)	Dytiscidae	7	4	s_xs	b_f	h_w	f_z	100
Oreonebria castanea	(Bonelli, 1810)	Carabidae	10	6	s_m	b_e	h_b	f_z	100

Orsodacne cerasi	(Linné, 1758)	Orsodacnidae	7	7	s_m	b_w	h_v	f_p	200
Orthocis alni	(Gyllenhal, 1813)	Ciidae	5	5	s_xs	b_w	h_t	f_m	300
Orthocis lucasi	(Abeille de Perrin, 1874)	Ciidae	4	3	s_xs	b_w	h_t	f_m	100
Orthoperus atomus	(Gyllenhal, 1808)	Corylophidae	7	5	s_xs	b_w	h_t	f_z	300
Orthoperus intersitus	Bruce, 1951	Corylophidae	6	4	s_xs	b_f	h_f	f_z	150
Orthotomicus laricis	(Fabricius, 1792)	Curculionidae	4	4	s_s	b_w	h_t	f_x	150
Othius punctulatus	(Goeze, 1777)	Staphylinidae	6	5	s_l	b_w	h_b	f_z	150
Othius subuliformis	Stephens, 1833	Staphylinidae	6	6	s_s	b_w	h_b	f_z	200
Otiorhynchus carinatopunctatus	(Retzius, 1783)	Curculionidae	5	4	s_s	b_e	h_v	f_p	200
Otiorhynchus coecus	Germar, 1824	Curculionidae	7	6	s_l	b_w	h_v	f_p	150
Otiorhynchus gemmatus	(Scopoli, 1763)	Curculionidae	10	9	s_m	b_f	h_v	f_p	100
Otiorhynchus lepidopterus	(Fabricius, 1794)	Curculionidae	7	7	s_m	b_w	h_v	f_p	200
Otiorhynchus lirus	Schönherr, 1834	Curculionidae	8	7	s_m	b_e	h_b	f_p	100
Otiorhynchus morio	(Fabricius, 1781)	Curculionidae	4	4	s_l	b_e	h_v	f_p	200
Otiorhynchus ovatus	(Linné, 1758)	Curculionidae	4	4	s_s	b_e	h_v	f_p	150
Otiorhynchus pseudonothus	Apfelbeck, 1897	Curculionidae	5	4	s_l	b_e	h_v	f_p	100
Otiorhynchus pupillatus	Gyllenhal, 1834	Curculionidae	7	5	s_m	b_w	h_v	f_p	200
Otiorhynchus singularis	(Linné, 1767)	Curculionidae	5	5	s_m	b_e	h_v	f_p	200
Otiorhynchus tenebricosus	(Herbst, 1784)	Curculionidae	5	4	s_l	b_w	h_v	f_p	300
Oulema duftschmidi	(L. Redtenbacher, 1874)	Chrysomelidae	6	6	s_s	b_e	h_v	f_p	200
Oulema gallaeciana	(Heyden, 1870)	Chrysomelidae	5	5	s_s	b_e	h_v	f_p	150
Oxyomus sylvestris	(Scopoli, 1763)	Scarabaeidae	8	7	s_s	b_e	h_f	f_s	200
Oxypoda alternans	(Gravenhorst, 1802)	Staphylinidae	7	5	s_s	b_w	h_p	f_z	300
Oxypoda annularis	(Mannerheim, 1830)	Staphylinidae	9	9	s_xs	b_w	h_b	f_z	300
Oxypoda brevicornis	(Stephens, 1832)	Staphylinidae	4	4	s_xs	b_e	h_f	f_z	200
Oxypselaphus obscurus	(Herbst, 1784)	Carabidae	4	4	s_s	b_f	h_b	f_z	300
Oxystoma craccae	(Linné, 1767)	Brentidae	8	7	s_xs	b_e	h_v	f_p	100
Oxystoma ochropus	(Germar, 1818)	Brentidae	5	3	s_s	b_o	h_v	f_p	150
Oxystoma pomonae	(Fabricius, 1798)	Brentidae	6	4	s_s	b_o	h_v	f_p	100
Oxytelus piceus	(Linné, 1767)	Staphylinidae	10	7	s_s	b_o	h_f	f_s	>300
Oxythyrea funesta	(Poda von Neuhaus, 1761)	Scarabaeidae	5	5	s_m	b_o	h_v	f_s	>300
Pachybrachis picus	(J. Weise, 1882)	Chrysomelidae	8	4	s_s	b_w	h_v	f_p	100
Pachyta quadrimaculata	(Linné, 1758)	Cerambycidae	12	12	s_l	b_w	h_t	f_x	100
Pachytodes cerambyciformis	(Schrank, 1781)	Cerambycidae	8	8	s_m	b_w	h_t	f_x	300
Paederidus ruficollis	(Fabricius, 1777)	Staphylinidae	5	3	s_m	b_f	h_b	f_z	200
Paederus fuscipes	Curtis, 1826	Staphylinidae	10	10	s_m	b_f	h_b	f_z	>300
Paederus littoralis	Gravenhorst, 1802	Staphylinidae	4	4	s_m	b_o	h_b	f_z	150
Paederus riparius	(Linné, 1758)	Staphylinidae	10	10	s_m	b_f	h_b	f_z	300
Palorus depressus	(Fabricius, 1790)	Tenebrionidae	6	6	s_s	b_e	h_e	f_z	200
Panagaeus cruxmajor	(Linné, 1758)	Carabidae	5	5	s_m	b_f	h_b	f_z	300
Paradromius linearis	(A. G. Olivier, 1795)	Carabidae	16	15	s_s	b_o	h_v	f_z	300
Paramecosoma melanocephalum	(Herbst, 1793)	Cryptophagidae	4	4	s_xs	b_f	h_b	f_m	300
Paranchus albipes	(Fabricius, 1796)	Carabidae	5	4	s_m	b_f	h_b	f_z	>300
Paratachys bistriatus	(Duftschmid, 1812)	Carabidae	6	4	s_xs	b_f	h_b	f_z	150
Parethelcus pollinarius	(Forster, 1771)	Curculionidae	7	7	s_s	b_o	h_v	f_p	200
Paromalus flavicornis	(Herbst, 1792)	Histeridae	5	5	s_xs	b_w	h_t	f_z	200

Parophonus maculicornis	(Duftschmid, 1812)	Carabidae	4	4	s_m	b_o	h_b	f_p	100
Patrobus atrorufus	(Ström, 1768)	Carabidae	7	6	s_m	b_w	h_b	f_z	300
Pediacus depressus	(Herbst, 1797)	Cucujidae	4	3	s_s	b_w	h_t	f_z	200
Peltodytes caesus	(Duftschmid, 1805)	Haliplidae	5	5	s_s	b_f	h_w	f_p	300
Pentaphyllus testaceus	(Hellwig, 1792)	Tenebrionidae	6	5	s_xs	b_w	h_t	f_x	150
Perapion curtirostre	(Germar, 1817)	Brentidae	10	10	s_xs	b_e	h_v	f_p	200
Perapion marchicum	(Herbst, 1797)	Apionidae	8	5	s_xs	b_e	h_v	f_p	200
Perapion violaceum	(Kirby, 1808)	Apionidae	13	12	s_s	b_e	h_v	f_p	300
Perileptus areolatus	(Creutzer, 1799)	Carabidae	5	4	s_xs	b_f	h_b	f_z	>300
Peritelus sphaeroides	Germar, 1824	Curculionidae	9	8	s_m	b_o	h_v	f_p	150
Phacophallus parumpunctatus	(Gyllenhal, 1827)	Staphylinidae	6	5	s_s	b_o	h_f	f_z	150
Phaedon armoraciae	(Linné, 1758)	Chrysomelidae	4	4	s_s	b_f	h_v	f_p	150
Phaedon cochleariae	(Fabricius, 1792)	Chrysomelidae	7	6	s_s	b_f	h_v	f_p	300
Phalacrus fimetarius	(Fabricius, 1775)	Phalacridae	4	4	s_xs	b_o	h_v	f_m	150
Pheletes aeneoniger	(DeGeer, 1774)	Elateridae	6	6	s_m	b_w	h_v	f_p	300
Pheletes quercus	(A. G. Olivier, 1790)	Elateridae	4	4	s_s	b_o	h_v	f_p	200
Philonthus carbonarius	(Gravenhorst, 1802)	Staphylinidae	5	4	s_m	b_e	h_b	f_z	150
Philonthus cognatus	Stephens, 1832	Staphylinidae	4	4	s_m	b_e	h_b	f_z	300
Philonthus decorus	(Gravenhorst, 1802)	Staphylinidae	8	7	s_l	b_w	h_b	f_z	>300
Philonthus intermedius	(Lacordaire, 1835)	Staphylinidae	4	3	s_m	b_w	h_f	f_z	150
Philonthus micans	(Gravenhorst, 1802)	Staphylinidae	5	5	s_m	b_f	h_b	f_z	300
Philonthus politus	(Linné, 1758)	Staphylinidae	8	6	s_l	b_e	h_f	f_z	150
Philonthus quisquiliarius	(Gyllenhal, 1810)	Staphylinidae	8	8	s_m	b_f	h_b	f_z	>300
Philonthus rotundicollis	(Ménétriés, 1832)	Staphylinidae	5	5	s_m	b_e	h_b	f_z	200
Philonthus rufimanus	Erichson, 1840	Staphylinidae	4	3	s_m	b_f	h_b	f_z	100
Philonthus sanguinolentus	(Gravenhorst, 1802)	Staphylinidae	5	5	s_m	b_e	h_f	f_z	150
Philonthus spinipes	Sharp, 1874	Staphylinidae	6	6	s_l	b_o	h_f	f_z	300
Philonthus splendens	(Fabricius, 1792)	Staphylinidae	5	4	s_l	b_e	h_f	f_z	150
Philonthus succicola	C. G. Thomson, 1860	Staphylinidae	5	5	s_l	b_e	h_f	f_z	200
Philonthus varians	(Paykull, 1789)	Staphylinidae	4	4	s_m	b_e	h_f	f_z	200
Philopedon plagiatum	(Schaller, 1783)	Curculionidae	7	4	s_s	b_o	h_v	f_p	150
Philothermus evanescens	(Reitter, 1876)	Cerylonidae	9	3	s_xs	b_w	h_t	f_z	100
Phloeocharis subtilissima	Mannerheim, 1830	Staphylinidae	8	7	s_xs	b_w	h_t	f_z	300
Phloeonomus minimus	(Erichson, 1839)	Staphylinidae	5	4	s_xs	b_w	h_t	f_z	100
Phloeonomus punctipennis	C. G. Thomson, 1867	Staphylinidae	10	10	s_xs	b_w	h_t	f_z	300
Phloeonomus pusillus	(Gravenhorst, 1806)	Staphylinidae	4	3	s_xs	b_w	h_t	f_z	150
Phloeopora corticalis	(Gravenhorst, 1802)	Staphylinidae	5	5	s_xs	b_w	h_t	f_z	200
Phloeostiba plana	(Paykull, 1792)	Staphylinidae	7	7	s_xs	b_w	h_t	f_z	150
Phosphuga atrata	(Linné, 1758)	Silphidae	6	6	s_l	b_w	h_e	f_z	300
Phratora laticollis	(Suffrian, 1851)	Chrysomelidae	5	4	s_s	b_w	h_v	f_p	200
Phratora vitellinae	(Linné, 1758)	Chrysomelidae	5	5	s_s	b_w	h_v	f_p	200
Phratora vulgatissima	(Linné, 1758)	Chrysomelidae	5	3	s_s	b_w	h_v	f_p	100
Phrissotrichum rugicolle	(Germar, 1817)	Brentidae	7	2	s_xs	b_o	h_v	f_p	100
Phyllobius arborator	(Herbst, 1797)	Curculionidae	14	11	s_m	b_w	h_v	f_p	300
Phyllobius argentatus	(Linné, 1758)	Curculionidae	17	16	s_s	b_w	h_v	f_p	200
Phyllobius betulinus	(Bechstein & Scharfenberg	Curculionidae	8	7	s_s	b_w	h_v	f_p	200

Phyllobius glaucus	(Scopoli, 1763)	Curculionidae	15	15	s_m	b_w	h_v	f_p	>300
Phyllobius maculicornis	Germar, 1824	Curculionidae	7	7	s_s	b_w	h_v	f_p	200
Phyllobius oblongus	(Linné, 1758)	Curculionidae	11	11	s_s	b_e	h_v	f_p	300
Phyllobius pomaceus	Gyllenhal, 1834	Curculionidae	13	10	s_m	b_e	h_v	f_p	200
Phyllobius pyri	(Linné, 1758)	Curculionidae	14	13	s_m	b_e	h_v	f_p	300
Phyllobius roboretanus	Gredler, 1882	Curculionidae	14	12	s_s	b_e	h_v	f_p	150
Phyllobius virideaeris	(Laicharting, 1781)	Curculionidae	10	9	s_s	b_o	h_v	f_p	150
Phyllobius viridicollis	(Fabricius, 1792)	Curculionidae	9	6	s_s	b_e	h_v	f_p	>300
Phyllobrotica quadrimaculata	(Linné, 1758)	Chrysomelidae	11	8	s_m	b_f	h_v	f_p	200
Phyllodrepa floralis	(Paykull, 1789)	Staphylinidae	6	6	s_s	b_e	h_n	f_z	300
Phyllodrepa nigra	(Gravenhorst, 1806)	Staphylinidae	6	5	s_s	b_w	h_t	f_z	150
Phyllopertha horticola	(Linné, 1758)	Scarabaeidae	11	8	s_m	b_e	h_v	f_p	>300
Phyllotreta astrachanica	Lopatin, 1977	Chrysomelidae	4	4	s_xs	b_o	h_v	f_p	100
Phyllotreta atra	(Fabricius, 1775)	Chrysomelidae	6	6	s_xs	b_e	h_v	f_p	150
Phyllotreta cruciferae	(Goeze, 1777)	Chrysomelidae	5	5	s_xs	b_o	h_v	f_p	100
Phyllotreta exclamationis	(Thunberg, 1784)	Chrysomelidae	5	4	s_xs	b_f	h_v	f_p	150
Phyllotreta nemorum	(Linné, 1758)	Chrysomelidae	5	5	s_xs	b_e	h_v	f_p	300
Phyllotreta nigripes	(Fabricius, 1775)	Chrysomelidae	6	6	s_xs	b_e	h_v	f_p	150
Phyllotreta nodicornis	(Marsham, 1802)	Chrysomelidae	9	5	s_xs	b_o	h_v	f_p	100
Phyllotreta ochripes	(Curtis, 1837)	Chrysomelidae	5	5	s_s	b_e	h_v	f_p	100
Phyllotreta procera	(L. Redtenbacher, 1849)	Chrysomelidae	11	7	s_xs	b_o	h_v	f_p	100
Phyllotreta tetrastigma	(Comolli, 1837)	Chrysomelidae	5	5	s_xs	b_f	h_v	f_p	300
Phyllotreta undulata	Kutschera, 1860	Chrysomelidae	6	6	s_xs	b_e	h_v	f_p	300
Phyllotreta vittula	(L. Redtenbacher, 1849)	Chrysomelidae	7	7	s_xs	b_e	h_v	f_p	200
Phymatodes testaceus	(Linné, 1758)	Cerambycidae	8	7	s_l	b_w	h_t	f_x	300
Phytobius leucogaster	(Marsham, 1802)	Curculionidae	4	3	s_xs	b_f	h_v	f_p	100
Phytoecia nigricornis	(Fabricius, 1781)	Cerambycidae	6	4	s_m	b_o	h_v	f_p	100
Pidonia lurida	(Fabricius, 1792)	Cerambycidae	8	5	s_m	b_w	h_t	f_x	150
Pissodes pini	(Linné, 1758)	Curculionidae	4	4	s_m	b_w	h_t	f_x	100
Pityogenes chalcographus	(Linné, 1760)	Scolytidae	11	8	s_xs	b_w	h_t	f_x	200
Pityophagus ferrugineus	(Linné, 1761)	Nitidulidae	5	5	s_s	b_w	h_t	f_z	300
Placonotus testaceus	(Fabricius, 1787)	Laemophloeidae	7	6	s_xs	b_w	h_t	f_z	200
Placusa depressa	Mäklin, 1845	Staphylinidae	4	3	s_xs	b_w	h_t	f_z	100
Placusa tachyporoides	(Waltl, 1838)	Staphylinidae	4	4	s_xs	b_w	h_t	f_z	150
Plagionotus arcuatus	(Linné, 1758)	Cerambycidae	6	5	s_l	b_w	h_t	f_x	100
Plagiosterna aenea	(Linné, 1758)	Chrysomelidae	7	4	s_m	b_w	h_v	f_p	>300
Planeustomus palpalis	(Erichson, 1839)	Staphylinidae	6	5	s_xs	b_w	h_b	f_s	100
Platambus maculatus	(Linné, 1758)	Dytiscidae	15	14	s_m	b_f	h_w	f_z	>300
Plateumaris consimilis	(Schrank, 1781)	Chrysomelidae	12	9	s_m	b_f	h_v	f_p	200
Plateumaris sericea	(Linné, 1758)	Chrysomelidae	18	14	s_m	b_f	h_v	f_p	300
Platydema violacea	(Fabricius, 1790)	Tenebrionidae	4	4	s_m	b_w	h_t	f_x	200
Platydracus chalcocephalus	(Fabricius, 1801)	Staphylinidae	4	4	s_l	b_w	h_f	f_z	150
Platydracus stercorarius	(A. G. Olivier, 1795)	Staphylinidae	12	10	s_l	b_o	h_b	f_z	300
Platynaspis luteorubra	(Goeze, 1777)	Coccinellidae	9	8	s_s	b_o	h_v	f_z	200
Platynus livens	(Gyllenhal, 1810)	Carabidae	5	5	s_m	b_w	h_b	f_z	100
Platyrhinus resinosus	(Scopoli, 1763)	Anthribidae	7	6	s_l	b_w	h_t	f_x	200

Platystethus nitens	(C. R. Sahlberg, 1832)	Staphylinidae	8	8	s_xs	b_o	h_b	f_s	300
Platystomos albinus	(Linné, 1758)	Anthribidae	8	8	s_m	b_w	h_t	f_x	300
Plectophloeus nitidus	(Fairmaire, 1858)	Staphylinidae	5	3	s_xs	b_w	h_t	f_z	100
Plectophloeus nubigena	(Reitter, 1877)	Staphylinidae	5	4	s_xs	b_w	h_t	f_z	100
Plegaderus caesus	(Herbst, 1792)	Histeridae	8	6	s_xs	b_w	h_t	f_z	300
Plegaderus dissectus	Erichson, 1839	Histeridae	8	7	s_xs	b_w	h_t	f_z	200
Pleurophorus caesus	(Creutzer, 1796)	Scarabaeidae	9	8	s_xs	b_o	h_f	f_s	100
Pocadius adustus	Reitter, 1888	Nitidulidae	4	4	s_s	b_w	h_p	f_m	200
Pocadius ferrugineus	(Fabricius, 1775)	Nitidulidae	6	6	s_s	b_w	h_p	f_m	200
Podabrus alpinus	(Paykull, 1798)	Cantharidae	7	6	s_l	b_w	h_v	f_z	300
Podagrica fuscicornis	(Linné, 1767)	Chrysomelidae	8	7	s_s	b_o	h_v	f_p	200
Podagrica fuscipes	(Fabricius, 1775)	Chrysomelidae	12	9	s_s	b_o	h_v	f_p	100
Podistra rufotestacea	(Letzner, 1845)	Cantharidae	6	6	s_m	b_w	h_v	f_z	300
Poecilium alni	(Linné, 1767)	Cerambycidae	7	4	s_s	b_w	h_t	f_x	150
Poecilus lepidus	(Leske, 1785)	Carabidae	6	4	s_l	b_o	h_b	f_z	300
Poecilus versicolor	(J. Sturm, 1824)	Carabidae	6	6	s_m	b_o	h_b	f_z	>300
Pogonocherus decoratus	Fairmaire, 1855	Cerambycidae	4	4	s_s	b_w	h_t	f_x	150
Pogonocherus fasciculatus	(DeGeer, 1775)	Cerambycidae	6	5	s_m	b_w	h_t	f_x	200
Pogonocherus hispidus	(Linné, 1758)	Cerambycidae	9	8	s_m	b_w	h_t	f_x	200
Pogonus chalceus	(Marsham, 1802)	Carabidae	4	4	s_m	b_f	h_b	f_z	100
Pogonus iridipennis	(Nicolai, 1822)	Carabidae	4	2	s_m	b_f	h_b	f_z	100
Polydrusus aeratus	(Gravenhorst, 1807)	Curculionidae	14	11	s_s	b_w	h_v	f_p	300
Polydrusus amoenus	(Germar, 1824)	Curculionidae	7	4	s_s	b_w	h_v	f_p	100
Polydrusus cervinus	(Linné, 1758)	Curculionidae	10	9	s_s	b_w	h_v	f_p	300
Polydrusus corruscus	Germar, 1824	Curculionidae	5	4	s_s	b_w	h_v	f_p	150
Polydrusus fulvicornis	(Fabricius, 1792)	Curculionidae	7	4	s_s	b_f	h_v	f_p	100
Polydrusus marginatus	Stephens, 1831	Curculionidae	12	11	s_s	b_w	h_v	f_p	150
Polydrusus mollis	(Ström, 1768)	Curculionidae	5	4	s_m	b_w	h_v	f_p	150
Polydrusus pilosus	Gredler, 1866	Curculionidae	6	4	s_s	b_w	h_v	f_p	>300
Polydrusus pterygomalis	Boheman, 1840	Curculionidae	7	4	s_s	b_w	h_v	f_p	200
Polydrusus tereticollis	(DeGeer, 1775)	Curculionidae	8	7	s_s	b_w	h_v	f_p	300
Polygraphus grandiclava	C. G. Thomson, 1886	Curculionidae	6	3	s_xs	b_w	h_t	f_x	100
Polygraphus poligraphus	(Linné, 1758)	Scolytidae	6	5	s_xs	b_w	h_t	f_x	150
Poophagus sisymbrii	(Fabricius, 1777)	Curculionidae	4	4	s_s	b_f	h_v	f_p	200
Porotachys bisulcatus	(Nicolai, 1822)	Carabidae	7	5	s_xs	b_o	h_f	f_z	300
Potamophilus acuminatus	(Fabricius, 1792)	Elmidae	4	3	s_m	b_f	h_w	f_p	200
Prasocuris junci	(Brahm, 1790)	Chrysomelidae	7	5	s_s	b_f	h_v	f_p	200
Prasocuris phellandrii	(Linné, 1758)	Chrysomelidae	7	5	s_s	b_f	h_v	f_p	100
Pria dulcamarae	(Scopoli, 1763)	Nitidulidae	4	3	s_xs	b_o	h_v	f_p	300
Priobium carpini	(Herbst, 1793)	Ptinidae	6	6	s_s	b_w	h_t	f_x	>300
Prionocyphon serricornis	(P. W. J. Müller, 1821)	Scirtidae	5	5	s_s	b_w	h_w	f_s	300
Prionus coriarius	(Linné, 1758)	Cerambycidae	7	6	s_xl	b_w	h_t	f_x	>300
Procraerus tibialis	(Lacordaire, 1835)	Elateridae	7	6	s_m	b_w	h_t	f_x	200
Propylea quatuordecimpunctata	(Linné, 1758)	Coccinellidae	12	12	s_s	b_e	h_v	f_z	300
Prosternon tessellatum	(Linné, 1758)	Elateridae	9	9	s_l	b_w	h_v	f_e	300
Protaetia marmorata	(Fabricius, 1792)	Scarabaeidae	4	4	s_xl	b_w	h_t	f_x	150

Protaetia metallica	(Paykull, 1799)	Scarabaeidae	13	11	s_l	b_w	h_n	f_x	300
Protapion apricans	(Herbst, 1797)	Brentidae	5	5	s_xs	b_e	h_v	f_p	300
Protapion fulvipes	(Geoffroy, 1785)	Apionidae	13	13	s_xs	b_e	h_v	f_p	300
Protapion nigritarse	(Kirby, 1808)	Brentidae	5	5	s_xs	b_e	h_v	f_p	300
Protapion ononidis	(Gyllenhal, 1827)	Brentidae	9	3	s_xs	b_o	h_v	f_p	300
Protapion trifolii	(Linné, 1768)	Brentidae	15	13	s_xs	b_e	h_v	f_p	300
Proteinus brachypterus	(Fabricius, 1792)	Staphylinidae	8	7	s_xs	b_e	h_f	f_s	200
Proteinus laevigatus	Hochhuth, 1872	Staphylinidae	5	5	s_xs	b_e	h_f	f_s	100
Proteinus ovalis	Stephens, 1834	Staphylinidae	10	10	s_xs	b_e	h_f	f_s	200
Psammodius asper	(Fabricius, 1775)	Scarabaeidae	4	3	s_s	b_o	h_b	f_s	300
Psammoecus bipunctatus	(Fabricius, 1792)	Silvanidae	5	5	s_xs	b_f	h_f	f_z	300
Pselaphus heisei	Herbst, 1791	Pselaphidae	7	7	s_xs	b_e	h_b	f_z	300
Pseudapion rufirostre	(Fabricius, 1775)	Brentidae	5	3	s_xs	b_o	h_v	f_p	150
Pseudocistela ceramboides	(Linné, 1758)	Tenebrionidae	10	9	s_l	b_w	h_t	f_x	150
Pseudoperapion brevirostre	(Herbst, 1797)	Brentidae	13	10	s_xs	b_e	h_v	f_p	300
Pseudorchestes ermischi	(Dieckmann, 1958)	Curculionidae	4	4	s_xs	b_o	h_v	f_p	150
Pseudostenapion simum	(Germar, 1817)	Brentidae	5	5	s_xs	b_o	h_v	f_p	200
Pseudovadonia livida	(Fabricius, 1777)	Cerambycidae	9	9	s_m	b_w	h_b	f_m	300
Psylliodes affinis	(Paykull, 1799)	Chrysomelidae	6	5	s_xs	b_o	h_v	f_p	200
Psylliodes chalcomera	(Illiger, 1807)	Chrysomelidae	8	5	s_s	b_o	h_v	f_p	100
Psylliodes chrysocephala	(Linné, 1758)	Chrysomelidae	13	13	s_s	b_o	h_v	f_p	200
Psylliodes dulcamarae	(Koch, 1803)	Chrysomelidae	6	5	s_s	b_e	h_v	f_p	200
Psylliodes instabilis	Foudras, 1860	Chrysomelidae	8	4	s_xs	b_o	h_v	f_p	100
Psylliodes isatidis	Heikertinger, 1913	Chrysomelidae	6	6	s_s	b_o	h_v	f_p	100
Psylliodes napi	(Fabricius, 1792)	Chrysomelidae	9	9	s_xs	b_e	h_v	f_p	300
Psylliodes picina	(Marsham, 1802)	Chrysomelidae	7	6	s_xs	b_f	h_v	f_p	200
Psylliodes toelgi	Heikertinger, 1914	Chrysomelidae	4	3	s_xs	b_o	h_v	f_p	150
Psyllobora vigintiduopunctata	(Linné, 1758)	Coccinellidae	15	14	s_s	b_o	h_v	f_z	300
Ptenidium fuscicorne	Erichson, 1845	Ptiliidae	5	5	s_xs	b_f	h_b	f_m	200
Ptenidium gressneri	Erichson, 1845	Ptiliidae	10	6	s_xs	b_w	h_t	f_m	200
Ptenidium intermedium	Wankowicz, 1869	Ptiliidae	5	4	s_xs	b_f	h_f	f_m	100
Pterostichus burmeisteri	Heer, 1838	Carabidae	5	5	s_l	b_w	h_b	f_z	300
Pterostichus cristatus	(L. Dufour, 1820)	Carabidae	10	7	s_l	b_w	h_b	f_z	300
Pterostichus diligens	(J. Sturm, 1824)	Carabidae	6	6	s_s	b_f	h_b	f_z	300
Pterostichus fasciatopunctatus	(Creutzer, 1799)	Carabidae	5	4	s_l	b_w	h_b	f_z	100
Pterostichus jurinei	(Panzer, 1803)	Carabidae	8	6	s_l	b_w	h_b	f_z	100
Pterostichus madidus	(Fabricius, 1775)	Carabidae	6	6	s_l	b_e	h_b	f_z	200
Pterostichus melanarius	(Illiger, 1798)	Carabidae	12	10	s_l	b_o	h_b	f_z	>300
Pterostichus minor	(Gyllenhal, 1827)	Carabidae	8	7	s_m	b_f	h_b	f_z	200
Pterostichus niger	(Schaller, 1783)	Carabidae	8	8	s_l	b_w	h_b	f_z	300
Pterostichus nigrita	(Paykull, 1790)	Carabidae	9	9	s_m	b_f	h_b	f_z	300
Pterostichus oblongopunctatus	(Fabricius, 1787)	Carabidae	7	7	s_m	b_w	h_b	f_z	>300
Pterostichus pumilio	(Dejean, 1828)	Carabidae	6	3	s_s	b_w	h_b	f_z	200
Pterostichus strenuus	(Panzer, 1796)	Carabidae	8	7	s_m	b_f	h_b	f_z	300
Pterostichus unctulatus	(Duftschmid, 1812)	Carabidae	5	4	s_m	b_w	h_b	f_z	100
Pterostichus vernalis	(Panzer, 1796)	Carabidae	5	5	s_m	b_e	h_b	f_z	300

Pteryngium crenatum	(Fabricius, 1798)	Cryptophagidae	7	5	s_xs	b_w	h_t	f_m	300
Pteryx suturalis	(Heer, 1841)	Ptiliidae	14	12	s_xs	b_w	h_t	f_m	300
Ptilinus pectinicornis	(Linné, 1758)	Ptinidae	5	4	s_s	b_w	h_t	f_x	200
Ptinella aptera	(Guérin-Méneville, 1839)	Ptiliidae	5	3	s_xs	b_w	h_t	f_m	200
Ptinomorphus imperialis	(Linné, 1767)	Ptinidae	7	7	s_s	b_w	h_t	f_x	200
Ptinus fur	(Linné, 1758)	Ptinidae	6	5	s_s	b_e	h_f	f_s	100
Ptinus lichenum	Marsham, 1802	Ptinidae	4	3	s_xs	b_w	h_t	f_x	100
Ptinus rufipes	A. G. Olivier, 1790	Ptinidae	7	7	s_s	b_w	h_t	f_x	200
Ptinus sexpunctatus	Panzer, 1789	Ptinidae	7	7	s_s	b_e	h_t	f_s	200
Ptinus subpilosus	J. Sturm, 1837	Ptinidae	4	3	s_xs	b_w	h_b	f_s	150
Ptosima undecimmaculata	(Herbst, 1784)	Buprestidae	8	2	s_m	b_o	h_t	f_x	100
Pyrochroa coccinea	(Linné, 1760)	Pyrochroidae	6	6	s_l	b_w	h_t	f_x	200
Pyrochroa serraticornis	(Scopoli, 1763)	Pyrochroidae	6	6	s_l	b_w	h_t	f_x	200
Pyropterus nigroruber	(DeGeer, 1774)	Lycidae	6	5	s_m	b_w	h_t	f_z	300
Pyrrhalta viburni	(Paykull, 1799)	Chrysomelidae	9	6	s_s	b_w	h_v	f_p	200
Pyrrhidium sanguineum	(Linné, 1758)	Cerambycidae	6	6	s_m	b_w	h_t	f_x	200
Pytho depressus	(Linné, 1767)	Pythidae	4	3	s_l	b_w	h_t	f_x	150
Quasimus minutissimus	(Germar, 1823)	Elateridae	7	5	s_xs	b_o	h_v	f_e	100
Quedius brevicornis	C. G. Thomson, 1860	Staphylinidae	7	6	s_m	b_w	h_t	f_z	150
Quedius cinctus	(Paykull, 1790)	Staphylinidae	9	9	s_m	b_e	h_f	f_z	300
Quedius cruentus	(A. G. Olivier, 1795)	Staphylinidae	6	6	s_m	b_e	h_f	f_z	200
Quedius fumatus	(Stephens, 1833)	Staphylinidae	5	4	s_m	b_w	h_b	f_z	300
Quedius lateralis	(Gravenhorst, 1802)	Staphylinidae	6	6	s_l	b_w	h_p	f_z	200
Quedius limbatus	(Heer, 1839)	Staphylinidae	4	3	s_s	b_w	h_b	f_z	100
Quedius maurorufus	(Gravenhorst, 1806)	Staphylinidae	4	4	s_m	b_f	h_b	f_z	150
Quedius maurus	(C. R. Sahlberg, 1830)	Staphylinidae	5	4	s_m	b_w	h_t	f_z	300
Quedius mesomelinus	(Marsham, 1802)	Staphylinidae	13	13	s_m	b_e	h_e	f_z	>300
Quedius microps	Gravenhorst, 1847	Staphylinidae	5	4	s_s	b_w	h_t	f_z	150
Quedius ochripennis	(Ménétriés, 1832)	Staphylinidae	7	5	s_m	b_e	h_e	f_z	100
Quedius scitus	(Gravenhorst, 1806)	Staphylinidae	4	4	s_m	b_w	h_t	f_z	100
Quedius suturalis	Kiesenwetter, 1845	Staphylinidae	7	5	s_m	b_w	h_b	f_z	300
Quedius truncicola	Fairmaire & Laboulbène, 1	Staphylinidae	5	4	s_l	b_w	h_t	f_z	100
Quedius umbrinus	Erichson, 1839	Staphylinidae	4	3	s_m	b_w	h_b	f_z	100
Reichenbachia juncorum	(Leach, 1817)	Pselaphidae	4	2	s_xs	b_f	h_b	f_z	100
Rhagium inquisitor	(Linné, 1758)	Cerambycidae	4	4	s_l	b_w	h_t	f_x	200
Rhagium mordax	(DeGeer, 1775)	Cerambycidae	10	10	s_l	b_w	h_t	f_x	300
Rhagonycha atra	(Linné, 1767)	Cantharidae	5	5	s_s	b_w	h_v	f_z	150
Rhagonycha fulva	(Scopoli, 1763)	Cantharidae	11	10	s_m	b_o	h_v	f_z	200
Rhagonycha gallica	Pic, 1923	Cantharidae	6	4	s_m	b_w	h_v	f_z	100
Rhagonycha lignosa	(O.F. Müller, 1764)	Cantharidae	7	7	s_m	b_e	h_v	f_z	300
Rhagonycha lutea	(O.F. Müller, 1764)	Cantharidae	5	5	s_m	b_o	h_v	f_z	200
Rhagonycha nigriventris	Motschulsky, 1860	Cantharidae	4	4	s_s	b_w	h_v	f_z	300
Rhagonycha testacea	(Linné, 1758)	Cantharidae	5	5	s_s	b_w	h_v	f_z	300
Rhagonycha translucida	(Krynicki, 1832)	Cantharidae	8	7	s_m	b_w	h_v	f_z	300
Rhamphus oxyacanthae	(Marsham, 1802)	Curculionidae	5	4	s_xs	b_w	h_v	f_p	150
Rhamphus pulicarius	(Herbst, 1795)	Curculionidae	7	5	s_xs	b_e	h_v	f_p	200

Rhantus grapii	(Gyllenhal, 1808)	Dytiscidae	4	3	s_m	b_f	h_w	f_z	300
Rhantus suturalis	(W. S. MacLeay, 1825)	Dytiscidae	17	13	s_l	b_f	h_w	f_z	300
Rhaphitropis marchica	(Herbst, 1797)	Anthribidae	6	5	s_xs	b_w	h_t	f_x	200
Rhinoncus castor	(Fabricius, 1792)	Curculionidae	7	6	s_xs	b_o	h_v	f_p	100
Rhinoncus henningsi	Wagner, 1936	Curculionidae	4	3	s_xs	b_f	h_v	f_p	150
Rhinoncus pericarpius	(Linné, 1758)	Curculionidae	10	9	s_xs	b_e	h_v	f_p	200
Rhinoncus perpendicularis	(Reich, 1797)	Curculionidae	4	4	s_xs	b_o	h_v	f_p	100
Rhinusa antirrhini	(Paykull, 1800)	Curculionidae	6	5	s_xs	b_o	h_v	f_p	150
Rhinusa neta	(Germar, 1821)	Curculionidae	4	3	s_xs	b_o	h_v	f_p	100
Rhizophagus bipustulatus	(Fabricius, 1792)	Monotomidae	6	6	s_xs	b_w	h_t	f_z	200
Rhizophagus cribratus	Gyllenhal, 1827	Monotomidae	4	2	s_s	b_w	h_t	f_z	200
Rhizophagus depressus	(Fabricius, 1792)	Monotomidae	6	6	s_s	b_w	h_t	f_z	200
Rhizophagus fenestralis	(Linné, 1758)	Monotomidae	5	4	s_xs	b_w	h_t	f_z	200
Rhizophagus ferrugineus	(Paykull, 1800)	Monotomidae	5	5	s_s	b_w	h_t	f_z	200
Rhizophagus perforatus	Erichson, 1845	Monotomidae	4	4	s_s	b_w	h_t	f_z	150
Rhopalapion longirostre	(A. G. Olivier, 1807)	Brentidae	9	7	s_xs	b_o	h_v	f_p	200
Rhyncolus ater	(Linné, 1758)	Curculionidae	4	4	s_s	b_w	h_t	f_x	300
Rhyssemus germanus	(Linné, 1767)	Scarabaeidae	5	4	s_s	b_o	h_b	f_s	200
Riolus subviolaceus	(P. W. J. Müller, 1817)	Elmidae	4	4	s_xs	b_f	h_w	f_p	100
Ropalodontus perforatus	(Gyllenhal, 1813)	Ciidae	5	5	s_xs	b_w	h_t	f_m	300
Rugilus erichsonii	(Fauvel, 1867)	Staphylinidae	4	3	s_s	b_e	h_f	f_z	150
Rugilus orbiculatus	(Paykull, 1789)	Staphylinidae	6	5	s_s	b_e	h_f	f_z	300
Rugilus rufipes	(Germar, 1836)	Staphylinidae	6	6	s_s	b_e	h_f	f_z	300
Rutpela maculata	(Poda von Neuhaus, 1761)	Cerambycidae	15	14	s_l	b_w	h_t	f_x	300
Rybaxis longicornis	(Leach, 1817)	Staphylinidae	7	6	s_xs	b_f	h_b	f_z	300
Salpingus planirostris	(Fabricius, 1787)	Salpingidae	16	15	s_s	b_w	h_t	f_z	200
Salpingus ruficollis	(Linné, 1761)	Salpingidae	9	9	s_s	b_w	h_t	f_z	300
Saperda scalaris	(Linné, 1758)	Cerambycidae	5	5	s_l	b_w	h_t	f_x	300
Scaphidema metallica	(Fabricius, 1792)	Tenebrionidae	9	8	s_m	b_w	h_t	f_x	300
Scaphidium quadrimaculatum	A. G. Olivier, 1790	Staphylinidae	12	11	s_s	b_w	h_t	f_m	300
Scaphisoma agaricinum	(Linné, 1758)	Staphylinidae	6	6	s_xs	b_w	h_t	f_m	300
Scaphisoma boleti	(Panzer, 1793)	Staphylinidae	9	8	s_xs	b_w	h_t	f_m	300
Schizotus pectinicornis	(Linné, 1758)	Pyrochroidae	7	7	s_m	b_w	h_t	f_x	300
Sciaphilus asperatus	(Bonsdorff, 1785)	Curculionidae	11	8	s_s	b_e	h_v	f_p	300
Sciaphobus scitulus	(Germar, 1824)	Curculionidae	4	2	s_s	b_o	h_v	f_p	100
Scolytus intricatus	(Ratzeburg, 1837)	Scolytidae	6	6	s_s	b_w	h_t	f_x	200
Scopaeus gracilis	(Sperk, 1835)	Staphylinidae	4	4	s_s	b_f	h_b	f_z	300
Scopaeus laevigatus	(Gyllenhal, 1827)	Staphylinidae	11	11	s_s	b_f	h_b	f_z	300
Scraptia fuscula	P. W. J. Müller, 1821	Scraptiidae	10	10	s_xs	b_w	h_t	f_x	200
Scydmaenus rufus	P. W. J. Müller & Kunze,	Staphylinidae	4	4	s_xs	b_e	h_f	f_z	200
Scymnus abietis	(Paykull, 1798)	Coccinellidae	6	6	s_xs	b_w	h_v	f_z	300
Scymnus apetzi	Mulsant, 1846	Coccinellidae	8	4	s_xs	b_o	h_v	f_z	100
Scymnus auritus	Thunberg, 1795	Coccinellidae	6	6	s_xs	b_w	h_v	f_z	300
Scymnus ferrugatus	(Moll, 1785)	Coccinellidae	5	5	s_xs	b_e	h_v	f_z	200
Scymnus frontalis	(Fabricius, 1787)	Coccinellidae	8	8	s_xs	b_o	h_v	f_z	300
Scymnus haemorrhoidalis	Herbst, 1797	Coccinellidae	8	8	s_xs	b_w	h_v	f_z	>300

Scymnus interruptus	(Goeze, 1777)	Coccinellidae	6	4	s_xs	b_o	h_v	f_z	150
Scymnus schmidti	Fürsch, 1958	Coccinellidae	5	5	s_xs	b_o	h_v	f_z	200
Scymnus suturalis	Thunberg, 1795	Coccinellidae	8	8	s_xs	b_w	h_v	f_z	200
Selatosomus aeneus	(Linné, 1758)	Elateridae	7	7	s_l	b_o	h_v	f_p	>300
Selatosomus confluens	(Germar, 1836)	Elateridae	4	3	s_l	b_o	h_b	f_p	200
Selatosomus latus	(Fabricius, 1801)	Elateridae	6	5	s_l	b_o	h_v	f_p	150
Sepedophilus bipunctatus	(Gravenhorst, 1802)	Staphylinidae	4	4	s_xs	b_w	h_t	f_m	200
Sepedophilus immaculatus	(Stephens, 1832)	Staphylinidae	5	5	s_xs	b_e	h_b	f_z	200
Sepedophilus littoreus	(Linné, 1758)	Staphylinidae	10	8	s_s	b_e	h_f	f_z	300
Sepedophilus marshami	(Stephens, 1832)	Staphylinidae	7	7	s_s	b_o	h_b	f_z	200
Sepedophilus obtusus	(Luze, 1902)	Staphylinidae	8	7	s_xs	b_e	h_b	f_z	200
Sepedophilus testaceus	(Fabricius, 1792)	Staphylinidae	9	9	s_s	b_w	h_t	f_m	300
Sericoderus lateralis	(Gyllenhal, 1827)	Corylophidae	10	9	s_xs	b_e	h_f	f_z	200
Sericus brunneus	(Linné, 1758)	Elateridae	6	6	s_m	b_o	h_v	f_e	200
Sermylassa halensis	(Linné, 1767)	Chrysomelidae	6	6	s_m	b_o	h_v	f_p	200
Siagonium quadricorne	Kirby & Spence, 1815	Staphylinidae	5	4	s_s	b_w	h_t	f_z	200
Sibinia pellucens	(Scopoli, 1772)	Curculionidae	7	7	s_s	b_o	h_v	f_p	300
Silis ruficollis	(Fabricius, 1775)	Cantharidae	4	4	s_m	b_f	h_v	f_z	150
Silpha carinata	Herbst, 1783	Silphidae	5	5	s_l	b_e	h_b	f_z	100
Silpha obscura	Linné, 1758	Silphidae	6	5	s_l	b_o	h_b	f_z	300
Silpha tyrolensis	Laicharting, 1781	Silphidae	11	8	s_l	b_e	h_b	f_z	200
Silusa rubiginosa	Erichson, 1837	Staphylinidae	5	2	s_s	b_w	h_t	f_z	100
Silvanoprus fagi	(Guérin-Méneville, 1844)	Silvanidae	5	5	s_xs	b_w	h_t	f_z	200
Silvanus bidentatus	(Fabricius, 1792)	Silvanidae	5	4	s_s	b_w	h_t	f_z	300
Silvanus unidentatus	(A. G. Olivier, 1790)	Silvanidae	4	4	s_xs	b_w	h_t	f_z	100
Simo hirticornis	(Herbst, 1795)	Curculionidae	7	5	s_m	b_w	h_v	f_p	300
Simplocaria semistriata	(Fabricius, 1794)	Byrrhidae	5	5	s_xs	b_e	h_b	f_p	300
Sinodendron cylindricum	(Linné, 1758)	Lucanidae	5	5	s_l	b_w	h_t	f_x	200
Sitona ambiguus	Gyllenhal, 1834	Curculionidae	5	3	s_s	b_o	h_v	f_p	100
Sitona hispidulus	(Fabricius, 1777)	Curculionidae	5	5	s_s	b_o	h_v	f_p	300
Sitona humeralis	Stephens, 1831	Curculionidae	12	11	s_s	b_o	h_v	f_p	300
Sitona languidus	Gyllenhal, 1834	Curculionidae	8	4	s_s	b_o	h_v	f_p	150
Sitona lineatus	(Linné, 1758)	Curculionidae	11	9	s_s	b_e	h_v	f_p	300
Sitona macularius	(Marsham, 1802)	Curculionidae	4	4	s_s	b_o	h_v	f_p	150
Sitona obsoletus	(Gmelin, 1790)	Curculionidae	6	6	s_s	b_o	h_v	f_p	200
Sitona striatellus	Gyllenhal, 1834	Curculionidae	7	6	s_s	b_o	h_v	f_p	100
Sitona sulcifrons	(Thunberg, 1798)	Curculionidae	12	12	s_s	b_o	h_v	f_p	300
Sitona suturalis	Stephens, 1831	Curculionidae	8	7	s_s	b_f	h_v	f_p	200
Smaragdina affinis	(Illiger, 1794)	Chrysomelidae	8	7	s_s	b_o	h_v	f_p	200
Smaragdina aurita	(Linné, 1767)	Chrysomelidae	6	6	s_s	b_o	h_v	f_p	200
Smaragdina flavicollis	(Charpentier, 1825)	Chrysomelidae	5	4	s_s	b_f	h_v	f_p	300
Smaragdina salicina	(Scopoli, 1763)	Chrysomelidae	4	4	s_s	b_o	h_v	f_p	300
Smicronyx jungermanniae	(Reich, 1797)	Curculionidae	6	4	s_xs	b_o	h_v	f_p	100
Soronia grisea	(Linné, 1758)	Nitidulidae	4	4	s_s	b_w	h_v	f_s	300
Soronia punctatissima	(Illiger, 1794)	Nitidulidae	4	4	s_s	b_w	h_v	f_s	150
Spavius glaber	(Gyllenhal, 1808)	Cryptophagidae	4	4	s_xs	b_w	h_n	f_m	300
Sphaeridium bipustulatum	Thunberg, 1794	Hydrophilidae	6	5	s_s	b_e	h_f	f_c	300

Sphaeridium lunatum	Fabricius, 1792	Hydrophilidae	5	4	s_m	b_e	h_f	f_c	100
Sphaeridium scarabaeoides	(Linné, 1758)	Hydrophilidae	6	5	s_m	b_e	h_f	f_c	200
Sphaeroderma testaceum	(Fabricius, 1775)	Chrysomelidae	5	5	s_s	b_o	h_v	f_p	200
Sphaerosoma piliferum	(P. W. J. Müller, 1821)	Alexiidae	4	3	s_xs	b_w	h_b	f_m	100
Sphaerosoma pilosum	(Panzer, 1793)	Alexiidae	8	6	s_xs	b_w	h_b	f_m	200
Sphindus dubius	(Gyllenhal, 1808)	Sphindidae	16	14	s_xs	b_w	h_t	f_m	300
Spondylis buprestoides	(Linné, 1758)	Cerambycidae	6	6	s_l	b_w	h_t	f_x	200
Squamapion atomarium	(Kirby, 1808)	Apionidae	5	4	s_xs	b_e	h_v	f_p	200
Squamapion flavimanum	(Gyllenhal, 1833)	Brentidae	5	3	s_xs	b_o	h_v	f_p	200
Staphylinus erythropterus	Linné, 1758	Staphylinidae	5	3	s_l	b_w	h_b	f_z	100
Stasiodis parvulus	(Fabricius, 1792)	Curculionidae	5	4	s_s	b_o	h_v	f_p	100
Stenagostus rhombeus	(A. G. Olivier, 1790)	Elateridae	10	7	s_l	b_w	h_t	f_z	200
Stenelmis canaliculata	(Gyllenhal, 1808)	Elmidae	5	5	s_s	b_f	h_w	f_p	100
Stenichnus collaris	(P. W. J. Müller & Kunze,	Staphylinidae	5	5	s_xs	b_w	h_b	f_z	200
Stenichnus godarti	(Latreille, 1806)	Staphylinidae	5	5	s_xs	b_w	h_t	f_z	150
Stenichnus scutellaris	(P. W. J. Müller & Kunze,	Staphylinidae	7	7	s_xs	b_e	h_b	f_z	300
Stenocarus ruficornis	(Stephens, 1831)	Curculionidae	9	6	s_s	b_o	h_v	f_p	200
Stenocorus meridianus	(Linné, 1758)	Cerambycidae	5	4	s_l	b_w	h_t	f_x	200
Stenolophus mixtus	(Herbst, 1784)	Carabidae	14	12	s_s	b_f	h_b	f_z	300
Stenolophus teutonus	(Schrank, 1781)	Carabidae	13	11	s_m	b_o	h_b	f_z	>300
Stenomax aeneus	(Scopoli, 1763)	Tenebrionidae	5	4	s_l	b_w	h_t	f_x	200
Stenopterapion meliloti	(Kirby, 1808)	Brentidae	7	6	s_xs	b_e	h_v	f_p	300
Stenopterapion tenue	(Kirby, 1808)	Brentidae	9	8	s_xs	b_e	h_v	f_p	300
Stenopterus rufus	(Linné, 1767)	Cerambycidae	4	4	s_l	b_w	h_t	f_x	200
Stenurella bifasciata	(O. F. Müller, 1776)	Cerambycidae	11	10	s_m	b_w	h_t	f_x	>300
Stenurella melanura	(Linné, 1758)	Cerambycidae	18	17	s_m	b_w	h_t	f_x	300
Stenurella nigra	(Linné, 1758)	Cerambycidae	9	9	s_m	b_w	h_t	f_x	300
Stenus bifoveolatus	Gyllenhal, 1827	Staphylinidae	4	4	s_s	b_f	h_b	f_z	150
Stenus bimaculatus	Gyllenhal, 1810	Staphylinidae	5	5	s_m	b_f	h_b	f_z	200
Stenus boops	Ljungh, 1810	Staphylinidae	13	8	s_s	b_f	h_b	f_z	>300
Stenus cicindeloides	(Schaller, 1783)	Staphylinidae	7	7	s_s	b_f	h_b	f_z	300
Stenus clavicornis	(Scopoli, 1763)	Staphylinidae	5	5	s_s	b_e	h_b	f_z	200
Stenus comma	LeConte, 1863	Staphylinidae	4	4	s_s	b_f	h_b	f_z	300
Stenus crassus	Stephens, 1833	Staphylinidae	4	3	s_s	b_o	h_f	f_z	200
Stenus flavipalpis	C. G. Thomson, 1860	Staphylinidae	5	5	s_s	b_f	h_b	f_z	150
Stenus flavipes	Stephens, 1833	Staphylinidae	5	5	s_s	b_e	h_b	f_z	150
Stenus fornicatus	Stephens, 1833	Staphylinidae	4	4	s_xs	b_f	h_b	f_z	150
Stenus fulvicornis	Stephens, 1833	Staphylinidae	4	4	s_s	b_f	h_b	f_z	200
Stenus guttula	P. W. J. Müller, 1821	Staphylinidae	5	4	s_s	b_f	h_b	f_z	100
Stenus humilis	Erichson, 1839	Staphylinidae	9	8	s_s	b_e	h_b	f_z	>300
Stenus impressus	Germar, 1824	Staphylinidae	8	8	s_s	b_e	h_b	f_z	>300
Stenus juno	(Paykull, 1789)	Staphylinidae	5	5	s_s	b_f	h_b	f_z	300
Stenus latifrons	Erichson, 1839	Staphylinidae	4	4	s_s	b_f	h_b	f_z	150
Stenus nitidiusculus	Stephens, 1833	Staphylinidae	4	4	s_s	b_w	h_b	f_z	300
Stenus providus	Erichson, 1839	Staphylinidae	8	6	s_s	b_f	h_b	f_z	300
Stenus pubescens	Stephens, 1833	Staphylinidae	7	6	s_s	b_f	h_b	f_z	150
Stenus similis	(Herbst, 1784)	Staphylinidae	9	9	s_s	b_e	h_b	f_z	300

Stenus solutus	Erichson, 1840	Staphylinidae	9	5	s_s	b_f	h_b	f_z	150
Stenus tarsalis	Ljungh, 1810	Staphylinidae	5	5	s_s	b_f	h_b	f_z	200
Stephostethus lardarius	(DeGeer, 1775)	Latridiidae	7	5	s_xs	b_e	h_e	f_m	150
Stereonychus fraxini	(DeGeer, 1775)	Curculionidae	8	7	s_s	b_w	h_v	f_p	200
Stethorus pusillus	(Herbst, 1797)	Coccinellidae	9	8	s_xs	b_w	h_v	f_z	200
Stictoleptura maculicornis	(DeGeer, 1775)	Cerambycidae	12	11	s_m	b_w	h_t	f_x	>300
Stictoleptura rubra	(Linné, 1758)	Cerambycidae	16	15	s_l	b_w	h_t	f_x	>300
Stilbus testaceus	(Panzer, 1797)	Phalacridae	5	5	s_xs	b_e	h_v	f_m	150
Stomis pumicatus	(Panzer, 1796)	Carabidae	5	5	s_m	b_f	h_b	f_z	150
Strophosoma capitatum	(DeGeer, 1775)	Curculionidae	7	6	s_s	b_w	h_v	f_p	200
Strophosoma melanogrammum	(Forster, 1771)	Curculionidae	9	9	s_s	b_w	h_v	f_p	200
Subcoccinella vigintiquatuorpunctata	(Linné, 1758)	Coccinellidae	12	9	s_s	b_o	h_v	f_p	300
Sulcacis fronticornis	(Panzer, 1805)	Ciidae	5	5	s_xs	b_w	h_t	f_m	200
Sulcacis nitidus	(Fabricius, 1792)	Cisidae	7	6	s_xs	b_w	h_t	f_m	150
Sunius melanocephalus	(Fabricius, 1792)	Staphylinidae	6	5	s_s	b_o	h_b	f_z	150
Symbiotes gibberosus	(P. H. Lucas, 1846)	Endomychidae	7	4	s_xs	b_w	h_t	f_m	100
Synapion ebeninum	(Kirby, 1808)	Brentidae	7	7	s_xs	b_e	h_v	f_p	150
Synaptus filiformis	(Fabricius, 1781)	Elateridae	5	4	s_m	b_f	h_v	f_e	150
Synchita humeralis	(Fabricius, 1792)	Zopheridae	15	14	s_s	b_w	h_t	f_m	200
Synchita variegata	Hellwig, 1792	Zopheridae	4	3	s_xs	b_w	h_t	f_m	100
Syntomium aeneum	(P. W. J. Müller, 1821)	Staphylinidae	5	5	s_xs	b_w	h_b	f_z	300
Syntomus foveatus	(Geoffroy, 1785)	Carabidae	6	5	s_s	b_o	h_b	f_z	>300
Syntomus truncatellus	(Linné, 1761)	Carabidae	10	8	s_xs	b_e	h_b	f_z	300
Tachinus fimetarius	Gravenhorst, 1802	Staphylinidae	6	5	s_s	b_e	h_f	f_z	150
Tachinus humeralis	Gravenhorst, 1802	Staphylinidae	7	7	s_m	b_w	h_f	f_z	200
Tachinus laticollis	Gravenhorst, 1802	Staphylinidae	11	11	s_s	b_e	h_f	f_z	300
Tachinus lignorum	(Linné, 1758)	Staphylinidae	5	3	s_m	b_o	h_f	f_z	100
Tachinus marginellus	(Fabricius, 1781)	Staphylinidae	7	6	s_s	b_e	h_f	f_z	300
Tachinus pallipes	Gravenhorst, 1806	Staphylinidae	7	6	s_s	b_e	h_f	f_z	300
Tachinus rufipes	(Linné, 1758)	Staphylinidae	11	10	s_s	b_e	h_f	f_z	300
Tachinus subterraneus	(Linné, 1758)	Staphylinidae	6	6	s_s	b_e	h_f	f_z	200
Tachyerges decoratus	(Germar, 1821)	Curculionidae	6	4	s_xs	b_w	h_v	f_p	300
Tachyerges salicis	(Linné, 1758)	Curculionidae	5	5	s_xs	b_w	h_v	f_p	200
Tachyerges stigma	(Germar, 1821)	Curculionidae	4	4	s_xs	b_e	h_v	f_p	300
Tachyporus nitidulus	(Fabricius, 1781)	Staphylinidae	9	9	s_xs	b_e	h_b	f_z	200
Tachyporus obtusus	(Linné, 1767)	Staphylinidae	5	5	s_s	b_e	h_b	f_z	200
Tachyporus ruficollis	Gravenhorst, 1802	Staphylinidae	6	6	s_s	b_w	h_b	f_z	300
Tachyporus solutus	Erichson, 1839	Staphylinidae	4	4	s_s	b_o	h_b	f_z	200
Tachyporus transversalis	Gravenhorst, 1806	Staphylinidae	5	5	s_xs	b_f	h_b	f_z	100
Tachyta nana	(Gyllenhal, 1810)	Carabidae	7	6	s_xs	b_w	h_t	f_z	200
Tachyusa coarctata	Erichson, 1837	Staphylinidae	6	5	s_xs	b_f	h_b	f_z	150
Taeniapion urticarium	(Herbst, 1784)	Brentidae	6	5	s_xs	b_e	h_v	f_p	300
Tanymecus palliatus	(Fabricius, 1787)	Curculionidae	8	8	s_m	b_o	h_v	f_p	200
Tanysphyrus lemnae	(Paykull, 1792)	Brachyceridae	11	11	s_xs	b_f	h_v	f_p	300
Tatianaerhynchites aequatus	(Linné, 1767)	Attelabidae	13	10	s_s	b_w	h_v	f_p	200
Telmatophilus typhae	(Fallén, 1802)	Cryptophagidae	4	3	s_xs	b_f	h_v	f_p	100

Temnocerus coeruleus	(Fabricius, 1798)	Attelabidae	4	4	s_xs	b_w	h_v	f_p	300
Tenebrio molitor	Linné, 1758	Tenebrionidae	5	5	s_l	b_e	h_f	f_s	150
Tetartopeus quadratus	(Paykull, 1789)	Staphylinidae	4	3	s_m	b_f	h_b	f_z	150
Tetartopeus terminatus	Gravenhorst, 1802	Staphylinidae	6	6	s_m	b_f	h_b	f_z	200
Tetratoma fungorum	Fabricius, 1790	Tetratomidae	8	7	s_s	b_w	h_t	f_m	200
Tetrops praeustus	(Linné, 1758)	Cerambycidae	8	7	s_s	b_w	h_t	f_x	200
Tetrops starkii	Chevrolat, 1859	Cerambycidae	4	4	s_s	b_w	h_t	f_x	150
Thamiocolus signatus	(Gyllenhal, 1837)	Curculionidae	6	4	s_xs	b_o	h_v	f_p	100
Thanasimus formicarius	(Linné, 1758)	Cleridae	13	11	s_m	b_w	h_t	f_z	300
Thanatophilus sinuatus	(Fabricius, 1775)	Silphidae	8	7	s_m	b_e	h_f	f_n	200
Thinodromus arcuatus	(Stephens, 1834)	Staphylinidae	9	9	s_s	b_f	h_b	f_p	200
Thinodromus dilatatus	(Erichson, 1839)	Staphylinidae	5	3	s_s	b_f	h_b	f_p	150
Tillus elongatus	(Linné, 1758)	Cleridae	12	12	s_m	b_w	h_t	f_z	200
Timarcha goettingensis	(Linné, 1758)	Chrysomelidae	6	5	s_m	b_o	h_v	f_p	>300
Timarcha tenebricosa	(Fabricius, 1775)	Chrysomelidae	5	5	s_l	b_o	h_v	f_p	150
Tinotus morion	(Gravenhorst, 1802)	Staphylinidae	4	3	s_xs	b_e	h_f	f_z	100
Tomicus piniperda	(Linné, 1758)	Curculionidae	4	4	s_s	b_w	h_t	f_x	150
Trachodes hispidus	(Linné, 1758)	Curculionidae	4	4	s_s	b_w	h_t	f_x	300
Trachyphloeus alternans	Gyllenhal, 1834	Curculionidae	5	3	s_xs	b_o	h_v	f_p	100
Trachyphloeus scabriculus	(Linné, 1771)	Curculionidae	6	5	s_s	b_o	h_v	f_p	150
Trachys minuta	(Linné, 1758)	Buprestidae	5	5	s_s	b_w	h_v	f_p	300
Trechus obtusus	Erichson, 1837	Carabidae	5	5	s_s	b_e	h_b	f_z	>300
Trechus quadristriatus	(Schrank, 1781)	Carabidae	6	6	s_s	b_e	h_b	f_z	300
Trichius fasciatus	(Linné, 1758)	Scarabaeidae	21	20	s_m	b_w	h_t	f_x	300
Trichius gallicus	Dejean, 1821	Scarabaeidae	5	4	s_l	b_w	h_t	f_x	200
Trichius sexualis	Bedel, 1906	Scarabaeidae	6	6	s_l	b_w	h_t	f_x	300
Trichodes alvearius	(Fabricius, 1792)	Cleridae	8	7	s_l	b_o	h_n	f_z	300
Trichodes apiarius	(Linné, 1758)	Cleridae	10	8	s_l	b_o	h_n	f_z	>300
Trichophya pilicornis	(Gyllenhal, 1810)	Staphylinidae	8	7	s_xs	b_e	h_f	f_z	300
Trichosirocalus troglodytes	(Fabricius, 1787)	Curculionidae	13	10	s_xs	b_o	h_v	f_p	200
Trichotichnus laevicollis	(Duftschmid, 1812)	Carabidae	4	4	s_m	b_w	h_b	f_z	200
Trimium brevicorne	(Reichenbach, 1816)	Staphylinidae	7	7	s_xs	b_w	h_b	f_z	300
Triphyllus bicolor	(Fabricius, 1777)	Mycetophagidae	5	3	s_s	b_w	h_t	f_m	200
Triplax russica	(Linné, 1758)	Erotylidae	5	3	s_s	b_w	h_t	f_m	200
Tritoma bipustulata	Fabricius, 1775	Erotylidae	19	17	s_s	b_w	h_t	f_m	300
Trixagus dermestoides	(Linné, 1767)	Throscidae	5	5	s_s	b_e	h_e	f_m	300
Trixagus meybohmi	Leseigneur, 2005	Throscidae	4	4	s_xs	b_e	h_e	f_m	100
Trixagus obtusus	(Curtís, 1827)	Throscidae	7	5	s_xs	b_o	h_b	f_m	100
Troglops albicans	(Linné, 1767)	Melyridae	6	6	s_xs	b_w	h_t	f_z	150
Trogoderma glabrum	(Herbst, 1783)	Dermestidae	6	6	s_s	b_o	h_n	f_n	200
Tropinota hirta	(Poda von Neuhaus, 1761)	Scarabaeidae	5	4	s_m	b_o	h_v	f_s	>300
Tropiphorus elevatus	(Herbst, 1795)	Curculionidae	6	6	s_m	b_w	h_v	f_p	300
Trypodendron domesticum	(Linné, 1758)	Curculionidae	10	8	s_s	b_w	h_t	f_m	300
Trypodendron lineatum	(A. G. Olivier, 1795)	Curculionidae	7	7	s_s	b_w	h_t	f_m	300
Trypodendron signatum	(Fabricius, 1792)	Curculionidae	4	3	s_s	b_w	h_t	f_m	200
Tychius breviusculus	Desbrochers des Loges, 18	Curculionidae	9	6	s_xs	b_o	h_v	f_p	300

Tychius meliloti	Stephens, 1831	Curculionidae	5	5	s_xs	b_o	h_v	f_p	200
Tychius parallelus	(Panzer, 1794)	Curculionidae	4	4	s_xs	b_o	h_v	f_p	150
Tychius picirostris	(Fabricius, 1787)	Curculionidae	5	5	s_xs	b_o	h_v	f_p	300
Tychius quinquepunctatus	(Linné, 1758)	Curculionidae	5	4	s_s	b_o	h_v	f_p	200
Tychus niger	(Paykull, 1800)	Staphylinidae	11	9	s_xs	b_f	h_b	f_z	200
Typhaea stercorea	(Linné, 1758)	Mycetophagidae	5	5	s_xs	b_e	h_f	f_m	150
Typhaeus typhoeus	(Linné, 1758)	Geotrupidae	7	7	s_l	b_o	h_f	f_c	300
Tyrus mucronatus	(Panzer, 1803)	Staphylinidae	10	9	s_xs	b_w	h_t	f_z	300
Tytthaspis sedecimpunctata	(Linné, 1761)	Coccinellidae	18	16	s_xs	b_o	h_v	f_m	300
Uleiota planatus	(Linné, 1761)	Silvanidae	16	15	s_s	b_w	h_t	f_z	300
Uloma culinaris	(Linné, 1758)	Tenebrionidae	14	7	s_m	b_w	h_t	f_x	150
Uloma rufa	(Piller & Mitterpacher, 1	Tenebrionidae	12	10	s_m	b_w	h_t	f_x	100
Valgus hemipterus	(Linné, 1758)	Scarabaeidae	7	7	s_m	b_w	h_t	f_x	150
Variimorda mendax	Méquignon, 1946	Mordellidae	4	2	s_m	b_w	h_t	f_x	100
Vibidia duodecimguttata	(Poda von Neuhaus, 1761)	Coccinellidae	8	6	s_s	b_w	h_v	f_z	200
Vincenzellus ruficollis	(Panzer, 1794)	Salpingidae	5	4	s_s	b_w	h_t	f_z	150
Wanachia triguttata	(Gyllenhal, 1810)	Melandryidae	7	5	s_xs	b_w	h_t	f_m	100
Xantholinus elegans	(A. G. Olivier, 1795)	Staphylinidae	5	4	s_m	b_o	h_b	f_z	100
Xantholinus linearis	(A. G. Olivier, 1795)	Staphylinidae	11	10	s_m	b_e	h_b	f_z	300
Xantholinus longiventris	Heer, 1839	Staphylinidae	4	4	s_m	b_e	h_b	f_z	150
Xantholinus tricolor	(Fabricius, 1787)	Staphylinidae	7	6	s_m	b_w	h_b	f_z	300
Xyleborinus saxesenii	(Ratzeburg, 1837)	Curculionidae	4	4	s_xs	b_w	h_t	f_m	150
Xyleborus monographus	(Fabricius, 1792)	Curculionidae	7	5	s_xs	b_w	h_t	f_m	300
Xyletinus ater	(Creutzer, 1796)	Ptinidae	7	6	s_s	b_w	h_t	f_x	200
Xylocleptes bispinus	(Duftschmid, 1825)	Curculionidae	11	7	s_xs	b_o	h_t	f_x	300
Zabrus tenebrioides	(Goeze, 1777)	Carabidae	4	4	s_l	b_o	h_b	f_p	200
Zacladus exiguus	(A. G. Olivier, 1807)	Curculionidae	4	3	s_xs	b_o	h_v	f_p	100
Zacladus geranii	(Paykull, 1800)	Curculionidae	8	8	s_xs	b_o	h_v	f_p	300
Zeugophora flavicollis	(Marsham, 1802)	Megalopodidae	4	4	s_s	b_w	h_v	f_p	100
Zorochros dufouri	(Buysson, 1900)	Elateridae	7	5	s_s	b_f	h_b	f_e	300
Zorochros meridionalis	(Laporte, 1840)	Elateridae	4	4	s_xs	b_o	h_b	f_e	300
